# Microwave-assisted synthesis of biologically relevant steroidal 17-*exo*-pyrazol-5'-ones from a norpregnene precursor by a side-chain elongation/heterocyclization sequence

**DOI:** 10.3762/bjoc.14.236

**Published:** 2018-10-08

**Authors:** Gergő Mótyán, László Mérai, Márton Attila Kiss, Zsuzsanna Schelz, Izabella Sinka, István Zupkó, Éva Frank

**Affiliations:** 1 Department of Organic Chemistry, University of Szeged, Dóm tér 8, H-6720 Szeged, Hungary; 2 Department of Pharmacodynamics and Biopharmacy, University of Szeged, Eötvös u. 6, H-6720 Szeged, Hungary

**Keywords:** antiproliferative activity, Knorr reaction, microwave, pyrazol-5-ones, steroids

## Abstract

Multistep syntheses of novel 17β-pyrazol-5'-ones in the Δ^5^-androstane series were efficiently carried out from pregnenolone acetate. A steroidal 17-carboxylic acid was first synthesized as a norpregnene precursor by the bromoform reaction and subsequent acetylation. Its CDI-activated acylimidazole derivative was then converted to a β-ketoester containing a two carbon atom-elongated side chain than that of the starting material. A Knorr cyclization of the bifunctional 1,3-dicarbonyl compound with hydrazine and its monosubstituted derivatives in AcOH under microwave heating conditions led to the regioselective formation of 17-*exo*-heterocycles in good to excellent yields. The suppression of an acid-catalyzed thermal decarboxylation of the β-ketoester and thus a significant improvement in the yield of the desired heterocyclic products could be achieved by the preliminary liberation of the arylhydrazines from their hydrochloride salts in EtOH in the presence of NaOAc. The reaction rates were found to depend on the electronic character of the substituent present in the phenylhydrazine applied. The antiproliferative activities of the structurally related steroidal pyrazol-5'-ones and their deacetylated analogs were screened on three human adherent breast cancer cell lines (MCF7, T47D and MDA-MB-231): the microculture tetrazolium assay revealed that some of the presented derivatives exerted cell growth inhibitory effects on some of these cell lines comparable to those of the reference compound, cisplatin.

## Introduction

17-*exo*-Heterocyclic androstanes with five or six-membered heterocyclic rings connected directly to C-17 of the sterane core represent a remarkable subclass of semisynthetic sex hormone analogs in consequence of their dual pharmacological importance. A number of these derivatives display an inhibitory effect on 17α-hydroxylase-C_17,20_-lyase (P450_17α_) enzyme, which, acting as an important regulator, plays an essential role in the endogenous production of androgen hormones, and therefore, in the development of prostate cancer [1]. According to extensive structure–activity relationship and docking studies, a potent steroidal inhibitor should possess certain structural characteristics for efficient P450_17α_ inhibition [1–3], such as (i) a five or six-membered non-bulky heterocycle containing O, N or S atoms attached to position C-17 of the sterane skeleton with the lone electron pairs capable of coordinating with the heme iron at the active site; (ii) a N atom at either position 3′ or 4′ relative to the atom through which the heterocyclic ring is connected to the sterane framework; (iii) a hydroxy or keto group at C-3 and (iv) the presence of a C_16_–C_17_ double bond, which facilitates the inhibitory effect but is not an essential requirement. Some 17-heterocycle-substituted androstanes, even those that lack the structural features described above, are also known to display cytotoxic effects on diverse cancer cells by inducing a disturbance in the cell cycle and promoting apoptosis without affecting normal cellular proliferation [4–5]. In these latter cases, detailed structural criteria are still not available owing to little information about the mode of action of these derivatives.

Amongst steroidal 17-*exo*-heterocycles, those containing a pyrazole heteroaromatic ring are of special relevance with respect to the above-mentioned bioactivities [6–9]. Interestingly, so far only a few examples of compounds in which a pyrazolone moiety is attached to the sterane skeleton have been published, but not to C-17 [10]. Nevertheless, this heterocyclic scaffold is also an important building block in many clinically relevant drugs, agrochemicals, dyes, pigments and chelating agents [11–13], and therefore its introduction to C-17 of androstanes may be of interest from a pharmacological point of view.

The first and probably most frequently used method for the synthesis of pyrazolones is based on the Knorr condensation of β-ketoesters with substituted or unsubstituted hydrazines. However, these reactions often suffer from certain disadvantages, such as the necessity of high temperature or prolonged reaction time and low yields of the desired products [14]. A rate acceleration and yield improvement could be achieved in some cases by performing the reactions under microwave (MW) conditions [15–17]. Especially with respect to pyrazol-5-ones, keto–enol tautomerism can be challenging and is of special importance in biological systems, chemical reactivity, and molecular recognition [18]. The tautomeric equilibrium in solution is strongly influenced by the substitution pattern of the heterocyclic ring, the polarity and protic nature of the solvent and, although to a lesser extent, by the temperature and concentration [19].

In view of the above-mentioned reasons, the main objective of the present study was to design and carry out the preparation of novel steroidal heterocycles containing pyrazol-5-one moieties attached to C-17 of the sterane core, using both conventional heating and MW irradiation. The reaction conditions were optimized in order to improve the yields of the desired products and the influences of substituents of the hydrazine reagents investigated. The in vitro antiproliferative activities of the synthesized compounds were also determined on three human breast malignant cell lines (MCF7, T47D, and MDA-MB-231) by means of 3-(4,5-dimethylthiazol-2-yl)-2,5-diphenyltetrazolium bromide (MTT) assay [20]. Furthermore, the most promising molecules were additionally tested on mouse fibroblasts (NIH-3T3) in order to obtain preliminary results concerning the cancer selectivity of the selected agents.

## Results and Discussion

### Synthetic studies

**Scheme 1:**
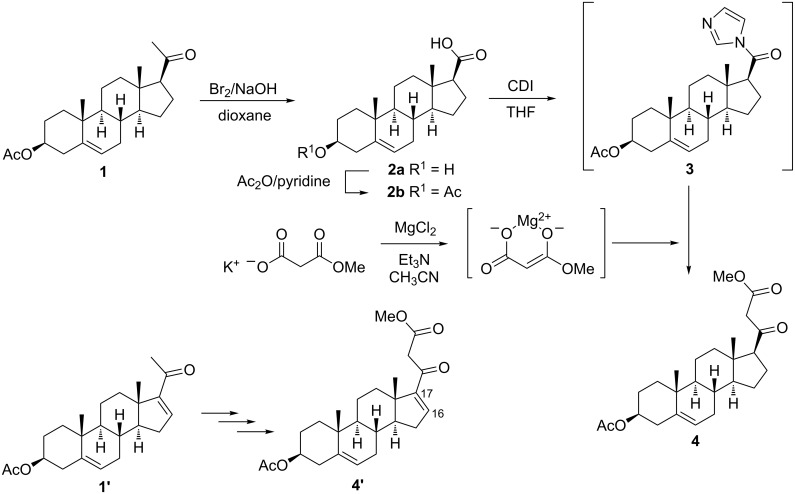
Multistep synthesis of steroidal β-ketoesters **4** and **4'** from pregnenolone acetate (**1**) and pregnadienolone acetate (**1'**).

The steroidal β-ketoester precursor **4**, suitable for the attempted heterocyclization reaction with hydrazines was synthesized from commercially available pregnenolone acetate (**1**) via a multistep sequence (Scheme 1). First compound **1** was converted to the 17β-carboxylic acid **2b** by the bromoform reaction and subsequent acetylation according to well-known literature procedures [21–23]. After the activation of **2b** with 1,1′-carbonyldiimidazole (CDI) as coupling reagent in THF, the magnesium enolate of malonic acid half ester, prepared in situ from potassium methyl malonate, MgCl_2_ and triethylamine in acetonitrile, was added [24–25]. The acylation of magnesium methyl malonate by the preformed imidazole **3** led to the desired bifunctional starting material **4** in good yield (79%).

Analogously, β-ketoester **4'** could be obtained from pregnadienolone acetate **1'** through a Δ^5,16^-carboxylic acid intermediate [23,26] under identical conditions albeit in disappointing low yield (33%) which is presumably caused by the decreased propensity of the conjugated carbonyl compound to react with the magnesium enolate. Although the presence of a C_16_–C_17_ double bond as in **4'** is assumed to be beneficial for a P450_17α_-inhibitory effect, further transformations of this compound were abandoned because of the insufficient yield and its potential tendency to react with monosubstituted hydrazines – not only with its β-ketoester moiety to give 17-*exo*-heterocycles, but also with its enone part to provide ring D-condensed pyrazolines [27].

**Scheme 2:**
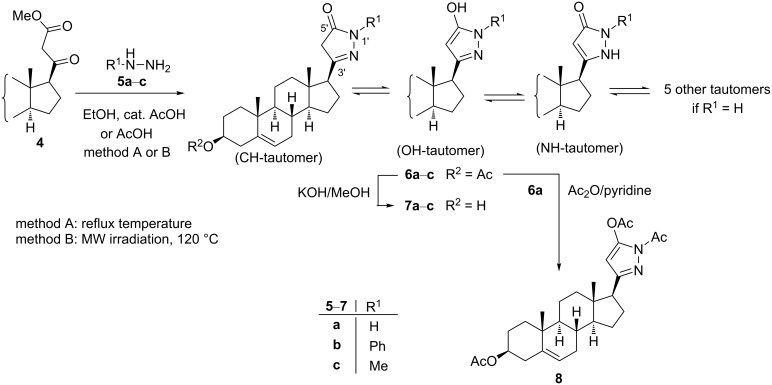
Cyclization of compound **4** with hydrazine hydrate (**5a**), phenylhydrazine (**5b**) and methylhydrazine (**5c**).

Therefore, the ring-closure reactions of **4** with unsubstituted and monosubstituted hydrazines as binucleophilic reagents were investigated next. First, compound **4** was reacted with hydrazine hydrate (**5a**) in refluxing ethanol containing a catalytic amount of AcOH (Scheme 2).

TLC monitoring of the reaction indicated full conversion of **4** within 4 h reaction time to afford a fairly polar product insoluble or only slightly soluble in all commonly used NMR solvents. However, a subsequent derivatization with acetic anhydride in pyridine to afford **8**, allowed its structure verification indirectly. This derivatization did not only improve the solubility of the compound, but also eliminated the possibility of prototropic tautomerism through acetylation of both the amino and hydroxy groups present in the heterocyclic ring in **6a**. The ^1^H and ^13^C NMR spectroscopical analysis confirmed the structure of **8**, which at the same time supported the formation of **6a** in a yield of 84% during the previous reaction step. While N-unsubstituted pyrazolones such as **6a** theoretically may have eight tautomeric forms [28], their N(1')-substituted derivatives, lacking a functionality on pyrazole C-4, can only exist in three equilibrating tautomers (OH, CH and NH) [18,29]. Therefore, further heterocyclizations of **4** were performed with monosubstituted hydrazine derivatives. The reaction with phenylhydrazine (**5b**) was completed within 7 h in refluxing EtOH in the presence of an acid catalyst. A reduction of the reaction time to 3 h could be achieved by changing the solvent to AcOH affording the desired product **6b** in high yield (86%, Scheme 2). On the other hand, the reaction of **4** with methylhydrazine (**5c**) required a longer reaction time (5 h) in refluxing AcOH to furnish the purified product (**6c**) in a diminished yield (61%). This may be attributed to the weaker nucleophilic character of the external N compared to the internal one in **5c** [27], in contrast to phenylhydrazine (**5b**), making the first condensation step more difficult. The regioselectivity of the reactions with monosubstituted hydrazines is controlled by the higher reactivity of the ketone moiety over the ester towards nucleophiles, and the least hindered terminal nitrogen atom of the binucleophiles. Both reactions were repeated in AcOH under microwave conditions at 120 °C furnishing products **6b** and **6c** within shorter time (20 min and 40 min), however, without a substantial improvement in the yields.

Since commercially available arylhydrazine hydrochlorides were intended to be applied for further transformations, the reactions of **4** with **5b**·HCl using an equivalent amount of NaOAc in AcOH, both under conventional heating and MW irradiation, were also carried out. Although similar reaction conditions have been described in the literature for the reactions of methyl acetoacetate with arylhydrazine hydrochlorides to afford the corresponding pyrazol-5-ones in 50–70% yields within 5–10 h [30], no full conversion of **4** could be achieved. Even after refluxing the mixture for 24 h the desired product **6b** was obtained only in low yield (≈30%). At the same time, the use of the MW-assisted method at 120 °C shortened the conversion time significantly (80 min). However, in addition to **6b** (≈50%), the conventional, and even more the MW-promoted transformations led to a considerable amount (20–25%) of pregnenolone acetate (**1**) as a byproduct. The latter is thought to be produced by an acid-catalyzed thermal decarboxylation of **4** during the relatively long heating period. Further the unwanted side reaction was attributed to the poor solubility of **5b**·HCl in AcOH resulting in a heterogeneous reaction mixture even at high temperature and therefore a slow liberation of **5b** from its salt upon the addition of NaOAc. In order to circumvent this issue, the reaction was repeated with in situ-liberated phenylhydrazine (**5b**) by dissolving **5b**·HCl and NaOAc in a small amount of EtOH under mild heating for 10 min. To this solution, containing NaCl as the only solid substance, the steroidal dicarbonyl compound **4** dissolved in AcOH was added. The so obtained mixture was then irradiated in a MW reactor at 120 °C for 20 min. Under these conditions, the corresponding product **6b** was obtained in 85% yield after chromatographic purification without notable formation of **1**.

After optimizing the conditions for the MW-assisted synthesis of **6b** from **4** with **5b**·HCl, analogous heterocyclization reactions were carried out with different substituted phenylhydrazine hydrochlorides **5d**–**j**. All reactions furnished the corresponding 17-*exo*-heterocycles **6d**–**j** in good to excellent yields (83–92%, Table 1).

**Table 1:** Synthesis of steroidal *N*(1')-aryl-substituted pyrazol-5'-ones^a^.

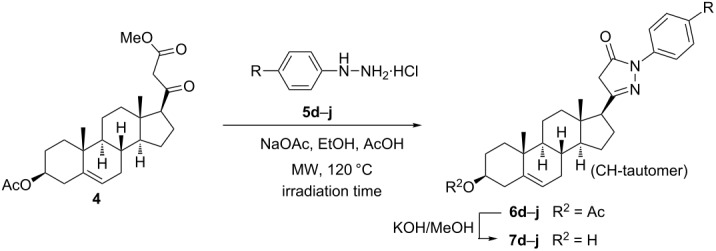					

entry	arylhydrazine hydrochloride	R	pyrazol-5-one	irradiation time (min)	yield^b^ (%)

1	**5d**	Me	**6d**	10	86
2	**5e**	iPr	**6e**	10	90
3	**5f**	*t*-Bu	**6f**	10	87
4	**5g**	OMe	**6g**	5	92
5	**5h**	F	**6h**	30	85
6	**5i**	Cl	**6i**	30	83
7	**5j**	Br	**6j**	30	85

^a^Reagents and conditions: arylhydrazine hydrochloride **5d**–**j**·HCl (1.2 equiv), NaOAc (1.2 equiv), EtOH (10 mL), 40 °C, 10 min, then compound **4** (1.0 mmol) in AcOH (20 mL), MW, 120 °C, 5–30 min. ^b^After purification by column chromatography.

The electronic features of the substituents on the aromatic ring of **5** had a notable influence on the reaction rates. The ring-closure of **4** with **5d**–**g** containing electron-donating groups (Table 1, entries 1–4) took place within shorter reaction times (5–10 min) compared to phenylhydrazine (**5b**). On the other hand the presence of electron-withdrawing halogens on the aromatic ring of **5** (Table 1, entries 5–7) lengthened the reaction time to 30 min. This observation can be explained by the enhanced or diminished nucleophilic character of the nitrogen atoms caused by the different groups on the aromatic ring in **5**, favoring or hampering their nucleophilic attack during the intermolecular heterocyclization. In order to enlarge the compound library available for pharmacological studies, the 3β-OH analogs **7a**–**j** of the primary products **6a**–**j** were also synthesized through simple alkaline deacetylation (Scheme 2, Table 1).

**Figure 1:**
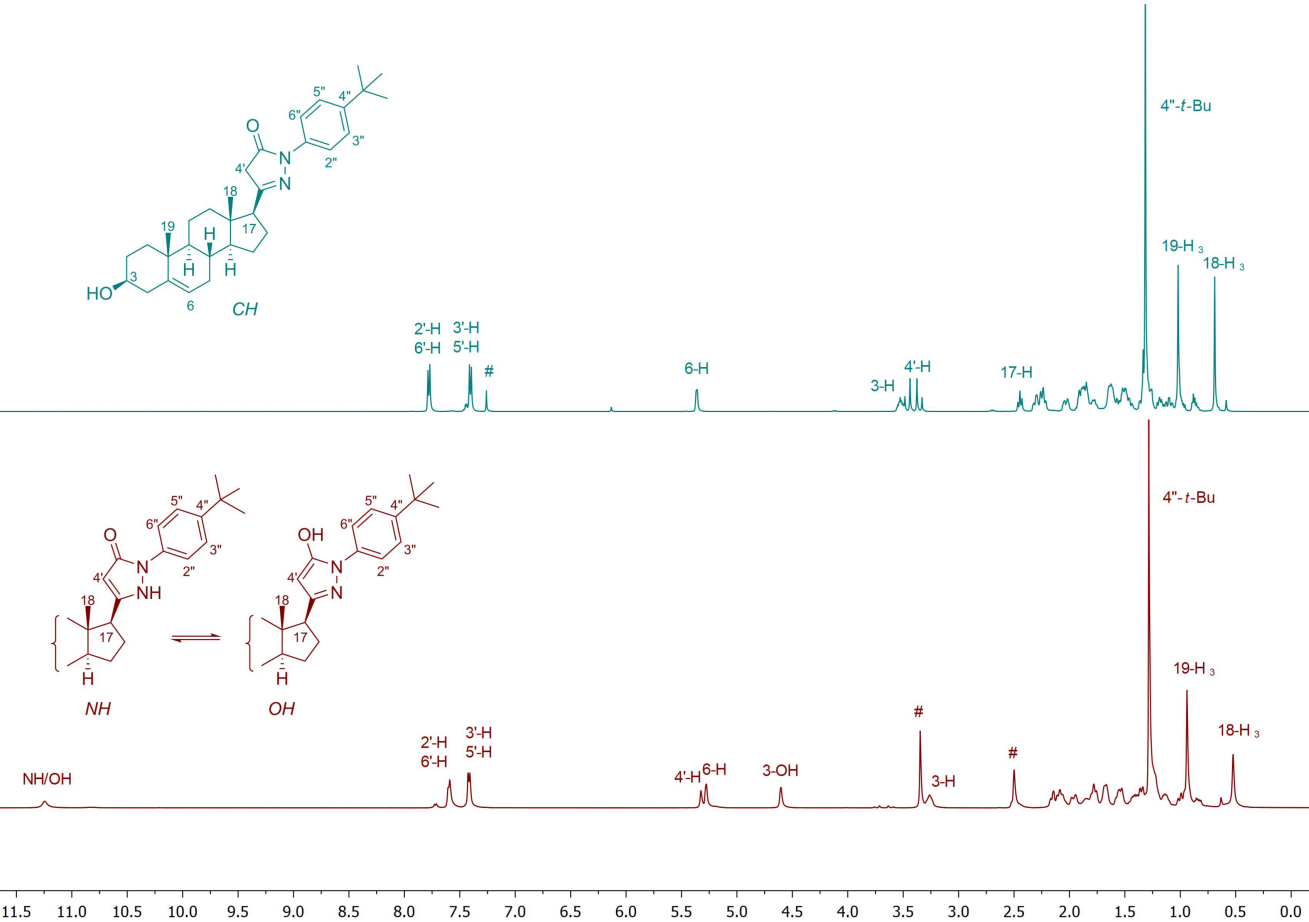
^1^H NMR spectra of compound **7f** in CDCl_3_ (top; # solvent signal) and in DMSO-*d*_6_ (bottom; # solvent signal).

The structures of all synthesized compounds were characterized by ^1^H and ^13^C NMR spectroscopy supplemented by IR and MS measurements. The dependence of the tautomeric equilibrium on the polarity of the applied solvent was observed during the NMR experiments. For example, in the ^1^H NMR spectrum of compound **7f** recorded immediately after dissolution in CDCl_3_, the pyrazolone heterocyclic ring mainly exists as the CH-tautomeric form (Figure 1). The characteristic signals of the 4'-methylene hydrogens appear at 3.36 and 3.46 ppm in the ^1^H NMR spectrum. However, in the more polar solvent DMSO-*d*_6_, the equilibrium mixture of the NH- and OH-tautomers of **7f** predominates. As the activation barrier between these latter isomers is low and their interconversion is rapid on the NMR timescale, only average signals can be observed for the 4'-H and NH/OH protons [31].

### Pharmacological studies

The pharmacological activities of the synthesized 17-*exo*-heterocycles **6a**–**j** and **7a**–**j** were studied in vitro. Their antiproliferative effects were determined by means of the MTT assay [20] on a panel of adherent breast cancer cell lines (MCF7, T47D and MDA-MB-231) after treatment for 72 h (Table 2). All compounds were initially screened at 10 and 30 μM concentrations and for those compounds that elicited growth inhibition of at least 50% at 10 μM and around 90% at 30 μM, IC_50_ values were calculated by using a set of dilutions (Figure S1 in Supporting Information File 1). The viability assays were repeated with the most potent agents **6h**, **7f**, **7i** and **7j** against NIH-3T3 mouse fibroblasts to generate preliminary data concerning the cancer selectivity. Since the molecular site of action of the tested compounds is not known, cisplatin (a clinically used DNA-binding anticancer agent) was applied as the reference. The results indicated that the 3β-acetates **6a**–**j** exhibited weak or only modest antiproliferative activities, typically eliciting 20–30% growth inhibition at 10 μM. However, the *p*-fluoro derivative **6h**, proved to be more effective on MCF7 and T47D cells. The deacetylated analogs **7a**–**j** generally inhibited cancer cell growth more efficiently. The character of the substituent on the aromatic ring was crucial for the antiproliferative actions on the different cell lines. A *tert-*butyl group at the *para* position (**7f**) appeared favorable against T47D cells resulting in an IC_50_ value lower than that of the reference cisplatin. While the chloro and bromo-substituted derivatives exerted more pronounced effects on MCF7 cells in their 3-OH form (**7i** and **7j**), the fluoro compound **7h** proved to be less active than its ester **6h**. Although the performed viability assay on animal fibroblasts cannot be considered as an appropriate toxicological evaluation, the obtained results are promising and point to a less growth inhibiting action of the compounds on fibroblasts than on cancer cells. All of them displayed less than 20% growth inhibition at 10 μM and their calculated IC_50_ values proved to be higher than those obtained on the malignant cell lines.

**Table 2:** Antiproliferative effects of the synthesized compounds on gynecological cell lines and NIH-3T3 fibroblasts.^a^

compound	conc. (μM)	inhibition of cell proliferation (%) ± SEM [calculated IC_50_ (μM)]			

		MCF7	MDA-MB-231	T47D	NIH-3T3

**6a**	10	25.7 ± 1.1	–	–	n.d.
	30	70.0 ± 1.0	30.9 ± 2.0	58.9 ± 1.0	

**6b**	10	–	–	38.2 ± 1.5	n.d.
	30	76.8 ± 0.3	72.1 ± 0.4	90.9 ± 0.6	

**6c**	10	–	–	–	n.d.
	30	52.2 ± 2.8	26.8 ± 1.2	83.8 ± 1,3	

**6d**	10	–	–	–	n.d.
	30	35.8 ± 1.1	–	51.4 ± 1.7	

**6e**	10	–	–	–	n.d.
	30	–	–	41.7 ± 2.6	

**6f**	10	–	–	–	n.d.
	30	73.8 ± 0.6	29.3 ± 3.0	72.2 ± 1.7	

**6g**	10	–	–	–	n.d.
	30	47.4 ± 1.5	–	60.7 ± 2.2	

**6h**	10	65.1 ± 0.9	–	74.9 ± 2.4	–
	30	95.1 ± 1.1	90.8 ± 0.5	84.2 ± 1.6	88.3 ± 0.8
		[9.0]		[6.5]	[18.1]

**6i**	10	–	–	22.2 ± 1.3	n.d.
	30	87.8 ± 0.7	29.2 ± 1.2	78.0 ± 0.6	

**6j**	10	31.0 ± 1.7	–	31.1 ± 2.2	n.d.
	30	86.4 ± 1.4	27.0 ± 1.0	77.5 ± 1.1	

**7a**	10	–	–	57.0 ± 0.9	n.d.
	30	–	–	65.8 ± 1.3	

**7b**	10	–	–	–	n.d.
	30	85.8 ± 1.0	40.6 ± 1.7	89.6 ± 0.7	

**7c**	10	–	–	–	n.d.
	30	23.1 ± 2.6	57.9 ± 2.0	–	

**7d**	10	–	–	31.1 ± 1.7	n.d.
	30	27.9 ± 2.7	–	60.3 ± 1.9	

**7e**	10	–	–	–	n.d.
	30	66.3 ± 1.5	–	95.8 ± 0.2	

**7f**	10	21.1 ± 2.8	31.3 ± 1.3	83.1 ± 1.3	–
	30	95.8 ± 0.3	89.6 ± 0.8	87.5 ± 0.5	84.6 ± 1.3
				[4.3]	[18.2]

**7g**	10	–	–	22.3 ± 1.3	n.d.
	30	40.9 ± 1.6	39.6 ± 1.7	55.7 ± 2.2	

**7h**	10	–	–	–	n.d.
	30	91.3 ± 0.8	96.8 ± 0.2	85.2 ± 1.1	

**7i**	10	66.8 ± 1.7	31.3 ± 1.5	32.8 ± 1.3	–
	30	96.3 ± 0.2	96.8 ± 0.2	87.8 ± 0.5	94.0 ± 0.5
		[6.9]			[15.3]

**7j**	10	58.6 ± 1.2	42.0 ± 0.8	48.3 ± 1.9	–
	30	89.5 ± 1.1	96.1 ± 0.2	85.5 ± 1.2	91.1 ± 1.0
		[8.1]			[17.3]

cisplatin	10	66.9 ± 1.8	–	51.0 ± 2.0	94.2 ± 0.4
	30	96.8 ± 0.4	71.5 ± 1.2	55.0 ± 1.5	96.4 ± 0.2
		[5.8]	[19.1]	[9.8]	[3.2]

^a^Compounds eliciting less than 20% inhibition of proliferation were considered ineffective and the exact results are not given, for simplicity. n.d.: not determined.

## Conclusion

In summary, a microwave-assisted one-pot method for the facile and efficient synthesis of novel steroidal 17-*exo*-pyrazol-5'-ones from a β-ketoester precursor with arylhydrazine hydrochlorides has been developed. An acid-catalyzed thermal decarboxylation of the starting material as an unwanted side reaction could be avoided by applying a one-pot two-step protocol involving the in situ liberation of the reagent from its salt in EtOH followed by the heterocyclization reaction through the addition of AcOH. Some of the presented compounds **6h**, **7f**, **7i** and **7j** exerted considerable antiproliferative activity with promising cancer selectivity on a panel of human breast cancer cell lines. This indicates that the pyrazolone heterocyclic ring at the 17β position is a promising scaffold for the design of anticancer agents of the Δ^5^ androstene series.

## Supporting Information

Experimental procedures for compounds **6a**–**j**, **7a**–**j** and **8**, their ^1^H, ^13^C NMR, MS, IR, elemental analysis data and the copies of their NMR spectra.

File 1 Experimental part.
