# Supplementary material for: Microwave-assisted synthesis of biologically relevant steroidal 17-exo-pyrazol-5'-ones from a norpregnene precursor by a side-chain elongation/heterocyclization sequence
Source: Beilstein J Org Chem. 2018 Oct 8;14:2589–96. doi: 10.3762/bjoc.14.236 (PMC6204839; doi:10.3762/bjoc.14.236)
Supplement: File 1 — Experimental part. [file Beilstein_J_Org_Chem-14-2589-s001.pdf]

Supporting Information
for
Microwave-assisted synthesis of biologically relevant steroidal 17-*exo*-pyrazol-5'-ones from a norpregnene precursor by a side-chain elongation/heterocyclization sequence

Gergő Mótyán[1], László Mérai[1], Márton Attila Kiss[1], Zsuzsanna Schelz[2], Izabella Sinka[2], István Zupkó[2] and Éva Frank*[1]

Address: [1]Department of Organic Chemistry, University of Szeged, Dóm tér 8, H-6720 Szeged, Hungary and [2]Department of Pharmacodynamics and Biopharmacy, University of Szeged, Eötvös u. 6, H-6720 Szeged, Hungary

Email: Éva Frank* - frank@chem.u-szeged.hu

* Corresponding author

Experimental part

Table of contents

1. General experimental methods and equipment S2
2. Characterization of 3β-acetoxy-20-oxopregn-5,16-dien-21-carboxylic acid methyl ester (**4'**) S3
3. Synthesis and characterization of compounds **6a**–**j** S3
4. Synthesis and characterization of compounds **7a**–**j** S9
5. Synthesis and characterization of compound **8** S14
6. Pharmacological methods S15
7. Figure S1: Concentration–response curves of most effective molecules S17
8. NMR spectra collection S18

1. General experimental methods and equipment

All solvents were distilled and dried prior to use. Reagents and materials were obtained from commercial suppliers and used without purification. The reactions under microwave irradiation were carried out with a CEM Corporation Focused Microwave System, Model Discover SP. Melting points (mp) were determined on an SMS Optimelt digital apparatus. Elemental analysis data were obtained with a Perkin Elmer CHN analyzer model 2400 and FTIR spectra were recorded on a FT/IR-4700 spectrometer (Jasco) using ATR. Infrared absorbance is reported in cm^{-1}. ^{1}H NMR spectra were obtained at room temperature in $CDCl_3$ or in DMSO–d_6 solution at 500 MHz (Bruker DRX 500) and ^{13}C NMR spectra at 125 MHz with the same instrument. Chemical shifts are reported in ppm (δ scale), and coupling constants (J) in Hz. The multiplicities of the ^{1}H resonance peaks are indicated as a singlet (s), a broad singlet (bs), a doublet (d), a triplet (t) or a multiplet (m). ^{13}C NMR spectra are ^{1}H-decoupled. For the determination of multiplicities, the *J*-MOD pulse sequence was used. Automated flow injection analyses were performed by using an HPLC/MSD system. The system comprised an Agilent 1100 micro vacuum degasser, a quaternary pump, a microwell plate autoinjector and a 1946A MSD equipped with an electrospray ion (ESI) source operated in the positive ion mode. The ESI parameters were: nebulizing gas N_2, at 35 psi; drying gas N_2, at 350 °C and 12 L/min; capillary voltage (V_{Cap}) 3000 V; and fragmentor voltage 70 V. The MSD was operated in scan mode with the mass range m/z 60–620. Samples (0.2 μL) were injected with an automated needle wash directly into the solvent flow (0.3 mL/min) of $MeCN/H_2O$ 70:30 (v/v) supplemented with 0.1% formic acid. The system was controlled by Agilent LC/MSD Chemstation software. All solvents were distilled immediately prior to use. The reactions were monitored by TLC on Kieselgel-G (Merck Si 254 F) layers (0.25 mm thick); solvent systems (ss): (A) $EtOAc/CH_2Cl_2$ 2:98 (v/v) (B) EtOAc/hexane 20:80 (v/v) (C) EtOAc/hexane 30:70 (v/v), (D) EtOAc/hexane 50:50 (v/v), (E) $MeOH/CH_2Cl_2$ 5:95 (v/v), (F) $MeOH/CH_2Cl_2$ 10:90 (v/v). The spots were detected by spraying with 5% phosphomolybdic acid in 50% aqueous phosphoric acid. The R_f values were determined for the spots observed by illumination at 254 and 365 nm.
Flash chromatography: Merck silica gel 60, 40–63 μm.

2. Characterization of 3β-acetoxy-20-oxopregn-5,16-dien-21-carboxylic acid methyl ester (4')

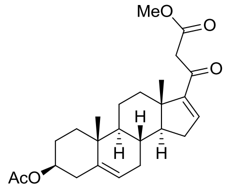

White solid, mp: 106–109 °C, R_f = 0.60 (ss A), IR (cm^{-1}): 2933, 1742, 1725, 1659, 1581, 1253, 1234, 1037, ^{1}H NMR ($CDCl_3$, 500 MHz): δ 0.92 (s, 3H, 18-H_3), 1.02 (m, 1H), 1.05 (s, 3H, 19-H_3), 1.13 (m, 1H), 1.36 (m, 1H), 1.45 (m, 1H), 1.52-1.74 (overlapping m, 5H), 1.85 (m, 2H), 1.98 (m, 1H), 2.02 (s, 3H, Ac-CH_3), 2.07 (m, 1H), 2.28-2.39 (overlapping m, 4H), 3.66 (d, 2H, J = 3.84 Hz, 21-H_2), 3.72 (s, 3H, OMe), 4.60 (m, 1H, 3-H), 5.38 (m, 1H, 6-H), 6.74 (s-like m, 16-H), ^{13}C NMR ($CDCl_3$, 125 MHz): δ 15.8 (C-18), 19.3 (C-19), 20.7 (CH_2), 21.5 (Ac-CH_3), 27.8 (CH_2), 30.3 (CH), 31.6 (CH_2), 32.7 (CH_2), 34.6 (CH_2), 36.9 (C-10), 37.0 (CH_2), 38.3 (CH_2), 46.4 (CH_2), 46.5 (C-13), 50.5 (CH), 52.4 ((OMe), 56.4 (CH), 74.0 (C-3), 122.0 (C-6), 140.4 (C-5), 146.1 (C-16), 154.7 (C-17), 168.2 (C-22), 170.6 (Ac-CO), 190.5 (C-20), ESI-MS: 415 $[M+H]^+$; Anal. Calcd for $C_{25}H_{34}O_5$ C, 72.44; H, 8.27. Found: C, 72.52; H, 8.18.

3. Synthesis and characterization of compounds 6a–j

3.1. 3β-Acetoxy-17β-[5'-hydroxy-1'*H*-pyrazol-3'-yl]androst-5-ene (**6a**)

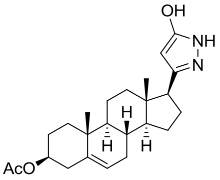

Compound **4** (333 mg, 0.80 mmol) and hydrazine hydrate (**5a**, 0.12 mL, 2.5 mmol) were dissolved in EtOH (10 mL) and 2 drops of glacial acetic acid were added. The reaction mixture was kept at reflux temperature for 4 h, and then poured into cold water (10 mL). The precipitate was filtered and the crude product was purified by column chromatography with MeOH/CH_2Cl_2 30:70 to give **6a** (268 mg, 84%) as white solid.

Mp: 277–280 °C, IR (cm^{-1}): 3318, 3182, 2904, 1729, 1595, 1245, 1033, ^{1}H NMR (DMSO-d_6, 500 MHz): δ 0.48 (s, 3H, 18-H_3), 0.97 (s, 3H, 19-H_3), 1.98 (s, 3H, 3-OAc), 4.45 (m, 1H, 3-H), 5.23 (s, 4′-H), 5.36 (m, 1H, 6-H), 9.25 (bs, NH/OH), 11.17 (bs, NH/OH), ESI-MS: 399 $[M+H]^+$; Anal. Calcd for $C_{24}H_{34}N_2O_3$ C, 72.33; H, 8.60. Found: C, 72.25; H, 8.49.

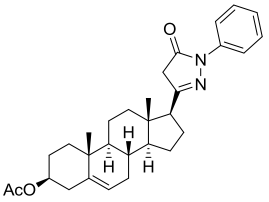

3.2. 3β-Acetoxy-17β-[5'-hydroxy-1'-phenyl-1'*H*-pyrazol-3'-yl]androst-5-ene (**6b**)

<u>Method A</u>: Compound **4** (333 mg, 0.80 mmol) and phenylhydrazine (**5b**, 0.08 mL, 0.80 mmol) were dissolved in AcOH (10 mL). The reaction mixture was kept at reflux temperature for 3 h and then poured into cold water (10 mL). The resulting yellow precipitate was filtered and the crude product purified by column chromatography with EtOAc/hexane 20:80 to give **6b** (327 mg, 86%) as white solid.

<u>Method B</u>: Compound **4** (333 mg, 0.80 mmol) and phenylhydrazine (**5b**, 0.10 mL, 1.0 mmol) were dissolved in AcOH (5 mL). The reaction mixture was irradiated at 120 °C for 20 min (max. power: 150 W), and then poured into cold water (10 mL). The resulting yellow precipitate was filtered and the crude product purified by column chromatography with EtOAc/hexane 20:80 to give **6b** (334 mg, 88%) as white solid.

Mp: 179–180 °C, R_f = 0.38 (ss C), IR (cm^{-1}): 2950, 1714, 1598, 1499, 1239, 1024, 756, 691, ^{1}H NMR ($CDCl_3$, 500 MHz): δ 0.70 (s, 3H, 18-H_3), 1.03 (s, 3H, 19-H_3), 2.03 (s, 3H, Ac-CH_3), 2.46 (t, 1H, *J* = 9.5 Hz, 17-H), 3.36 (d, 1H, *J* = 23.0 Hz) and 3.47 (d, 1H, *J* = 23.0 Hz): 4′-CH_2, 4.60 (m, 1H, 3-H), 5.39 (m, 1H, 6-H), 7.14 (m, 1H, 4"-H), 7.38 (m, 2H, 3"-H and 5"-H), 7.89 (d, 2H, *J* = 8.5 Hz, 2"-H and 6"-H), ^{13}C NMR ($CDCl_3$, 125 MHz): δ 13.6 (C-18), 19.5 (C-19), 21.0 (CH_2), 21.6 (Ac-CH_3), 23.8 (CH_2), 24.5 (CH_2), 27.8 (CH_2), 31.8 (CH_2), 32.1 (CH), 36.8 (C-10), 37.1 (CH), 38.2 (CH_2), 38.3 (CH_2), 43.2 (CH_2), 44.1 (C-13), 50.1 (CH), 52.7 (CH), 56.4 (CH), 73.9 (C-3), 118.9 (2C, C-2" and C-6"), 122.4 (C-6), 125.0 (C-4"), 128.9 (2C, C-3" and C-5"), 138.4 (C-1"), 139.9 (C-5), 160.2 (C-3′), 170.7 (Ac-CO), 170.8 (C-5′), ESI-MS: 475 $[M+H]^+$; Anal. Calcd for $C_{30}H_{38}N_2O_3$ C, 75.92; H, 8.07. Found: C, 76.06; H, 8.15.

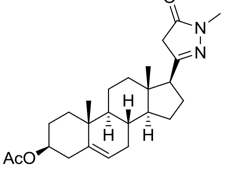

3.3. 3β-Acetoxy-17β-[5'-hydroxy-1'-methyl-1'*H*-pyrazol-3'-yl]androst-5-ene (**6c**)

<u>Method A</u>: Compound **4** (333 mg, 0.80 mmol) and methylhydrazine (**5c**, 0.05 mL, 1.0 mmol) were dissolved in AcOH (10 mL). The reaction mixture was kept at reflux temperature for 5 h and then poured into cold water (10 mL). The precipitate was filtered and the crude product purified by column chromatography with MeOH/CH_2Cl_2 3:97 to give **6c** (201 mg, 61%) as white solid.

Method B: Compound **4** (333 mg, 0.80 mmol) and methylhydrazine (**5c**, 0.05 mL, 1.0 mmol) were dissolved in AcOH (5 mL). The reaction mixture was irradiated at 120 °C for 40 min (max. power: 150 W), and then poured into cold water (10 mL). The resulting precipitate was filtered and the crude product purified by column chromatography MeOH/CH_2Cl_2 3:97 to give **6c** (215 mg, 65%) as white solid.

Mp.: 228–230 °C, R_f = 0.48 (ss E), IR (cm^{-1}): 2940, 1730, 1558, 1237, 1032, ^{1}H NMR ($CDCl_3$, 500 MHz): δ 0.63 (s, 3H, 18-H_3), 1.01 (s, 3H, 19-H_3), 2.02 (s, 3H, 3-Ac-CH_3), 3.11 (d, 1H, J = 23.0 Hz) and 3.22 (d, 1H, J = 23.0 Hz): 4′-CH_2, 3.28 (s, 3H, 1′-CH_3), 4.59 (m, 1H, 3-H), 5.36 (m, 1H, 6-H), ^{13}C NMR ($CDCl_3$, 125 MHz): δ 13.5 (C-18), 19.4 (C-19), 21.0 (CH_2), 21.5 (3-Ac-CH_3), 23.7 (CH_2), 24.4 (CH_2), 27.8 (CH_2), 31.3 (CH), 31.8 (CH_2), 32.1 (CH), 36.8 (C-10), 37.1 (CH_2), 38.2 (CH_2), 38.3 (CH_2), 41.5 (CH_2), 44.0 (C-13), 50.1 (CH), 52.6 (CH), 56.3 (CH), 73.9 (C-3), 122.4 (C-6), 139.8 (C-5), 159.7 (C-3′), 170.6 (3-Ac-C), 172.4 (C-5′), ESI-MS: 413 $[M+H]^+$; Anal. Calcd for $C_{25}H_{36}N_2O_3$ C, 72.78; H, 8.80. Found: C, 72.64; H, 8.67.

3.4. General procedure for the MW-assisted synthesis of 1'-aryl-substituted steroidal 17-exo-pyrazoles **6d**–**j**

Arylhydrazine hydrochloride (**5d**–**j**, 1.0 mmol) and NaOAc (82 mg, 1.0 mmol) were dissolved in EtOH (10 mL). The reaction mixture was stirred at 40 °C for 10 min and then compound **4** (333 mg, 0.80 mmol) dissolved in AcOH (20 mL) was added. The reaction mixture was irradiated at 120 °C for a given time (max. power: 150 W) and then poured into cold water (10 mL). The resulting precipitate was filtered and the crude product purified by column chromatography.

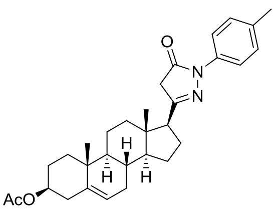

3.4.1. 3β-Acetoxy-17β-[5'-hydroxy-1'-(4''-tolyl)-1'*H*-pyrazol-3'-yl]androst-5-ene (**6d**)

4-Tolylhydrazine hydrochloride (**5d**·HCl, 159 mg) was used for the synthesis as described in the General procedure. Irradiation time: 10 min. Flash chromatography: EtOAc/hexane 20:80. Yield: 336 mg (white solid). Mp: 129–132 °C, R_f = 0.37 (ss C), IR (cm^{-1}): 1729, 1617, 1606, 1558, 1512, 1340, 1241, 1030, 811, 748, ^{1}H NMR ($CDCl_3$, 500 MHz): δ 0.69 (s, 3H, 18-H_3), 1.03 (s, 3H, 19-H_3), 2.03 (s, 3H, Ac-CH_3), 2.34 (s, 3H, 4''-CH_3), 2.45 (t, 1H, J = 9.4 Hz, 17-H), 3.35 (d, 1H, J = 23.0 Hz) and 3.45 (d, 1H, J = 23.0 Hz):

4′-CH_2, 4.61 (m, 1H, 3-H), 5.39 (m, 1H, 6-H), 7.18 (d, 2H, J = 8.4 Hz, 3"-H and 5"-H), 7.75 (d, 2H, J = 8.4 Hz, 2"-H and 6"H), ^{13}C NMR ($CDCl_3$, 125 MHz): δ 13.6 (C-18), 19.5 (C-19), 21.0 (CH_2), 21.1 (4"-CH_3), 21.6 (Ac-CH_3), 23.8 (CH_2), 24.5 (CH_2), 27.9 (CH_2), 31.9 (CH_2), 32.1 (CH), 36.8 (C-10), 37.1 (CH), 38.2 (CH_2), 38.3 (CH_2), 43.2 (CH_2), 44.1 (C-13), 50.1 (CH), 52.7 (CH), 56.4 (CH), 74.0 (C-3), 119.1 (2C, C-2″ and C-6″), 122.4 (C-6), 129.4 (2C, C-3" and C-5"), 134.7 (C-4"), 136.0 (C-1"), 139.9 (C-5), 160.1 (C-3′), 170.7 (2C, Ac-CO and C-5′), ESI-MS: 489 $[M+H]^+$; Anal. Calcd for $C_{31}H_{40}N_2O_3$ C, 76.19; H, 8.25. Found: C, 76.05; H, 8.37.

3.4.2. 3β-Acetoxy-17β-[5'-hydroxy-1'-(4"-isopropylphenyl)-1'*H*-pyrazol-3'-yl]androst-5-ene (**6e**)

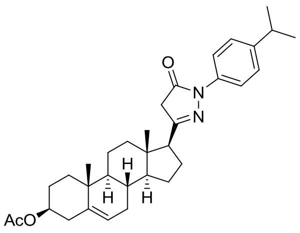

4-Isopropylphenylhydrazine hydrochloride (**5e**·HCl, 187 mg) was used for the synthesis as described in the General procedure. Irradiation time: 10 min. Flash chromatography: EtOAc/CH_2Cl_2 10:90. Yield: 372 mg (white solid). Mp: 123–126 °C, R_f = 0.42 (ss C), IR (cm^{-1}): 2935, 1732, 1556, 1512, 1375, 1363, 1234, 1035, 835, ^{1}H NMR ($CDCl_3$, 500 MHz): δ 0.69 (s, 3H, 18-H_3), 1.03 (s, 3H, 19-H_3), 1.24 (d, 6H, J = 6.8 Hz, 2 × ^{i}Pr-CH_3), 2.03 (s, 3H, Ac-CH_3), 2.45 (t, 1H, J = 9.2 Hz, 17-H), 2.90 (m, 1H, ^{i}Pr-CH), 3.35 (d, 1H, J = 23.0 Hz) and 3.46 (d, 1H, J = 23.0 Hz): 4′-CH_2, 4.61 (m, 1H, 3-H), 5.38 (m, 1H, 6-H), 7.24 (d, 2H, J = 8.4 Hz, 2"-H and 6"-H), 7.77 (d, 2H, J = 8.4 Hz, 3"-H and 5"-H); ^{13}C NMR ($CDCl_3$, 125 MHz): δ 13.6 (C-18), 19.5 (C-19), 21.0 (CH_2), 21.5 (Ac-CH_3), 23.8 (CH_2), 24.1 (2C, 2 × ^{i}Pr-CH_3), 24.5 (CH_2), 27.8 (CH_2), 31.9 (CH_2), 32.1 (CH), 33.8 (^{i}Pr -CH), 36.8 (C-10), 37.1 (CH), 38.2 (CH_2), 38.3 (CH_2), 43.1 (CH_2), 44.1 (C-13), 50.1 (CH), 52.7 (CH), 56.4 (CH), 74.0 (C-3), 119.2 (2C, C-2" and C-6"), 122.4 (C-6), 126.8 (2C, C-3" and C-5"), 136.1 (C-1"), 139.9 (C-5), 145.9 (C-4"), 160.1 (C-3′), 170.7 (2C, Ac-CO and C-5′), ESI-MS: 517 $[M+H]^+$; Anal. Calcd for $C_{33}H_{44}N_2O_3$ C, 76.71; H, 8.58. Found: C, 76.65; H, 8.39.

3.4.3. 3β-Acetoxy-17β-[5'-hydroxy-1'-(4"-*tert*-butylphenyl)-1'*H*-pyrazol-3'-yl]androst-5-ene (**6f**)

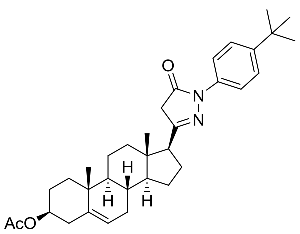

4-*tert*-Butylphenylhydrazine hydrochloride (**5f**·HCl, 201 mg) was used for the synthesis as described in the General

procedure. Irradiation time: 10 min. Flash chromatography: EtOAc/CH_2Cl_2 20:80. Yield: 369 mg (white solid). Mp: 130–133 °C, R_f = 0.28 (ss B), IR (cm^{-1}): 2951, 1732, 1556, 1515, 1376, 1363, 1239, 1034, 835, ^1H NMR ($CDCl_3$, 500 MHz): δ 0.69 (s, 3H, 18-H_3), 1.03 (s, 3H, 19-H_3), 1.31 (s, 9H, 3 × ^{t}Bu-CH_3), 2.03 (s, 3H, Ac-CH_3), 2.45 (t, 1H, J = 9.3 Hz, 17-H), 3.35 (d, 1H, J = 23.0 Hz) and 3.46 (d, 1H, J = 23.0 Hz): 4′-CH_2, 4.61 (m, 1H, 3-H), 5.39 (m, 1H, 6-H), 7.40 (d, 2H, J = 8.6 Hz, 3"-H and 5"-H), 7.78 (d, 2H, J = 8.6 Hz, 2"-H and 6"-H), ^{13}C NMR ($CDCl_3$, 125 MHz): δ 13.6 (C-18), 19.5 (C-19), 21.0 (CH_2), 21.6 (Ac-CH_3), 23.8 (CH_2), 24.5 (CH_2), 27.9 (CH_2), 31.5 (3C, 3 × ^{t}Bu-CH_3), 31.9 (CH_2), 32.2k (CH), 34.4 (^{t}Bu-C), 36.8 (C-10), 37.2 (CH_2), 38.2 (CH_2), 38.24 (CH_2), 43.1 (CH_2), 44.1 (C-13), 50.1 (CH), 52.7 (CH), 56.4 (CH), 74.0 (C-3), 118.9 (2C, C-2" and C-6"), 122.4 (C-6), 125.8 (2C, C-3" and C-5"), 135.8 (C-1"), 139.9 (C-5), 148.1 (C-4"), 160.1 (C-3′), 170.7 (2C, Ac-CO and C-5′), ESI-MS: 531 $[M+H]^+$; Anal. Calcd for $C_{34}H_{46}N_2O_3$ C, 76.94; H, 8.74. Found: C, 77.05; H, 8.59.

3.4.4. 3β-Acetoxy-17β-[5'-hydroxy-1'-(4"-methoxyphenyl)-1'*H*-pyrazol-3'-yl]androst-5-ene (**6g**)

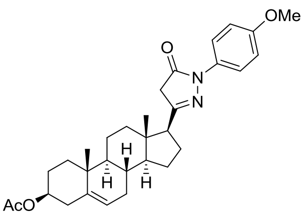

4-Methoxyphenylhydrazine hydrochloride (**5g**·HCl, 175 mg) was used for the synthesis as described in the General procedure. Irradiation time: 5 min. Flash chromatography: EtOAc/CH_2Cl_2 30:70. Yield: 371 mg (white solid). Mp: 139–142 °C, R_f = 0.22 (ss C), IR (cm^{-1}): 2933, 1730, 1699, 1508, 1245, 1035, 828, ^1H NMR ($CDCl_3$, 500 MHz): δ 0.69 (s, 3H, 18-H_3), 1.03 (s, 3H, 19-H_3), 2.03 (s, 3H, Ac-CH_3), 2.44 (t, 1H, J = 9.3 Hz, 17-H), 3.34 (d, 1H, J = 23.0 Hz) and 3.45 (d, 1H, J = 23.0 Hz): 4′-CH_2, 3.81 (s, 3H, 4"-OMe), 4.61 (m, 1H, 3-H), 5.38 (m, 1H, 6-H), 6.91 (d, 2H, J = 9.0 Hz, 3"-H and 5"-H), 7.76 (d, 2H, J = 9.0 Hz, 2"-H and 6"-H), ^{13}C NMR ($CDCl_3$, 125 MHz): δ 13.6 (C-18), 19.5 (C-19), 21.0 (CH_2), 21.5 (Ac-CH_3), 23.8 (CH_2), 24.5 (CH_2), 27.9 (CH_2), 31.9 (CH_2), 32.1 (CH), 36.8 (C-10), 37.1 (CH_2), 38.2 (CH_2), 38.3 (CH_2), 43.1 (CH_2), 44.1 (C-13), 50.1 (CH), 52.7 (CH), 55.6 (4"-OMe), 56.4 (CH), 74.0 (C-3), 114.1 (2C, C-3" and C-5"), 120.8 (2C, C-2" and C-6"), 122.4 (C-6), 131.8 (C-1"), 139.9 (C-5), 157.1 (C-4"), 160.1 (C-3′), 170.5 and 170.7 (Ac-CO and C-5′), ESI-MS: 505 $[M+H]^+$; Anal. Calcd for $C_{31}H_{40}N_2O_4$ C, 73.78; H, 7.99. Found: C, 73.67; H, 8.09.

3.4.5. 3β-Acetoxy-17β-[5'-hydroxy-1'-(4''-fluorophenyl)-1'*H*-pyrazol-3'-yl]androst-5-ene (**6h**)

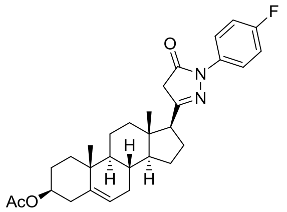

4-Fluorophenylhydrazine hydrochloride (**5h**·HCl, 163 mg) was used for the synthesis as described in the General procedure. Irradiation time: 30 min. Flash chromatography: EtOAc/hexane 20:80. Yield: 335 mg (white solid). Mp: 162–165 °C, R_f = 0.36 (ss C), IR (cm^{-1}): 2949, 1732, 1718, 1508, 1239, 1219, 1024, 835, ^1H NMR ($CDCl_3$, 500 MHz): δ 0.69 (s, 3H, 18-H_3), 1.03 (s, 3H, 19-H_3), 2.03 (s, 3H, Ac-CH_3), 2.45 (t, 1H, J = 9.3 Hz, 17-H), 3.37 (d, 1H, J = 23.1 Hz) and 3.47 (d, 1H, J = 23.1 Hz): 4′-CH_2, 4.61 (m, 1H, 3-H), 5.39 (m, 1H, 6-H), 7.07 (m, 2H, 3''-H and 5''-H), 7.86 (m, 2H, 2''-H and 6''-H), ^{13}C NMR ($CDCl_3$, 125 MHz): δ 13.6 (C-18), 19.5 (C-19), 21.0 (CH_2), 21.6 (Ac-CH_3), 23.8 (CH_2), 24.4 (CH_2), 27.8 (CH_2), 31.8 (CH_2), 32.1 (CH), 36.8 (C-10), 37.1 (CH_2), 38.2 (CH_2), 38.3 (CH_2), 43.1 (CH_2), 44.1 (C-13), 50.1 (CH), 52.7 (CH), 56.4 (CH), 73.9 (C-3), 115.6 (d, 2C, J = 22.6 Hz, C-3'' and C-5''), 120.7 (d, 2C, J = 7.9 Hz, C-3'' and C-5''), 122.4 (C-6), 134.5 (C-1''), 139.9 (C-5), 159.9 (d, J = 243.9 Hz, C-4''), 160.4 (C-3′), 170.7 (2C, Ac-CO and C-5′), ESI-MS: 493 $[M+H]^+$; Anal. Calcd for $C_{30}H_{37}FN_2O_3$ C, 73.14; H, 7.57. Found: C, 73.27; H, 7.42.

3.4.6. 3β-Acetoxy-17β-[5'-hydroxy-1'-(4''-chlorophenyl)-1'*H*-pyrazol-3'-yl]androst-5-ene (**6i**)

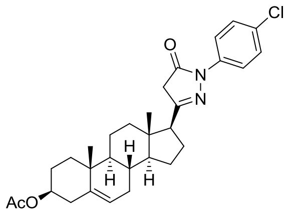

4-Chlorophenylhydrazine hydrochloride (**5i**·HCl, 179 mg) was used for the synthesis as described in the General procedure. Irradiation time: 30 min. Flash chromatography: EtOAc/hexane 20:80. Yield: 338 mg (white solid). Mp: 180–183 °C, R_f = 0.40 (ss C), IR (cm^{-1}): 2939, 1723, 1493, 1345, 1334, 1234, 1026, 828, ^1H NMR ($CDCl_3$, 500 MHz): δ 0.69 (s, 3H, 18-H_3), 1.03 (s, 3H, 19-H_3), 2.03 (s, 3H, Ac-CH_3), 2.45 (t, 1H, J = 9.2 Hz, 17-H), 3.37 (d, 1H, J = 23.1 Hz) and 3.47 (d, 1H, J = 23.1 Hz): 4′-CH_2, 4.61 (m, 1H, 3-H), 5.38 (m, 1H, 6-H), 7.33 (d, 2H, J = 8.8 Hz, 3''-H and 5''-H), 7.87 (d, 2H, J = 8.8 Hz, 2''-H and 6''-H), ^{13}C NMR ($CDCl_3$, 125 MHz): δ 13.6 (C-18), 19.5 (C-19), 21.0 (CH_2), 21.6 (Ac-CH_3), 23.8 (CH_2), 24.4 (CH_2), 27.9 (CH_2), 31.8 (CH_2), 32.1 (CH), 36.8 (C-10), 37.1 (CH_2), 38.2 (CH_2), 38.4 (CH_2), 43.2 (CH_2), 44.1 (C-13), 50.1 (CH), 52.7 (CH), 56.4 (CH), 73.9 (C-3), 120.0 (2C, C-2'' and C-6''), 122.4 (C-6), 128.9 (2C, C-3'' and C-5''), 130.0 (C-4''),

137.0 (C-1''), 139.9 (C-5), 160.6 (C-3′), 170.7 (2C, Ac-CO and C-5′), ESI-MS: 509 $[M+H]^+$; Anal. Calcd for $C_{30}H_{37}ClN_2O_3$ C, 70.78; H, 7.33. Found: C, 70.87; H, 7.19.

3.4.7 3β-Acetoxy-17β-[5'-hydroxy-1'-(4''-bromophenyl)-1'*H*-pyrazol-3'-yl]androst-5-ene (**6j**)

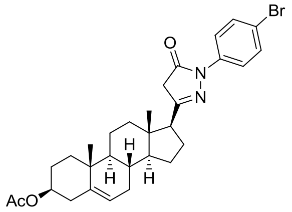

4-Bromophenylhydrazine hydrochloride (**5j**·HCl, 224 mg) was used for the synthesis as described in the General procedure. Irradiation time: 30 min. Flash chromatography: EtOAc/hexane 20:80. Yield: 376 mg (white solid). Mp: 190–193 °C, R_f = 0.42 (ss C), IR (cm^{-1}): 2940, 1723, 1488, 1236, 1024, 825, ^{1}H NMR ($CDCl_3$, 500 MHz): δ 0.69 (s, 3H, 18-H_3), 1.03 (s, 3H, 19-H_3), 2.03 (s, 3H, Ac-CH_3), 2.45 (t, 1H, J = 9.3 Hz, 17-H), 3.37 (d, 1H, J = 23.1 Hz) and 3.47 (d, 1H, J = 23.1 Hz): 4′-CH_2, 4.61 (m, 1H, 3-H), 5.38 (m, 1H, 6-H), 7.48 (d, 2H, J = 8.8 Hz, 3''-H and 5''-H), 7.82 (d, 2H, J = 8.8 Hz, 2''-H and 6''-H), ^{13}C NMR ($CDCl_3$, 125 MHz): δ 13.6 (C-18), 19.5 (C-19), 21.0 (CH_2), 21.6 (Ac-CH_3), 23.8 (CH_2), 24.5 (CH_2), 27.9 (CH_2), 31.8 (CH_2), 32.1 (CH), 36.8 (C-10), 37.1 (CH_2), 38.2 (CH_2), 38.4 (CH_2), 43.2 (CH_2), 44.1 (C-13), 50.1 (CH), 52.7 (CH), 56.4 (CH), 73.9 (C-3), 117.8 (C-4''), 120.3 (2C, C-2'' and C-6''), 122.4 (C-6), 131.9 (2C, C-3'' and C-5''), 137.5 (C-1''), 139.9 (C-5), 160.6 (C-3′), 170.7 (2C, Ac-CO and C-5′), ESI-MS: 553 $[M+H]^+$; Anal. Calcd for $C_{30}H_{37}BrN_2O_3$ C, 65.10; H, 6.74. Found: C, 64.97; H, 6.82.

4. General procedure for the synthesis of compounds 7a–j and characterization data

<u>General procedure</u>: Compound **6a**–**j** (0.50 mmol) was dissolved in MeOH (10 mL), and KOH (50 mg, 0.89 mmol) was added. The solution was stirred at room temperature for 4 h, then diluted with water and neutralized with diluted HCl. The resulting precipitate was filtered, washed with water and dried.

4.1. 3β-Hydroxy-17β-[5'-hydroxy-1'*H*-pyrazol-3'-yl]androst-5-ene (**7a**)

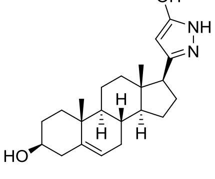

Compound **6a** (199 mg) was used for the synthesis as described in the General procedure. Yield: 162 mg (91%, white solid). Mp: 248–251 °C, R_f = 0.30 (ss F), IR (cm^{-1}): 3433, 3146, 2942, 1618, 1516, 1050, ^{1}H NMR (DMSO-d_6, 500 MHz): δ 0.48 (s, 3H, 18-

H_3), 0.94 (s, 3H, 19-H_3), 2.48 (t, 1H, J = 9.6 Hz, 17-H), 3.26 (m, 1H, 3-H), 4.60 (bs, 1H, 3-OH), 5.23 (s, 4′-H), 5.28 (m, 1H, 6-H), 10.78 (bs, NH/OH), ^{13}C NMR (DMSO-d_6, 125 MHz): δ 12.9 (C-18), 19.2 (C-19), 20.4 (CH_2), 24.0 (CH_2), 25.8 (CH_2), 31.3 (CH_2), 31.4 (CH_2), 31.9 (CH), 36.2 (CH_2), 37.0 (CH_2), 37.1 (C-10), 42.2 (CH_2), 43.1 (CH_2), 48.2 (C-13), 49.9 (C-17), 55.3 (CH), 70.0 (C-3), 88.4 (C-4′), 120.3 (C-6), 141.3 (C-5), 144.1 and 160.7 (C-3′ and C-5′), ESI-MS: 357 $[M+H]^+$; Anal. Calcd for $C_{22}H_{32}N_2O_2$ C, 74.12; H, 9.05. Found: C, 74.25; H, 9.15.

4.2. 3β-Hydroxy-17β-[5'-hydroxy-1'-phenyl-1'*H*-pyrazol-3'-yl]androst-5-ene (**7b**)

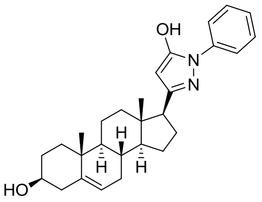

Compound **6b** (237 mg) was used for the synthesis as described in the General procedure. Yield: 190 mg (88%, white solid). Mp: 198–200 °C, R_f = 0.27 (ss D), IR (cm^{-1}): 3124, 2925, 1712, 1620, 1096, 750, 688, ^{1}H NMR (DMSO-d_6, 500 MHz): δ 0.54 (s, 3H, 18-H_3), 0.94 (s, 3H, 19-H_3), 3.27 (m, 1H, 3-H), 4.59 (bs, 1H, 3-OH), 5.29 (m, 1H, 6-H), 5.35 (s, 4′-H), 7.19 (m, 1H, 4"-H), 7.41 (m, 2H, 3"-H and 5"-H), 7.72 (d, 2H, J = 8.5 Hz, 2"-H and 6"-H), 11.36 (bs, NH/OH), ^{13}C NMR (DMSO-d_6, 125 MHz): δ 13.0 (C-18), 19.2 (C-19), 20.4 (CH_2), 24.1 (CH_2), 25.1 (CH_2), 31.4 (2C, 2 × CH_2), 31.8 (CH), 36.2 (CH_2), 37.0 (CH_2), 37.5 (C-10), 42.2 (CH_2), 43.0 (C-13), 49.9 (CH), 50.3 (C-17), 55.6 (CH), 70.0 (C-3), 87.0 (C-4′), 120.3 (C-6), 120.5 (2C, C-2" and C-6"), 124.8 (C-4"), 128.7 (2C, C-3" and C-5"), 139.1 (C-1"), 141.3 (C-5), 152.1 and 152.6 (C-3′ and C-5′), ESI-MS: 433 $[M+H]^+$; Anal. Calcd for $C_{28}H_{36}N_2O_2$ C, 77.74; H, 8.39. Found: C, 77.85; H, 8.26.

4.3. 3β-Hydroxy-17β-[5'-hydroxy-1'-methyl-1'*H*-pyrazol-3'-yl]androst-5-ene (**7c**)

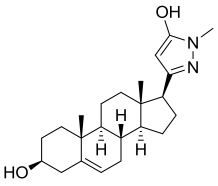

Compound **6c** (206 mg) was used for the synthesis as described in the General procedure. Yield: 167 mg (90%, white solid). Mp: 228–230 °C, R_f = 0.36 (ss E), IR (cm^{-1}): 3242, 2931, 1590, 1443, 1302, 1045, ^{1}H NMR (DMSO-d_6, 500 MHz): δ 0.47 (s, 3H, 18-H_3), 0.93 (s, 3H, 19-H_3), 3.27 (m, 1H, 3-H), 3.34 (s, 3H, 1'-CH_3), 4.60 (bs, 1H, 3-OH), 5.10 (s, 4′-H), 5.28 (m, 1H, 6-H), 10.60 (bs, NH/OH), ESI-MS: 471 $[M+H]^+$; Anal. Calcd for $C_{23}H_{34}N_2O_2$ C, 74.55; H, 9.25. Found: C, 74.39; H, 9.12.

4.4. 3β-Hydroxy-17β-[5'-hydroxy-1'-(4''-tolyl)-1'*H*-pyrazol-3'-yl]androst-5-ene (**7d**)

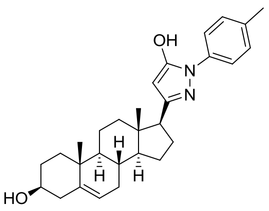

Compound **6d** (244 mg) was used for the synthesis as described in the General procedure. Yield: 205 mg (92%, white solid). Mp: 130–132 °C, R_f = 0.28 (ss D), IR (cm^{-1}): 3385, 2927, 1712, 1688, 1595, 1510, 1345, 1043, 815, ^{1}H NMR (DMSO-d_6, 500 MHz): δ 0.53 (s, 3H, 18-H_3), 0.94 (s, 3H, 19-H_3), 2.30 (s, 3H, 4''-CH_3), 3.26 (m, 1H, 3-H), 4.61 (bs, 1H, 3-OH), 5.28 (m, 1H, 6-H), 5.33 (s, 4′-H), 7.21 (d, 2H, *J* = 7.8 Hz, 2''-H and 6''-H), 7.58 (d, 2H, *J* = 7.8 Hz, 3''-H and 5''-H), 11.27 (bs, NH/OH), ^{13}C NMR (DMSO-d_6, 125 MHz): δ 13.0 (C-18), 19.2 (C-19), 20.4 (CH_2), 20.5 (4''-CH_3), 24.2 (CH_2), 25.2 (CH_2), 31.5 (2C, 2 × CH_2), 31.9 (CH), 36.2 (CH_2), 37.0 (CH_2), 37.5 (C-10), 42.3 (CH_2), 43.1 (C-13), 49.9 (CH), 50.4 (C-17), 55.6 (CH), 70.0 (C-3), 86.8 (C-4′), 120.4 (C-6), 120.6 (2C, C-2'' and C-6''), 129.1 (2C, C-3'' and C-5''), 134.0 (C-4''), 136.8 (C-1''),141.3 (C-5), 151.8 and 152.4 (C-3′ and C-5′), ESI-MS: 447 [M+H]$^+$; Anal. Calcd for $C_{29}H_{38}N_2O_2$ C, 77.99; H, 8.58. Found: C, 78.09; H, 8.42.

4.5. 3β-Hydroxy-17β-[5'-hydroxy-1'-(4''-isopropylphenyl)-1'*H*-pyrazol-3'-yl]androst-5-ene (**7e**)

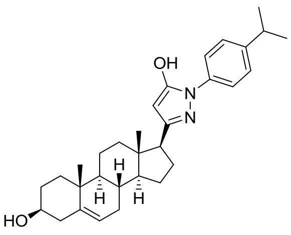

Compound **6e** (258 mg) was used for the synthesis as described in the General procedure. Yield: 209 mg (88%, white solid). Mp: 128–131 °C, R_f = 0.32 (ss D), IR (cm^{-1}): 3359, 2929, 1710, 1599, 1558, 1510, 1351, 1053, 833, ^{1}H NMR (DMSO-d_6, 500 MHz): δ 0.52 (s, 3H, 18-H_3), 0.94 (s, 3H, 19-H_3), 1.20 (d, 6H, *J* = 6.7 Hz, 2 × ^{i}Pr-CH_3), 2.88 (m, 1H, ^{i}Pr-CH), 3.26 (m, 1H, 3-H), 4.59 (bs, 1H, 3-OH), 5.28 (m, 1H, 6-H), 5.32 (s, 4′-H), 7.27 (d, 2H, *J* = 8.3 Hz, 3''-H and 5''-H), 7.59 (d, 2H, *J* = 8.3 Hz, 2''-H and 6''-H), 11.22 (bs, NH/OH), ^{13}C NMR (DMSO-d_6, 125 MHz): δ 13.0 (C-18), 19.2 (C-19), 20.4 (CH_2), 23.9 (2C, 2 × ^{i}Pr-CH_3), 24.2 (CH_2), 25.1 (CH_2), 31.4 (2C, 2 × CH_2), 31.9 (CH), 32.9 (^{i}Pr -CH), 36.2 (CH_2), 37.0 (CH_2), 37.5 (C-10), 42.2 (CH_2), 43.0 (C-13), 49.9 (CH), 50.3 (C-17), 55.6 (CH), 70.0 (C-3), 86.8 (C-4′), 120.4 (C-6), 120.8 (2C, C-2'' and C-6''), 126.5 (2C, C-3'' and C-5''), 137.0 (C-4″), 141.3 (C-5), 145.1 (C-1''), 151.8 és 152.3 (C-3′ and C-5), ESI-MS: 475 [M+H]$^+$; Anal. Calcd for $C_{31}H_{42}N_2O_2$ C, 78.44; H, 8.92. Found: C, 78.56; H, 8.79.

4.6. 3β-Hydroxy-17β-[5'-hydroxy-1'-(4''-*tert*-butylphenyl)-1'*H*-pyrazol-3'-yl]androst-5-ene (**7f**)

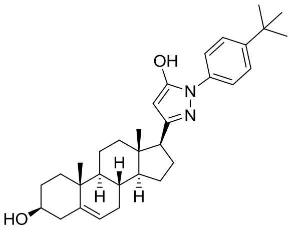

Compound **6f** (265 mg) was used for the synthesis as described in the General procedure. Yield: 213 mg (87%, white solid). Mp: 152–154 °C, R_f = 0.34 (ss D), IR (cm^{-1}): 3368, 2931, 1710, 1602, 1558, 1512, 1460, 1362, 1048, 835, ^{1}H-NMR ($CDCl_3$, 500 MHz): 0.69 (s, 3H, 18-H_3), 1.02 (s, 3H, 19-H_3), 1.32 (s, 9H, 3 × ^{t}Bu-CH_3), 2.45 (t, 1H, *J* = 9.3 Hz, 17-H), 3.36 (d, 1H, *J* = 23.0 Hz) and 3.46 (d, 1H, *J* = 23.0 Hz): 4′-CH_2, 3.52 (m, 1H, 3-H), 5.37 (m, 1H, 6-H), 7.40 (d, 2H, *J* = 8.6 Hz, 3''-H and 5''-H), 7.78 (d, 2H, *J* = 8.6 Hz, 2''-H and 6''-H), ^{13}C NMR ($CDCl_3$, 125 MHz): δ 13.6 (C-18), 19.6 (C-19), 21.1 (CH_2), 23.8 (CH_2), 24.5 (CH_2), 31.5 (3C, 3 × ^{t}Bu-CH_3), 31.7 (CH_2), 31.9 (CH_2), 32.2 (CH), 34.6 (^{t}Bu-C), 36.7 (C-10), 37.4 (CH_2), 38.4 (CH_2), 42.4 (CH_2), 43.1 (CH_2), 44.1 (C-13), 50.2 (CH), 52.7 (CH), 56.5 (CH), 71.8 (C-3), 118.9 (2C, C-2'' and C-6''), 121.4 (C-6), 125.8 (2C, C-3'' and C-5''), 135.8 (C-1''), 141.0 (C-5), 148.1 (C-4''), 160.1 (C-3′), 170.7 (C-5′),

^{1}H NMR (DMSO-d_6, 500 MHz): δ 0.52 (s, 3H, 18-H_3), 0.94 (s, 3H, 19-H_3), 1.28 (s, 9H, 3 × ^{t}Bu-CH_3), 3.26 (m, 1H, 3-H), 4.60 (bs, 1H, 3-OH), 5.28 (m, 1H, 6-H), 5.32 (s, 4′-H), 7.42 (d, 2H, *J* = 8.2 Hz, 3''-H and 5''-H), 7.60 (d, 2H, *J* = 8.2 Hz, 2''-H and 6''-H), 11.24 (bs, NH/OH), ^{13}C NMR (DMSO-d_6, 125 MHz): δ 13.0 (C-18), 19.2 (C-19), 20.4 (CH_2), 24.2 (CH_2), 25.1 (CH_2), 31.2 (3C, 3 × ^{t}Bu-CH_3), 31.5 (2C, 2 × CH_2), 31.9 (CH), 34.1 (^{t}Bu-C), 36.2 (CH_2), 37.0 (CH_2), 37.5 (C-10), 42.3 (CH_2), 43.1 (C-13), 49.9 (CH), 50.4 (C-17), 55.6 (CH), 70.0 (C-3), 86.8 (C-4′), 120.4 (3C, C-2'', C-6'' and C-6), 125.4 (2C, C-3'' and C-5''), 136.7 (C-1''), 141.3 (C-5), 147.3 (C-4''), 151.8 and 152.4 (C-3′ and C-5′), ESI-MS: 489 $[M+H]^+$; Anal. Calcd for $C_{32}H_{44}N_2O_2$ C, 78.65; H, 9.07. Found: C, 78.76; H, 8.95.

4.7. 3β-Hydroxy-17β-[5'-hydroxy-1'-(4''-methoxyphenyl)-1'*H*-pyrazol-3'-yl]androst-5-ene (**7g**)

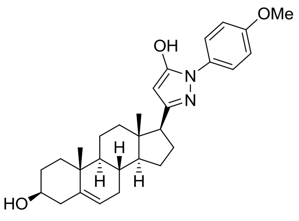

Compound **6g** (252 mg) was used for the synthesis as described in the General procedure. Yield: 213 mg (92%, white solid). Mp: 179–182 °C, R_f = 0.36 (ss D), IR (cm^{-1}): 3533, 2933, 1704, 1510, 1241, 1026, 831, ^{1}H NMR (DMSO-

d_6, 500 MHz): δ 0.53 (s, 3H, 18-H_3), 0.94 (s, 3H, 19-H_3), 3.26 (m, 1H, 3-H), 3.76 (s, 3H, 4''-OMe), 4.61 (bs, 1H, 3-OH), 5.28 (m, 1H, 6-H), 5.31 (s, 4′-H), 6.97 (d, 2H, J = 8.8 Hz, 2''-H and 6''-H), 7.56 (d, 2H, J = 8.8 Hz, 3''-H and 5''-H), 11.18 (bs, NH/OH), ^{13}C NMR (DMSO-d_6, 125 MHz): δ 13.0 (C-18), 19.2 (C-19), 20.5 (CH_2), 24.2 (CH_2), 25.2 (CH_2), 31.5 (2C, 2 × CH_2), 31.9 (CH), 36.2 (CH_2), 37.0 (CH_2), 37.5 (C-10), 42.3 (CH_2), 43.1 (C-13), 49.9 (CH), 55.3 (2C, C-17 and 4''-OMe), 55.6 (CH), 70.0 (C-3), 86.5 (C-4′), 113.9 (2C, C-3'' and C-5''), 120.4 (C-6), 122.4 (2C, C-2'' and C-6''), 132.5 (C-1''), 141.4 (C-5), 151.5 and 152.1 (C-3′ and C-5)′, 156.67 (C-4''), ESI-MS: 463 $[M+H]^+$; Anal. Calcd for $C_{29}H_{38}N_2O_3$ C, 75.29; H, 8.28. Found: C, 75.07; H, 8.14.

4.8. 3β-Hydroxy-17β-[5'-hydroxy-1'-(4''-fluorophenyl)-1'*H*-pyrazol-3'-yl]androst-5-ene (**7h**)

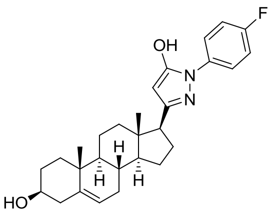

Compound **6g** (246 mg) was used for the synthesis as described in the General procedure. Yield: 205 mg (91%, white solid). Mp: 126–130 °C, R_f = 0.27 (ss D), IR (cm^{-1}): 3376, 2931, 1710, 1506, 1219, 1045, 832, ^{1}H NMR (DMSO-d_6, 500 MHz): δ 0.53 (s, 3H, 18-H_3), 0.94 (s, 3H, 19-H_3), 3.27 (m, 1H, 3-H), 4.59 (bs, 1H, 3-OH), 5.28 (m, 1H, 6-H), 5.34 (s, 4′-H), 7.25 (m, 2H, 3''-H and 5''-H), 7.72 (m, 2H, 2''-H and 6''-H), 11.43 (bs, NH/OH), ^{13}C NMR (DMSO-d_6, 125 MHz): δ 13.0 (C-18), 19.2 (C-19), 20.4 (CH_2), 24.1 (CH_2), 25.1 (CH_2), 31.4 (2C, 2 × CH_2), 31.8 (CH), 36.2 (CH_2), 37.0 (CH_2), 37.5 (C-10), 42.2 (CH_2), 43.1 (C-13), 49.9 (CH), 50.3 (C-17), 55.6 (CH), 70.0 (C-3), 86.9 (C-4′), 115.4 (d, 2C, J = 22.6 Hz, C-3'' and C-5''), 120.3 (C-6), 122.4 (2C, C-2'' and C-6''), 135.6 (C-1''), 141.3 (C-5), 152.2 and 152.4 (C-3′ and C-5′). 159.3 (d, J = 242.4 Hz, C-4″), ESI-MS: 451 $[M+H]^+$; Anal. Calcd for $C_{28}H_{35}FN_2O_2$ C, 74.64; H, 7.83. Found: C, 74.76; H, 7.96.

4.9. 3β-Hydroxy-17β-[5'-hydroxy-1'-(4''-chlorophenyl)-1'*H*-pyrazol-3'-yl]androst-5-ene (**7i**)

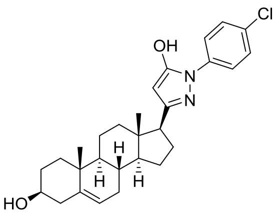

Compound **6i** (255 mg) was used for the synthesis as described in the General procedure. Yield: 198 mg (85%, white solid). Mp: 196–198 °C, R_f = 0.30 (ss D), IR (cm^{-1}): 3457,2931, 1723, 1597, 1490, 1339, 1330, 1048, 828, ^{1}H NMR (DMSO-d_6, 500 MHz): δ 0.53 (s, 3H, 18-H_3), 0.94 (s, 3H, 19-H_3), 3.26 (m, 1H, 3-H), 4.59 (bs, 1H,

3-OH), 5.28 (m, 1H, 6-H), 5.35 (s, 4′-H), 7.47 (d, 2H, J = 8.3 Hz, 3"-H and 5"-H), 7.76 (d, 2H, J = 8.3 Hz, 2"-H and 6"-H), 11.58 (bs, NH/OH), ^{13}C NMR (DMSO-d_6, 125 MHz): δ 13.0 (C-18), 19.2 (C-19), 20.4 (CH_2), 24.1 (CH_2), 25.0 (CH_2), 31.4 (2C, 2 × CH_2), 31.8 (CH), 36.2 (CH_2), 37.0 (CH_2), 37.5 (C-10), 42.2 (CH_2), 43.1 (C-13), 49.9 (CH), 50.3 (C-17), 55.6 (CH), 70.0 (C-3), 87.2 (C-4′), 120.3 (C-6), 121.6 (2C, C-2" and C-6"), 128.7 (2C, C-3" és C-5"), 131.7 (C-4"), 138.0 (C-1"), 141.3 (C-5), 152.6 and 152.8 (C-3′ and C-5′), ESI-MS: 467 $[M+H]^+$; Anal. Calcd for $C_{28}H_{35}ClN_2O_2$ C, 72.01; H, 7.55. Found: C, 71.89; H, 7.62.

4.10. 3β-Hydroxy-17β-[5'-hydroxy-1'-(4"-bromophenyl)-1'*H*-pyrazol-3'-yl]androst-5-ene (**7j**)

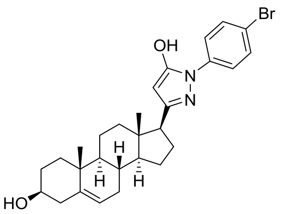

Compound **6j** (277 mg) was used for the synthesis as described in the General procedure. Yield: 230 mg (90%, white solid). Mp: 200–203 °C, R_f = 0.30 (ss D), IR (cm^{-1}): 3426, 2929, 1721, 1488, 1340, 1045, 823, ^{1}H NMR (DMSO-d_6, 500 MHz): δ 0.53 (s, 3H, 18-H_3), 0.94 (s, 3H, 19-H_3), 3.26 (m, 1H, 3-H), 4.59 (bs, 1H, 3-OH), 5.28 (m, 1H, 6-H), 5.35 (s, 4′-H), 7.59 (d, 2H, J = 8.4 Hz, 3"-H and 5"-H), 7.71 (d, 2H, J = 8.4 Hz, 2"-H and 6"-H), 11.58 (bs, NH/OH); ^{13}C NMR (DMSO-d_6, 125 MHz): δ 13.0 (C-18), 19.2 (C-19), 20.4 (CH_2), 24.1 (CH_2), 25.0 (CH_2), 31.4 (2C, 2 × CH_2), 31.8 (CH), 36.2 (CH_2), 37.0 (CH_2), 37.5 (C-10), 42.2 (CH_2), 43.1 (C-13), 49.9 (CH), 50.3 (C-17), 55.6 (CH), 70.0 (C-3), 87.2 (C-4′), 117.0 (C-4"), 120.3 (C-6), 122.0 (2C, C-2" and C-6"), 131.6 (2C, C-3" and C-5"), 138.4 (C-1"), 141.3 (C-5), 152.7 and 152.8 (C-3′ and C-5′), ESI-MS: 511 $[M+H]^+$; Anal. Calcd for $C_{28}H_{35}BrN_2O_2$ C, 65.75; H, 6.90. Found: C, 65.88; H, 6.74.

5. Synthesis and characterization of compound 8

Compound **6a** (100 mg, 0.25 mmol) was added to a mixture of pyridine (5 mL) and acetic anhydride (5 mL) and the solution was kept at room temperature for 6 h. After completion, the mixture was poured into acidic ice and the resulting precipitate was filtered, washed with water and dried. The crude product was purified by column chromatography with EtOAc/CH_2Cl_2 2:98 to give **8** (104 mg, 86%) as white solid.

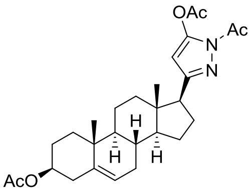

3β-Acetoxy-17β-[1′-acetyl-5′-acetoxy-1′*H*-pyrazol-3′-yl]androst-5-ene (**8**)

Mp: 158–160 °C, R_f = 0.54 (ss A), IR (cm^{-1}): 2940, 1794, 1725, 1597, 1370, 1248, 1177, 1023, ^{1}H NMR ($CDCl_3$, 500 MHz): δ 0.56 (s, 3H, 18-H_3), 1.02 (s, 3H, 19-H_3), 2.03 (s, 3H, 3-Ac-CH_3), 2.31 (s, 3H, 5′-Ac-CH_3), 2.61 (s, 3H, 1′-Ac-CH_3), 4.61 (m, 1H, 3-H), 5.39 (m, 1H, 6-H), 5.93 (s, 1H, 4′-H); ^{13}C NMR ($CDCl_3$, 125 MHz): δ 13.3 (C-18), 19.5 (C-19), 20.7 (Ac-CH_3), 20.9 (CH_2), 21.6 (3-Ac-CH_3), 23.4 (Ac-CH_3), 24.7 (CH_2), 25.2 (CH_2), 27.9 (CH_2), 32.0 (CH_2), 32.3 (CH), 36.8 (C-10), 37.2 (CH_2), 37.9 (CH_2), 38.2 (CH_2), 44.1 (C-13), 50.3 (CH), 50.9 (CH), 56.3 (CH), 74.0 (C-3), 99.9 (C-4′), 122.5 (C-6), 139.9 (C-5), 146.7 and 155.8 (C-3′ and C-5′), 167.7 (Ac-C), 170.2 (Ac-C), 170.7 (3-Ac-C), ESI-MS: 483 $[M+H]^+$; Anal. Calcd for $C_{28}H_{38}N_2O_5$ C, 69.68; H, 7.94. Found: C, 69.53; H, 8.06.

6. Pharmacological methods

In a similar manner as described before [Szabó, J. et al. *Molecules*, **2016**, *21*, 611], the antiproliferative properties of the prepared compounds were determined on a panel of human breast cancer cell lines. Briefly, MCF-7, MDA-MB-231 and T47D breast cancer cell lines and NIH-3T3 mouse fibroblasts were purchased from the European Collection of Cell Cultures (ECCAC, Salisbury, UK) and maintained in minimal essential medium (MEM), supplemented with 10% fetal bovine serum (FBS), 1% non-essential amino acids, and an antibiotic/antimycotic mixture (Lonza Group Ltd., Basel, Switzerland). Near-confluent cancer cells were seeded onto a 96-well microplate at a density of 5000/well and, after an overnight standing, a new medium, containing the test compounds at 10 and 30 µM, was added. After incubation for 72 h at 37 °C in humidified air containing 5% CO_2, the viability of the cells was determined by the addition of 20 µL of 5 mg/mL 3-(4,5-dimethylthiazol-2-yl)-2,5-diphenyltetrazolium bromide (MTT) solution. The mitochondrial reductase of the living cells metabolizes the MTT, and the produced formazan precipitated as purple crystals during a 4-h contact period. The medium was next removed and the formazan dissolved in 100 µL of DMSO during a 60-min period of shaking at 37 °C. Finally, the reduced MTT was assayed at 545 nm, using a microplate reader and wells with untreated cells served as control. In the case of the most active

compounds (i.e., higher than 50% growth inhibition at 10 μM and 80% growth inhibition at 30 μM), the assays were repeated with a set of dilutions (0.1–30 mM), sigmoidal concentration–response curves were fitted to the determined data, and the IC_{50} values were calculated by means of GraphPad Prism 4.0 (GraphPad Software, San Diego, CA, USA). The antiproliferative actions of these active molecules were additionally determined against NIH-3T3 mouse fibroblasts in order to obtain preliminary data concerning the cancer selectivity. All in vitro experiments were carried out on two microplates with five parallel wells. Stock solutions of the tested compounds (10 mM) were prepared in DMSO. The highest DMSO content of the medium (0.3%) did not have any substantial effect on the cell proliferation. Cisplatin (Ebewe Pharma GmbH, Unterach, Austria) was used as a reference agent.

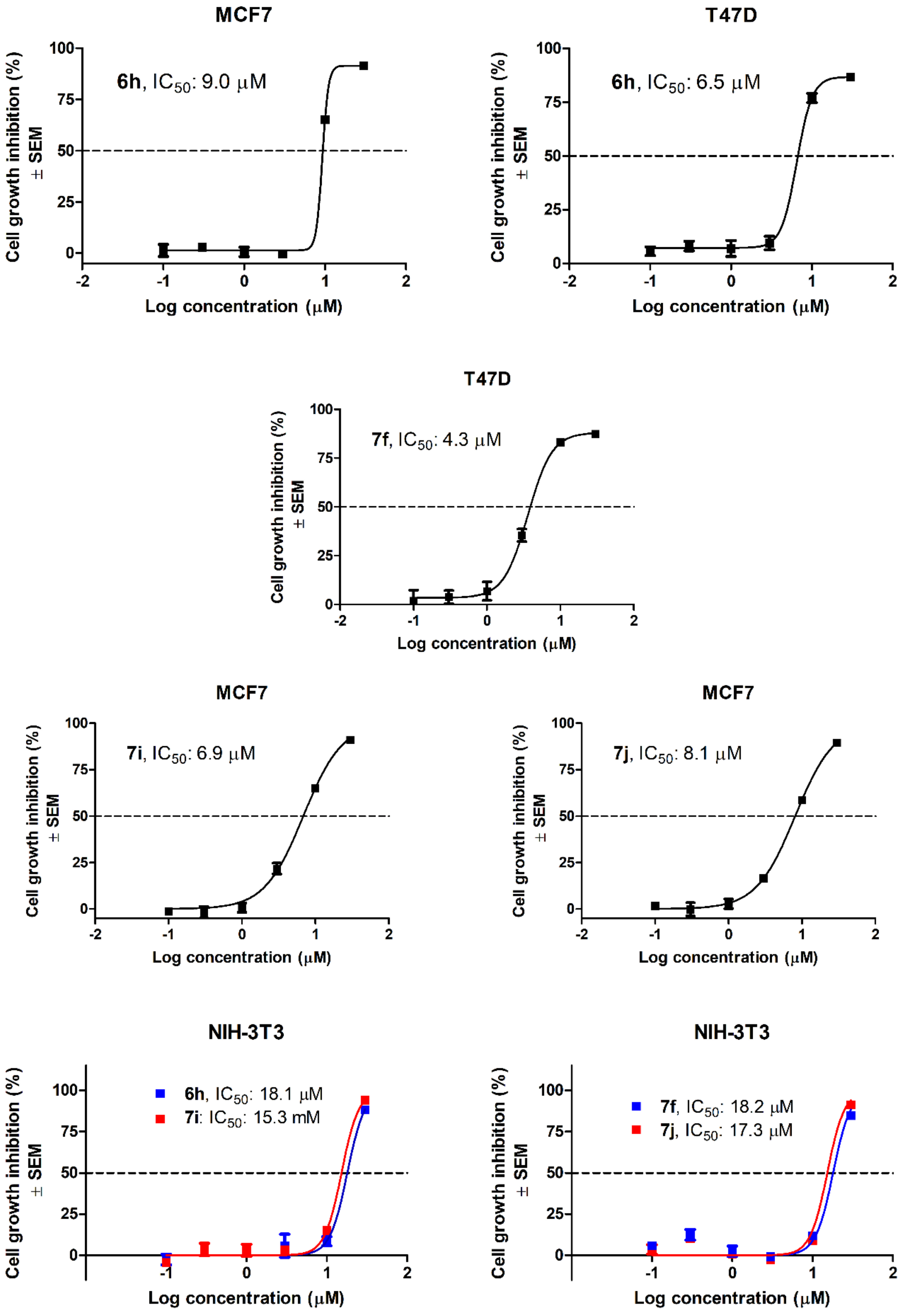

Figure S1: Concentration–response curves of the most effective molecules

7. NMR spectra collection

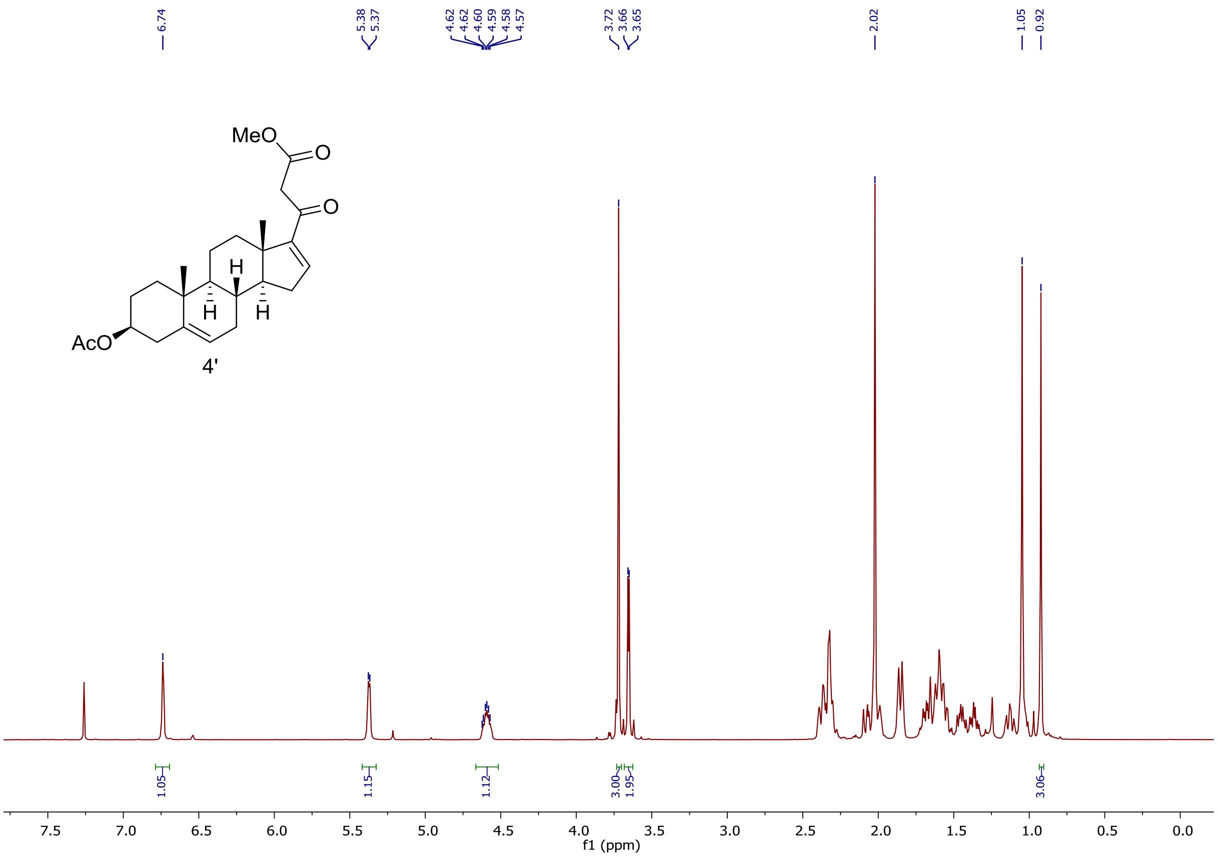

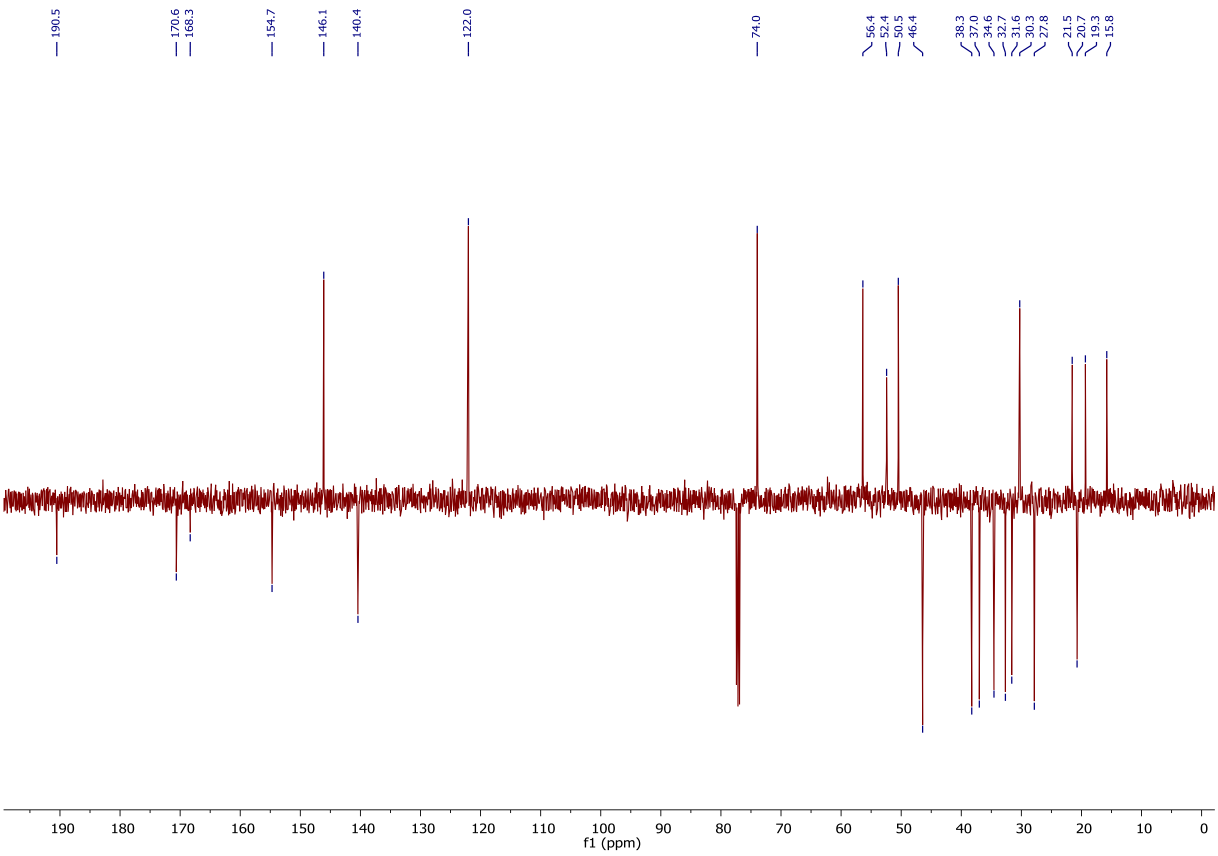

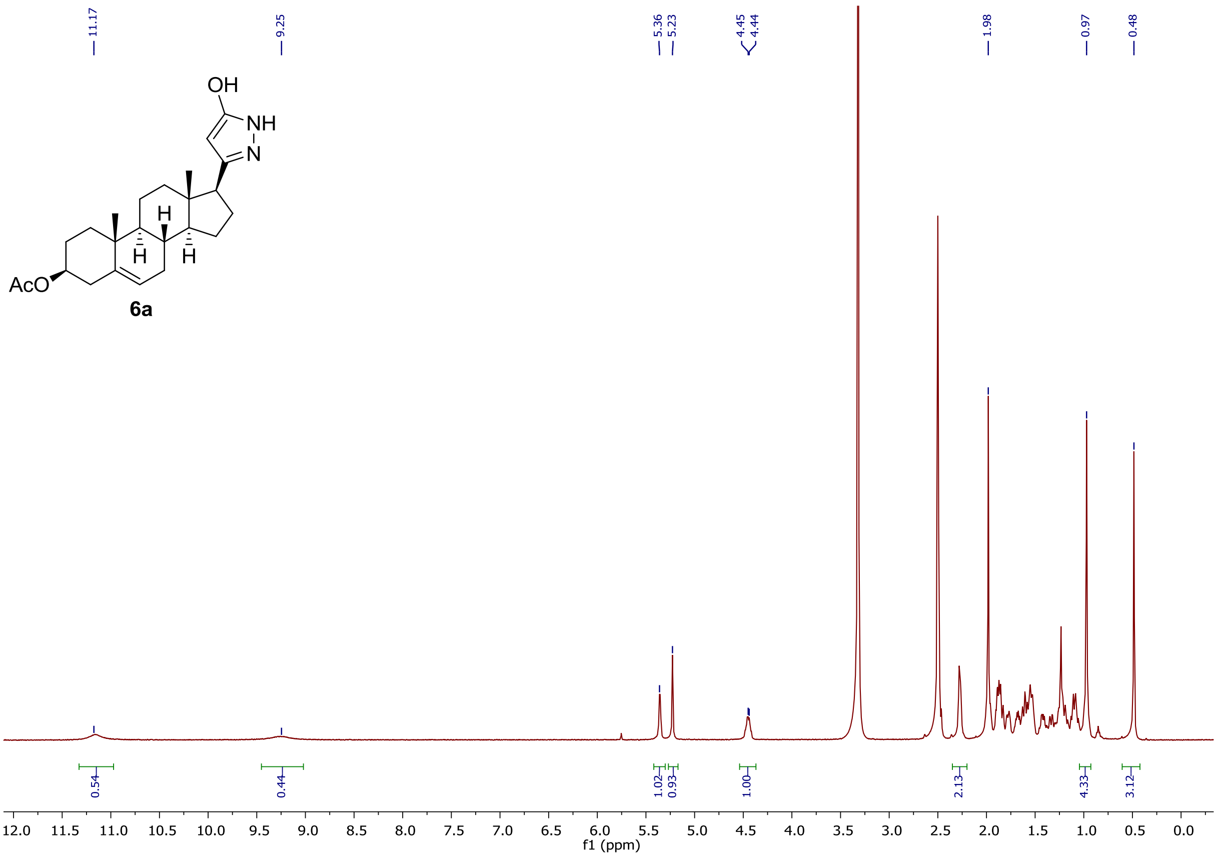

11.17
9.25
5.36
5.23
4.45
4.44
1.98
0.97
0.48
OH
NH
N
H
H
H
AcO
0.54
0.44
1.02
0.93
1.00
2.13
4.33
3.12
12.0
11.5
11.0
10.5
10.0
9.5
9.0
8.5
8.0
7.5
7.0
6.5
6.0
5.5
5.0
4.5
4.0
3.5
3.0
2.5
2.0
1.5
1.0
0.5
0.0
f1 (ppm)

6a

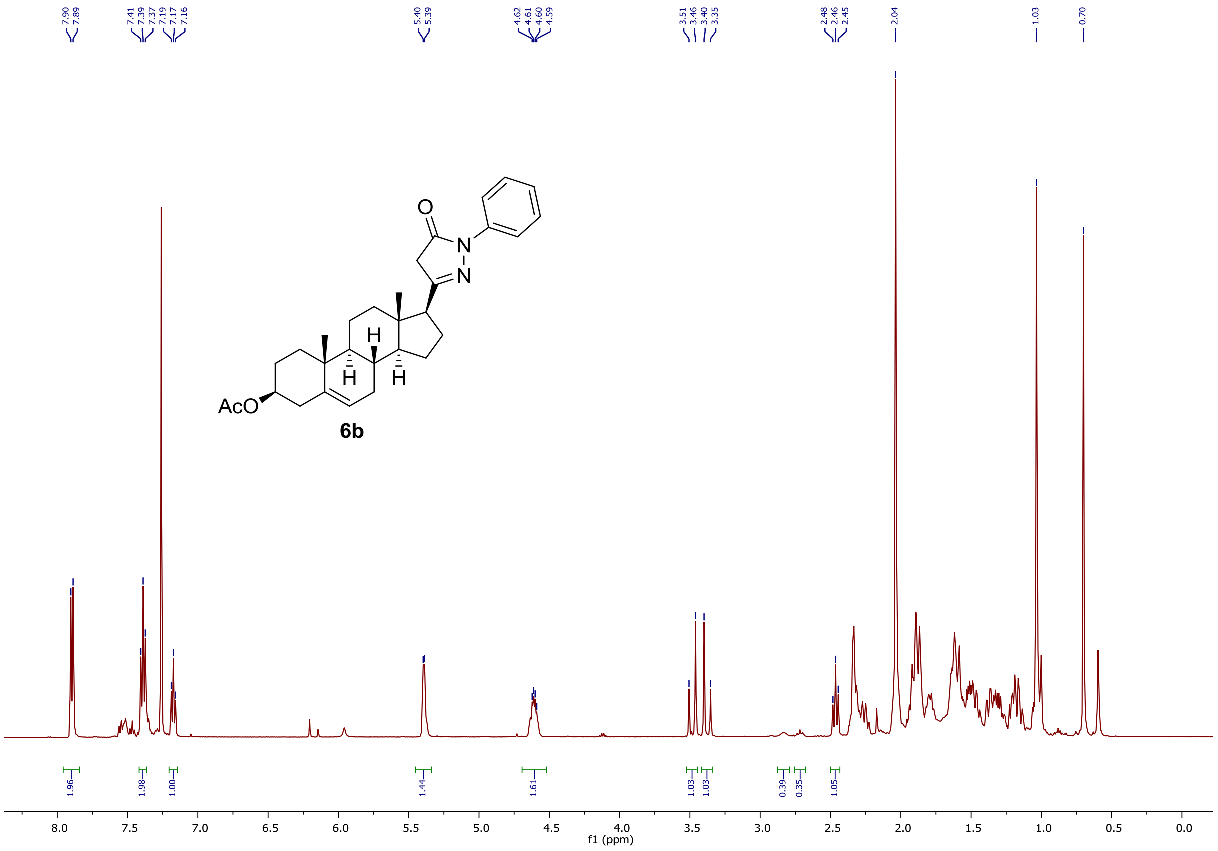
7.90
7.89
7.41
7.39
7.37
7.19
7.17
7.16
5.40
5.39
4.62
4.61
4.60
4.59
3.51
3.46
3.40
3.35
2.48
2.46
2.45
2.04
1.03
0.70
O
N
N
H
H
H
AcO
6b
1.96
1.98
1.00
1.44
1.61
1.03
1.03
0.39
0.35
1.05
8.0 7.5 7.0 6.5 6.0 5.5 5.0 4.5 4.0 3.5 3.0 2.5 2.0 1.5 1.0 0.5 0.0
f1 (ppm)

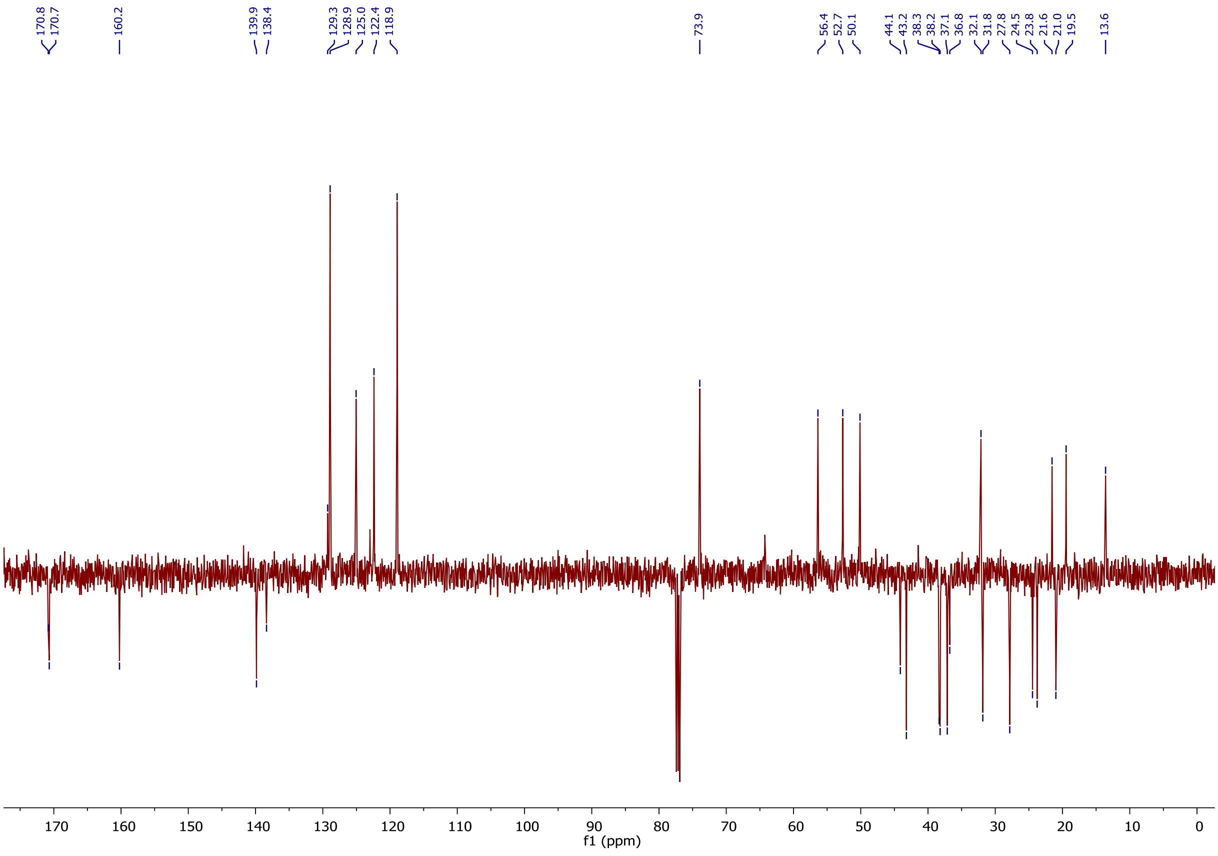
170.8
170.7
160.2
139.9
138.4
129.3
128.9
125.0
122.4
118.9
73.9
56.4
52.7
50.1
44.1
43.2
38.3
38.2
37.1
36.8
32.1
31.8
27.8
24.5
23.8
21.6
21.0
19.5
13.6
170 160 150 140 130 120 110 100 90 80 70 60 50 40 30 20 10 0
f1 (ppm)

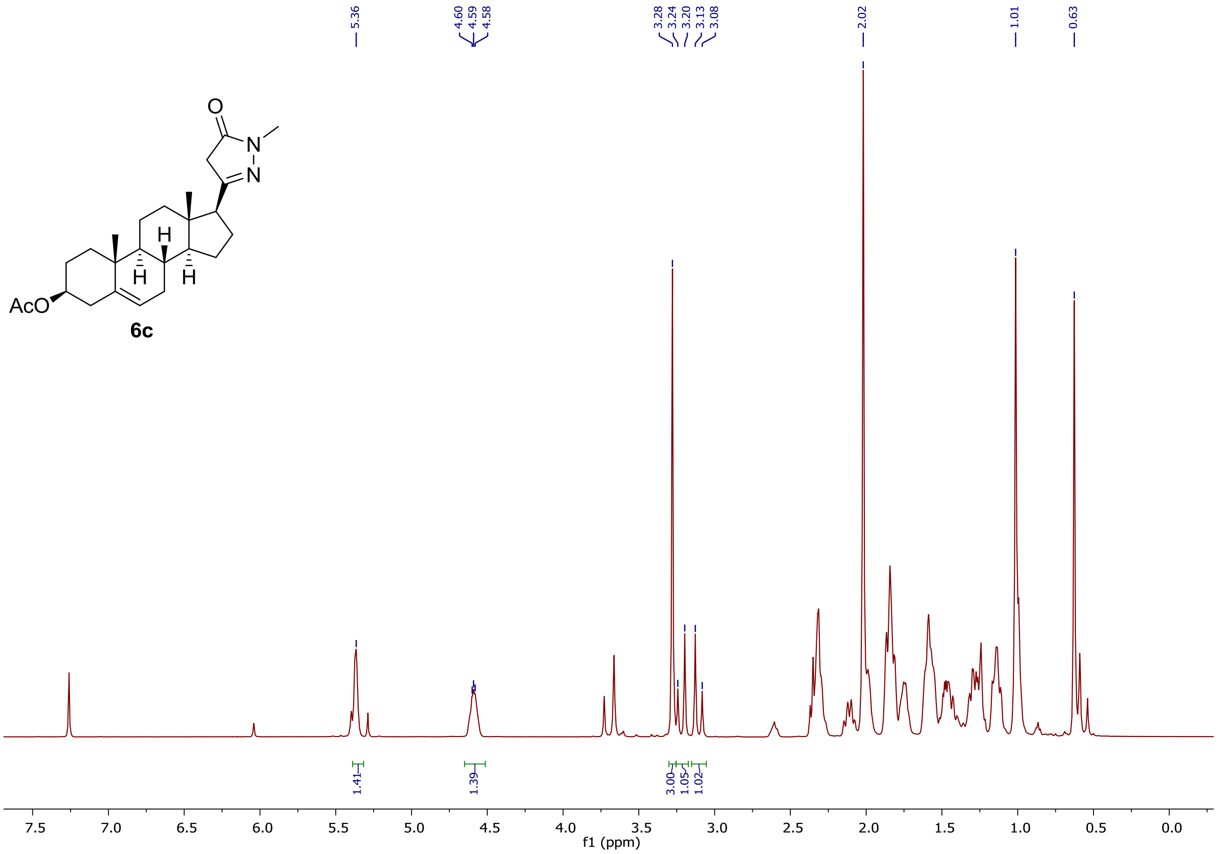
AcO
6c
5.36
4.60
4.59
4.58
3.28
3.24
3.20
3.13
3.08
2.02
1.01
0.63
1.41
1.39
3.00
1.05
1.02
7.5 7.0 6.5 6.0 5.5 5.0 4.5 4.0 3.5 3.0 2.5 2.0 1.5 1.0 0.5 0.0
f1 (ppm)

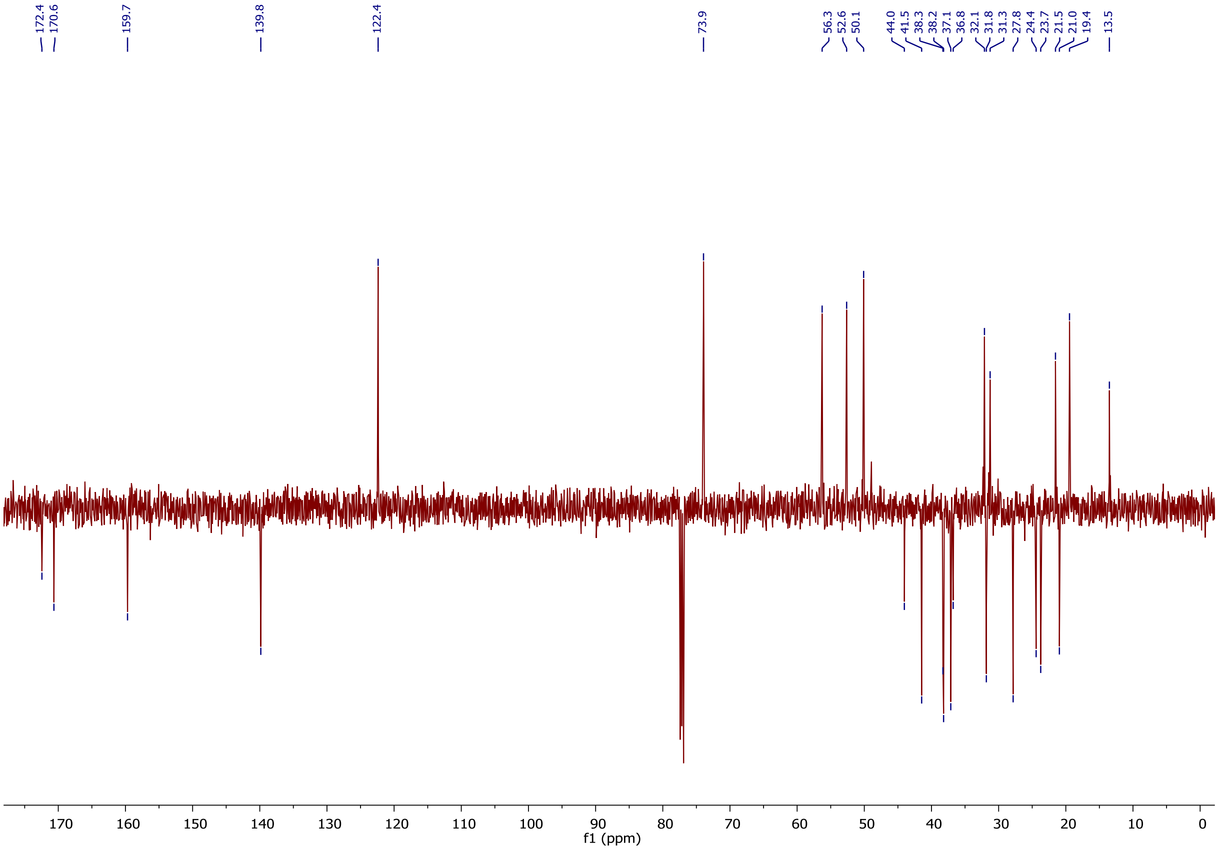
172.4
170.6
159.7
139.8
122.4
73.9
56.3
52.6
50.1
44.0
41.5
38.3
38.2
37.1
36.8
32.1
31.8
31.3
27.8
24.4
23.7
21.5
21.0
19.4
13.5
170 160 150 140 130 120 110 100 90 80 70 60 50 40 30 20 10 0
f1 (ppm)

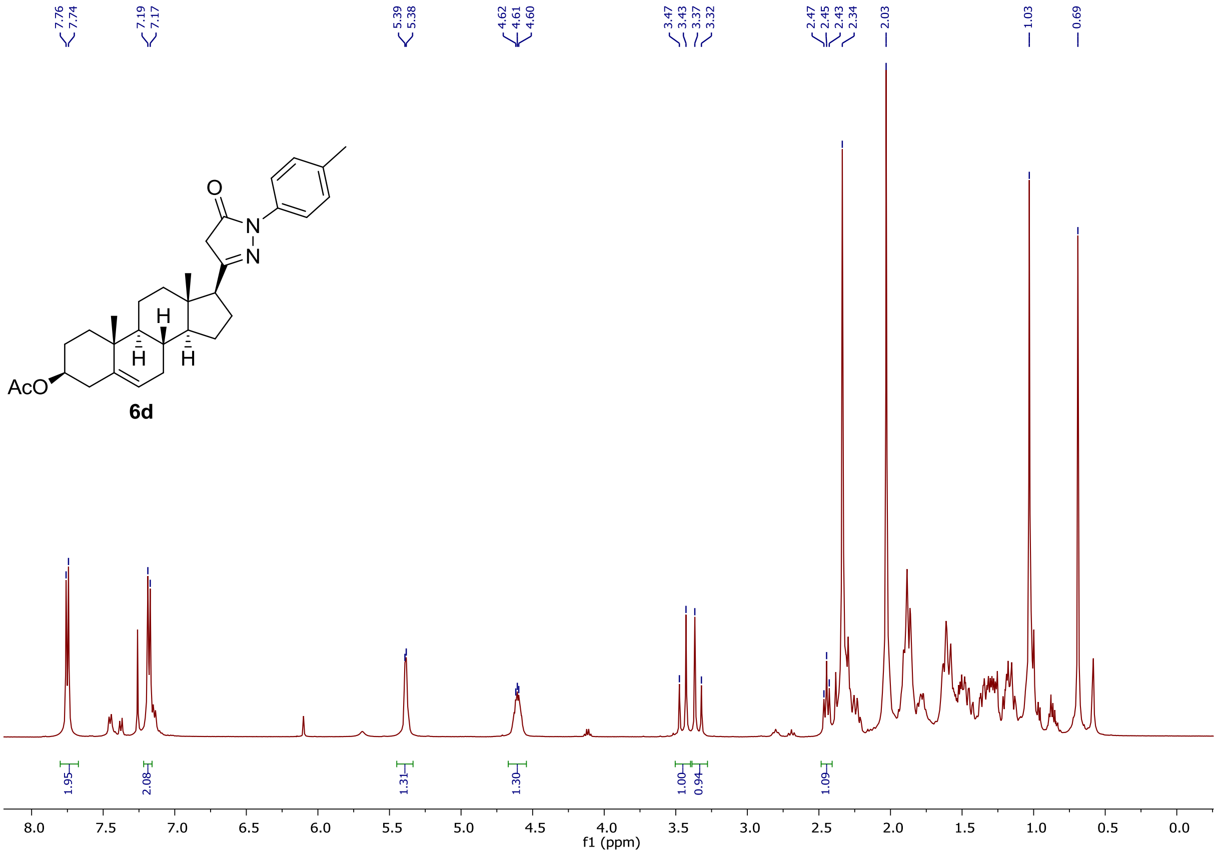
O
N
N
H
H
H
AcO
6d
7.76
7.74
7.19
7.17
5.39
5.38
4.62
4.61
4.60
3.47
3.43
3.37
3.32
2.47
2.45
2.43
2.34
2.03
1.03
0.69
1.95
2.08
1.31
1.30
1.00
0.94
1.09
8.0 7.5 7.0 6.5 6.0 5.5 5.0 4.5 4.0 3.5 3.0 2.5 2.0 1.5 1.0 0.5 0.0
f1 (ppm)

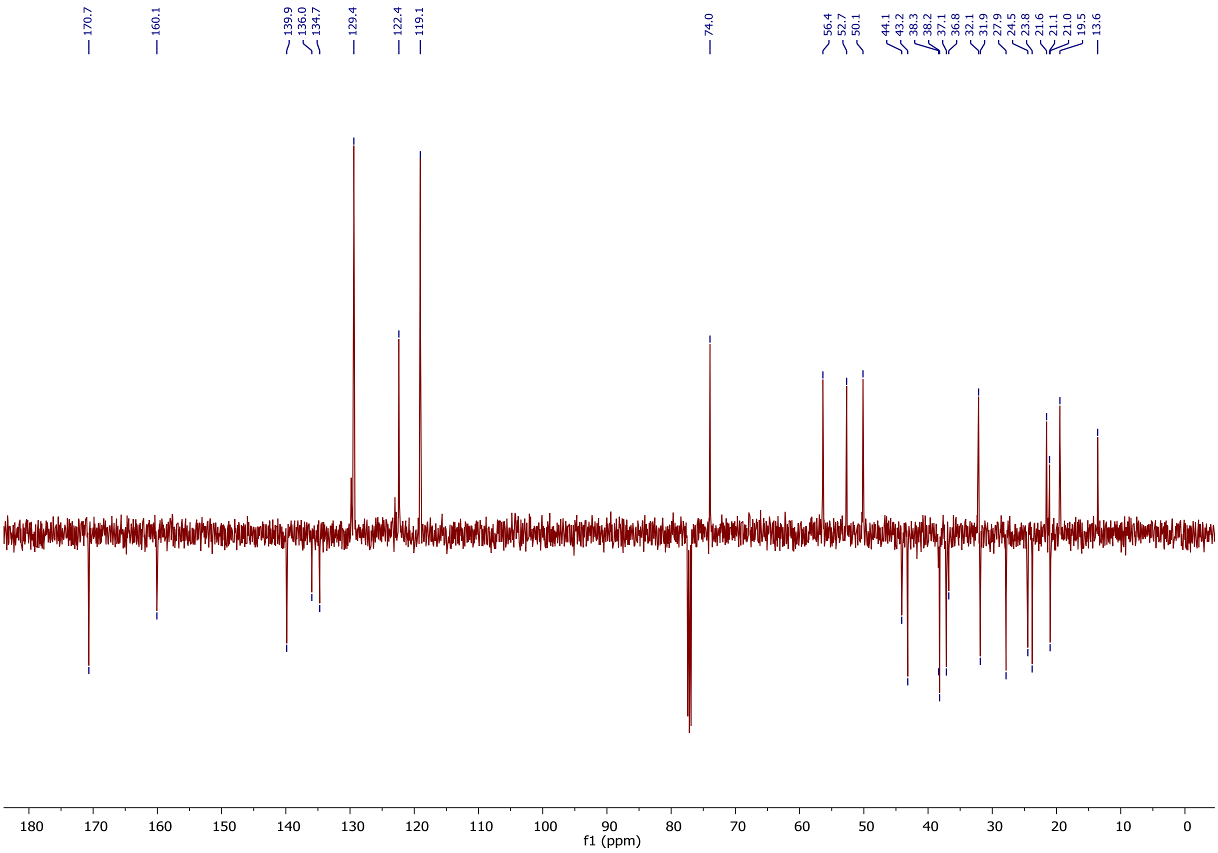
170.7
160.1
139.9
136.0
134.7
129.4
122.4
119.1
74.0
56.4
52.7
50.1
44.1
43.2
38.3
38.2
37.1
36.8
32.1
31.9
27.9
24.5
23.8
21.6
21.1
21.0
19.5
13.6
180 170 160 150 140 130 120 110 100 90 80 70 60 50 40 30 20 10 0
f1 (ppm)

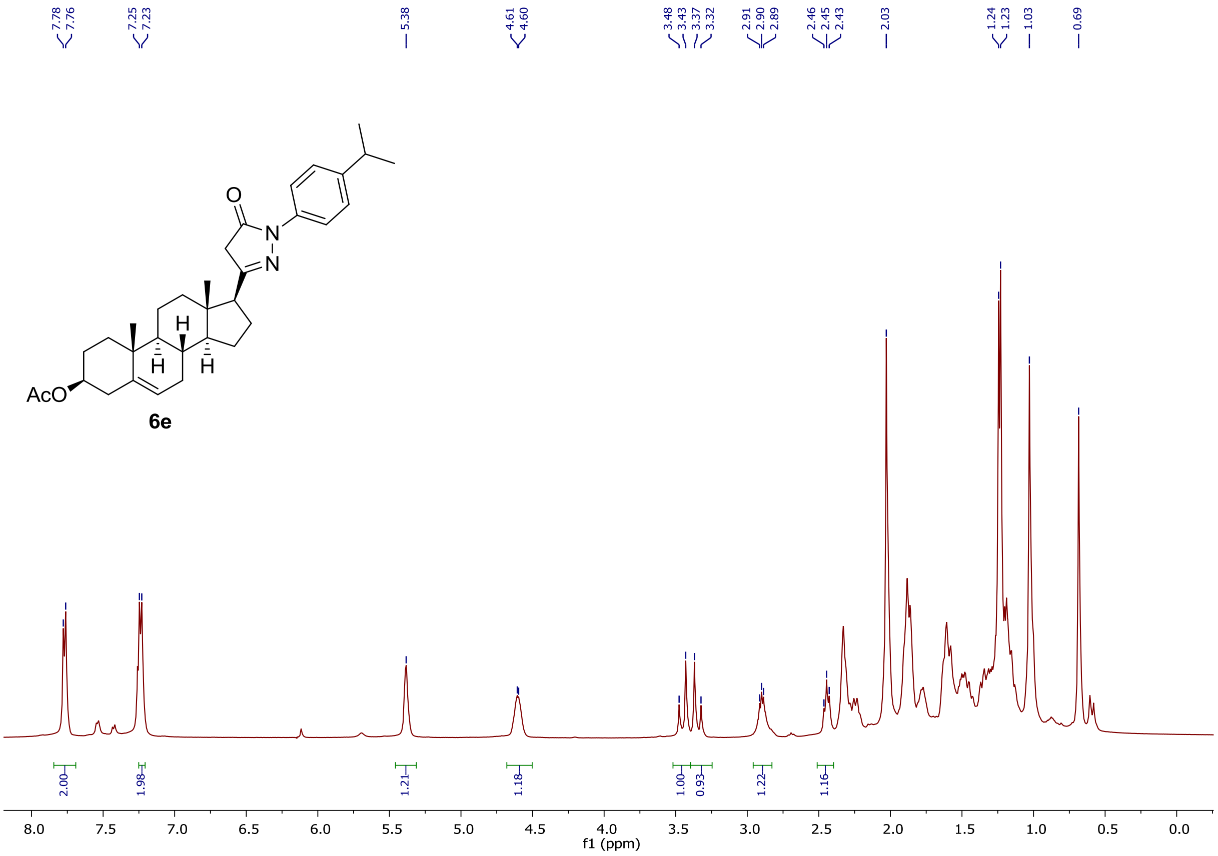
7.78
7.76
7.25
7.23
5.38
4.61
4.60
3.48
3.43
3.37
3.32
2.91
2.90
2.89
2.46
2.45
2.43
2.03
1.24
1.23
1.03
0.69
O
N
N
H
H
H
AcO
6e
2.00
1.98
1.21
1.18
1.00
0.93
1.22
1.16
8.0 7.5 7.0 6.5 6.0 5.5 5.0 4.5 4.0 3.5 3.0 2.5 2.0 1.5 1.0 0.5 0.0
f1 (ppm)

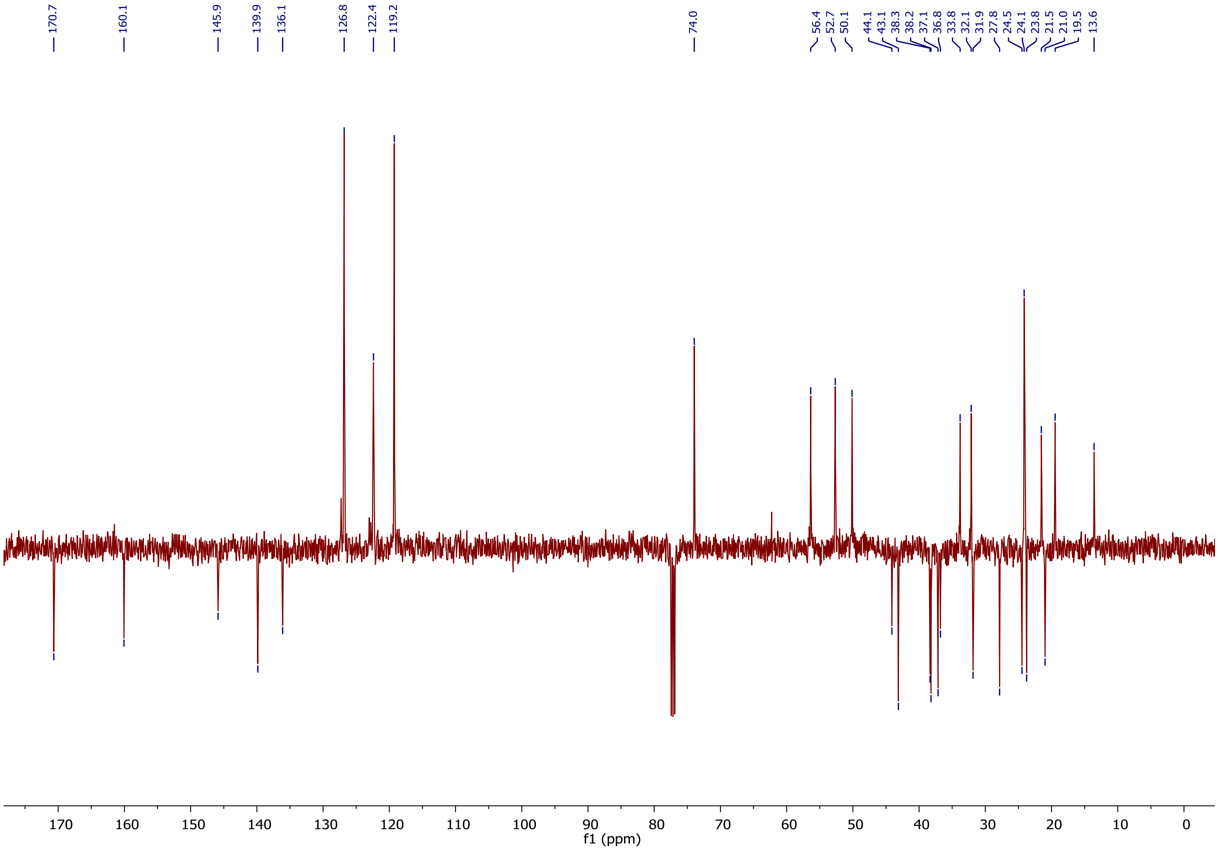
170.7
160.1
145.9
139.9
136.1
126.8
122.4
119.2
74.0
56.4
52.7
50.1
44.1
43.1
38.3
38.2
37.1
36.8
33.8
32.1
31.9
27.8
24.5
24.1
23.8
21.5
21.0
19.5
13.6
170 160 150 140 130 120 110 100 90 80 70 60 50 40 30 20 10 0
f1 (ppm)

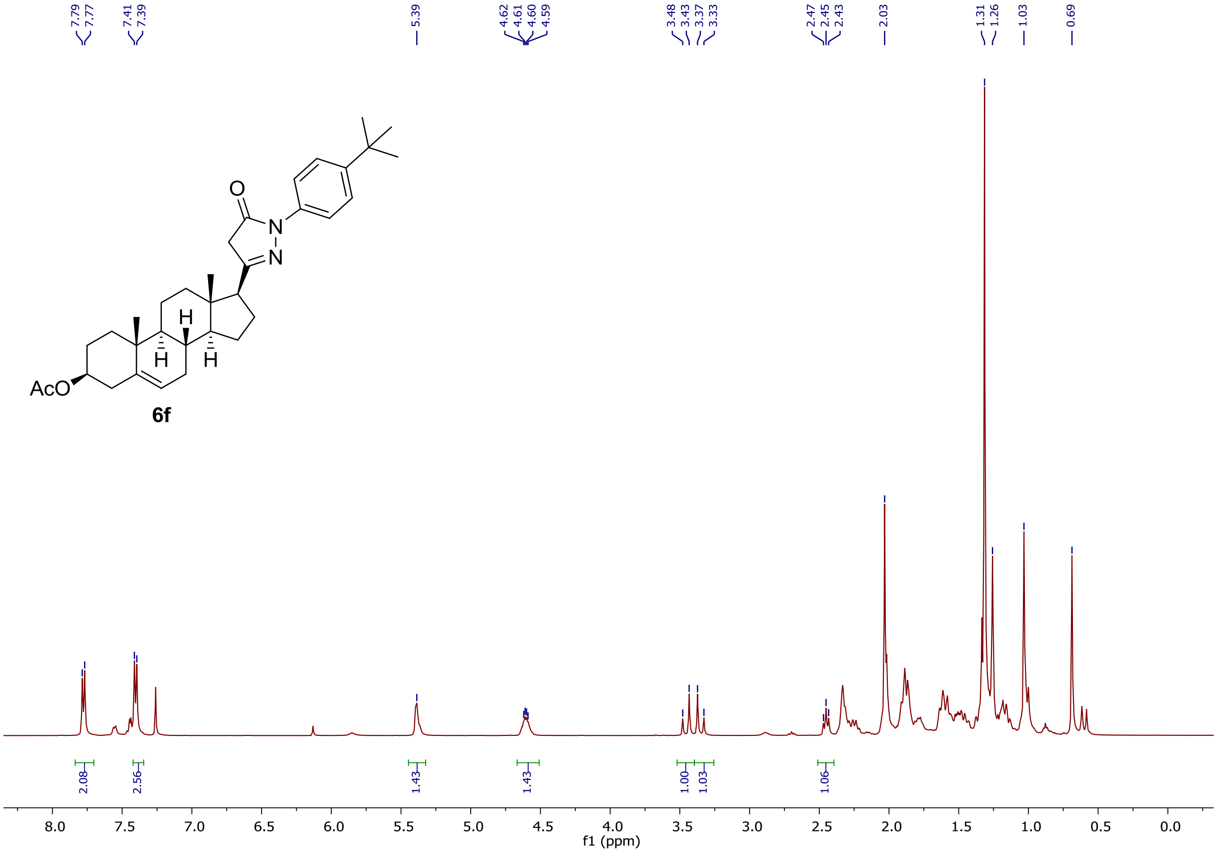
O
N
N
H
H
H
AcO
6f
7.79
7.77
7.41
7.39
5.39
4.62
4.61
4.60
4.59
3.48
3.43
3.37
3.33
2.47
2.45
2.43
2.03
1.31
1.26
1.03
0.69
2.08
2.56
1.43
1.43
1.00
1.03
1.06
8.0 7.5 7.0 6.5 6.0 5.5 5.0 4.5 4.0 3.5 3.0 2.5 2.0 1.5 1.0 0.5 0.0
f1 (ppm)

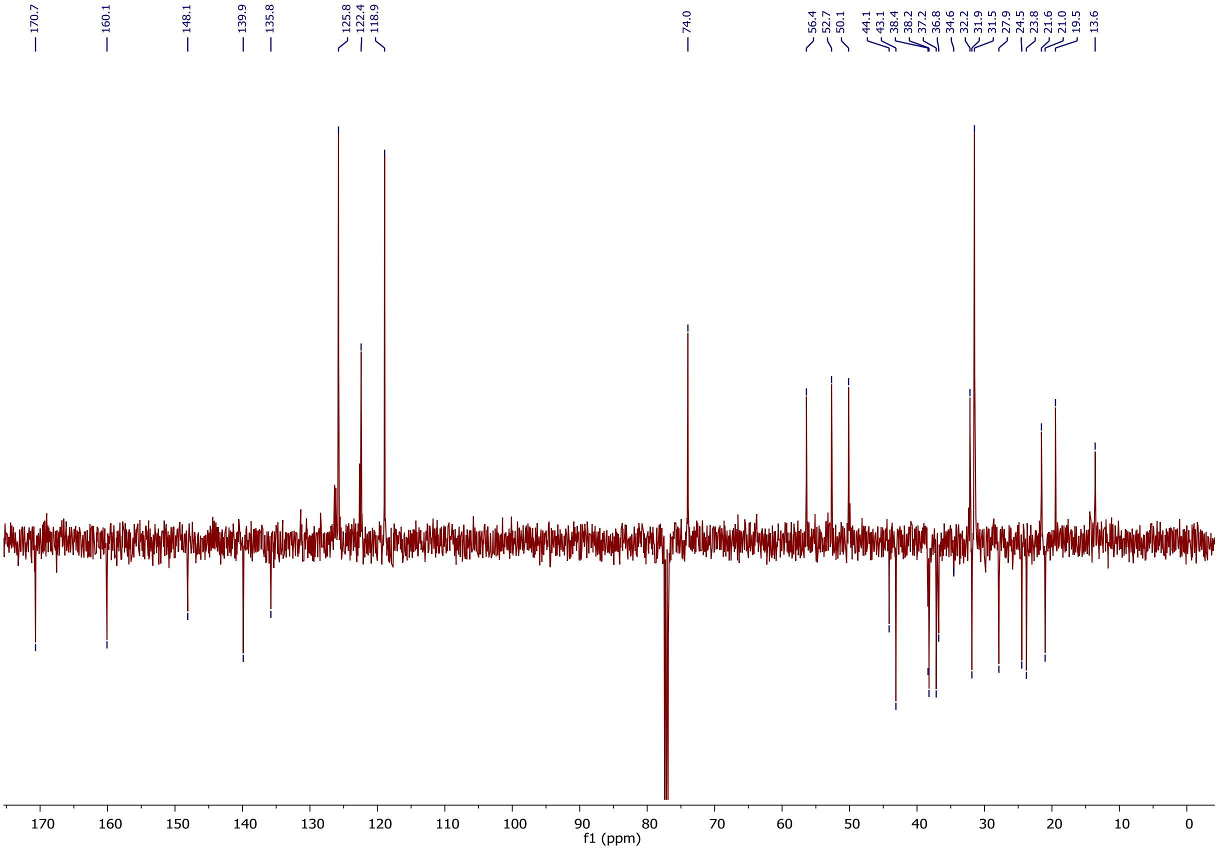
170.7
160.1
148.1
139.9
135.8
125.8
122.4
118.9
74.0
56.4
52.7
50.1
44.1
43.1
38.4
38.2
37.2
36.8
34.6
32.2
31.9
31.5
27.9
24.5
23.8
21.6
21.0
19.5
13.6
170 160 150 140 130 120 110 100 90 80 70 60 50 40 30 20 10 0
f1 (ppm)

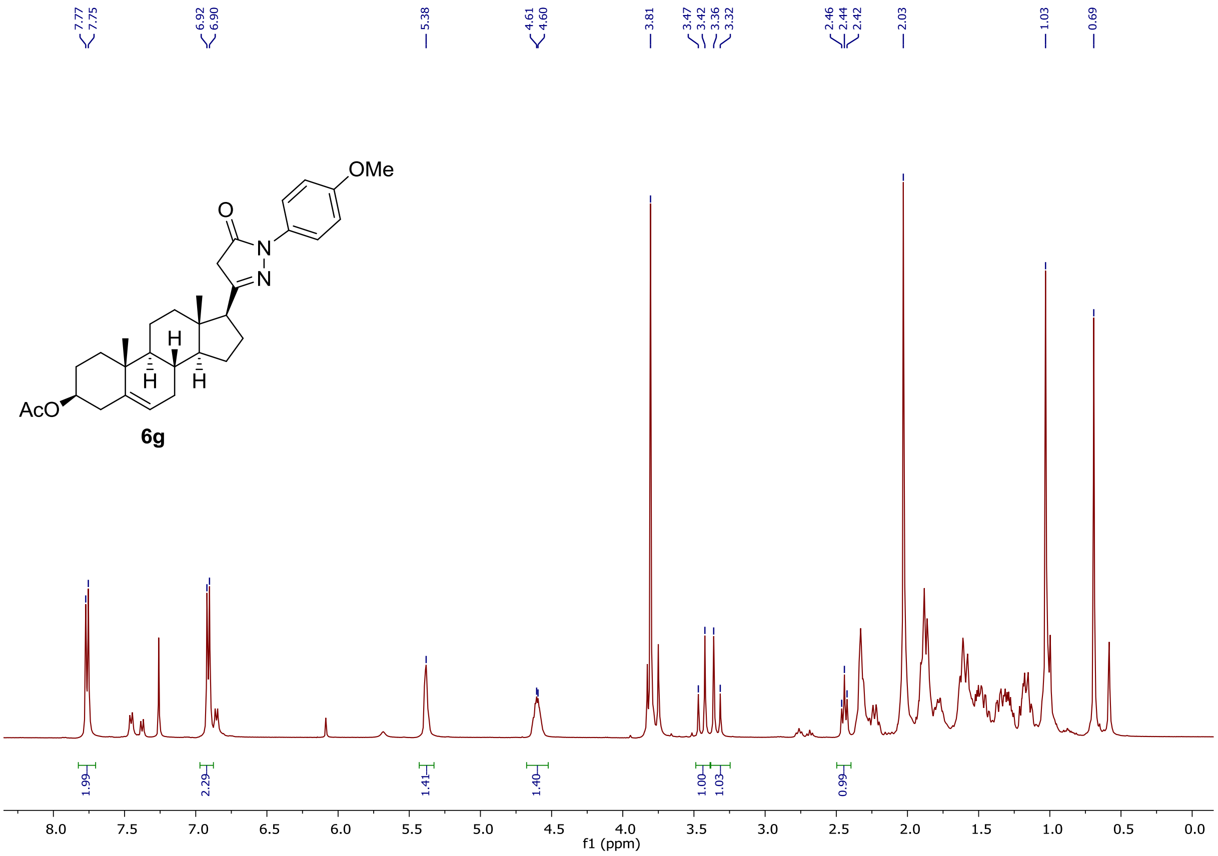
7.77
7.75
6.92
6.90
5.38
4.61
4.60
3.81
3.47
3.42
3.36
3.32
2.46
2.44
2.42
2.03
1.03
0.69
OMe
O
N
N
H
H
H
AcO
6g
1.99
2.29
1.41
1.40
1.00
1.03
0.99
8.0 7.5 7.0 6.5 6.0 5.5 5.0 4.5 4.0 3.5 3.0 2.5 2.0 1.5 1.0 0.5 0.0
f1 (ppm)

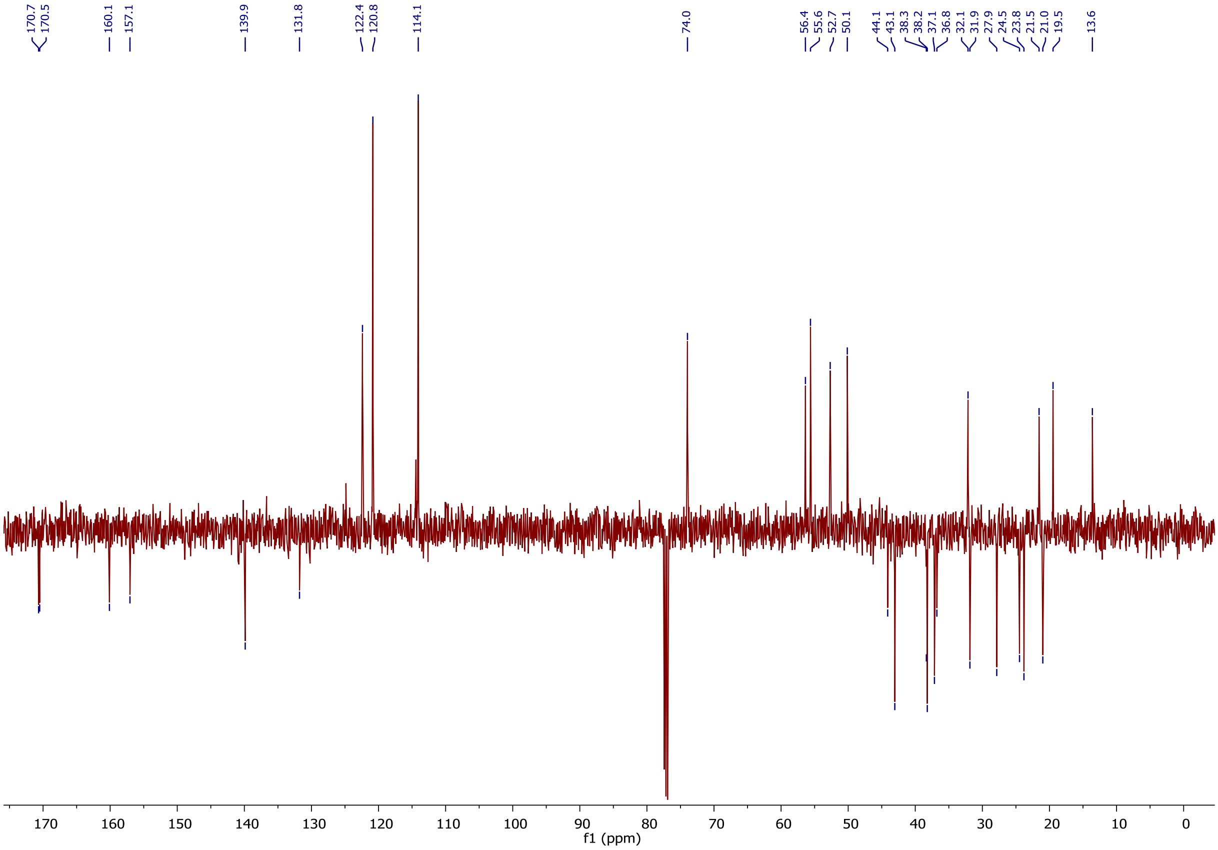
170.7
170.5
160.1
157.1
139.9
131.8
122.4
120.8
114.1
74.0
56.4
55.6
52.7
50.1
44.1
43.1
38.3
38.2
37.1
36.8
32.1
31.9
27.9
24.5
23.8
21.5
21.0
19.5
13.6
170 160 150 140 130 120 110 100 90 80 70 60 50 40 30 20 10 0
f1 (ppm)

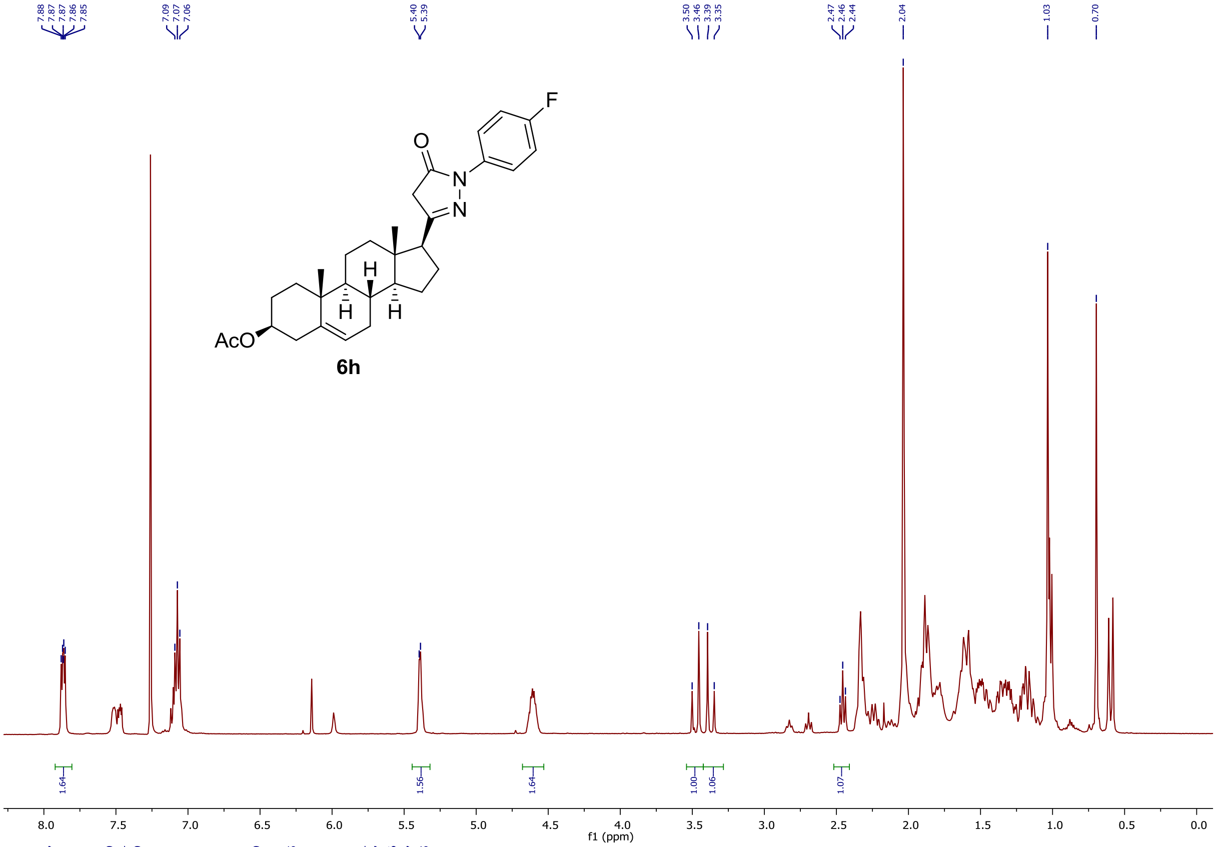
AcO
6h
f1 (ppm)

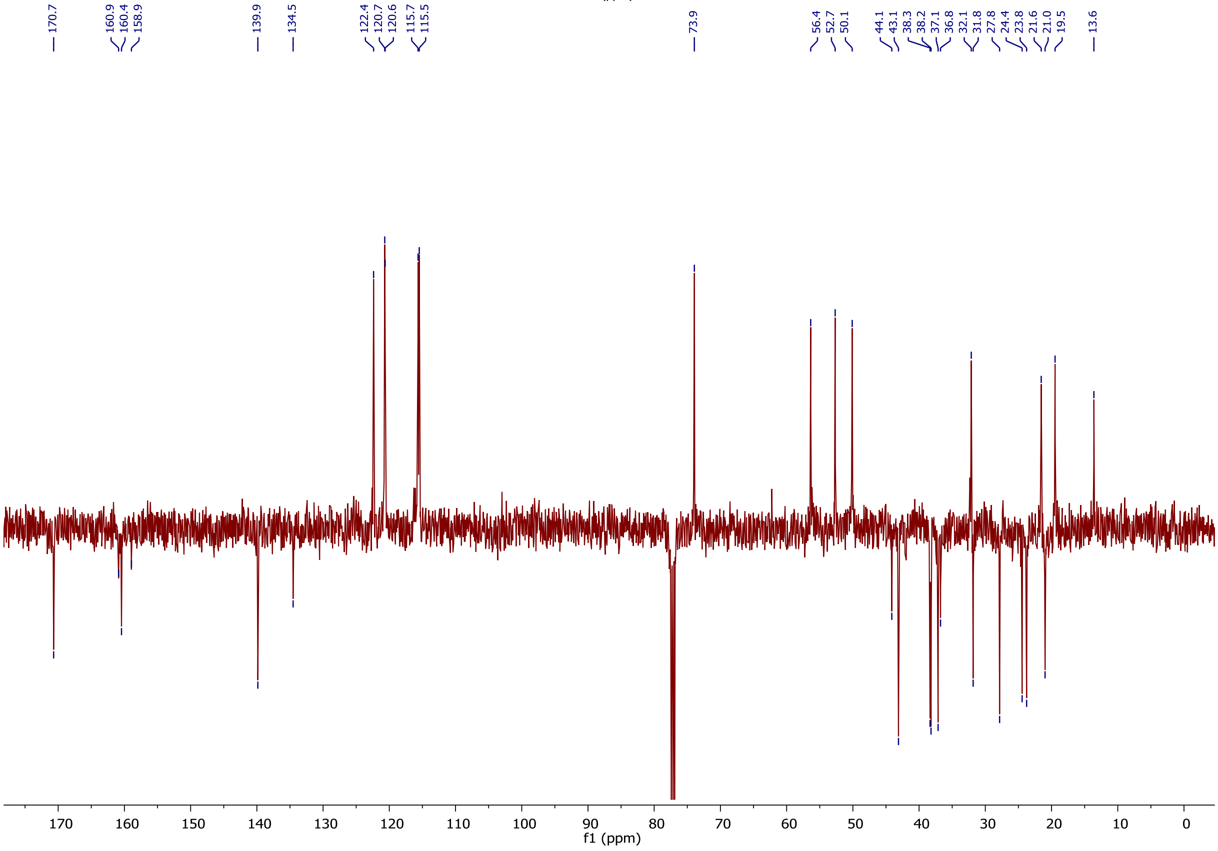
f1 (ppm)

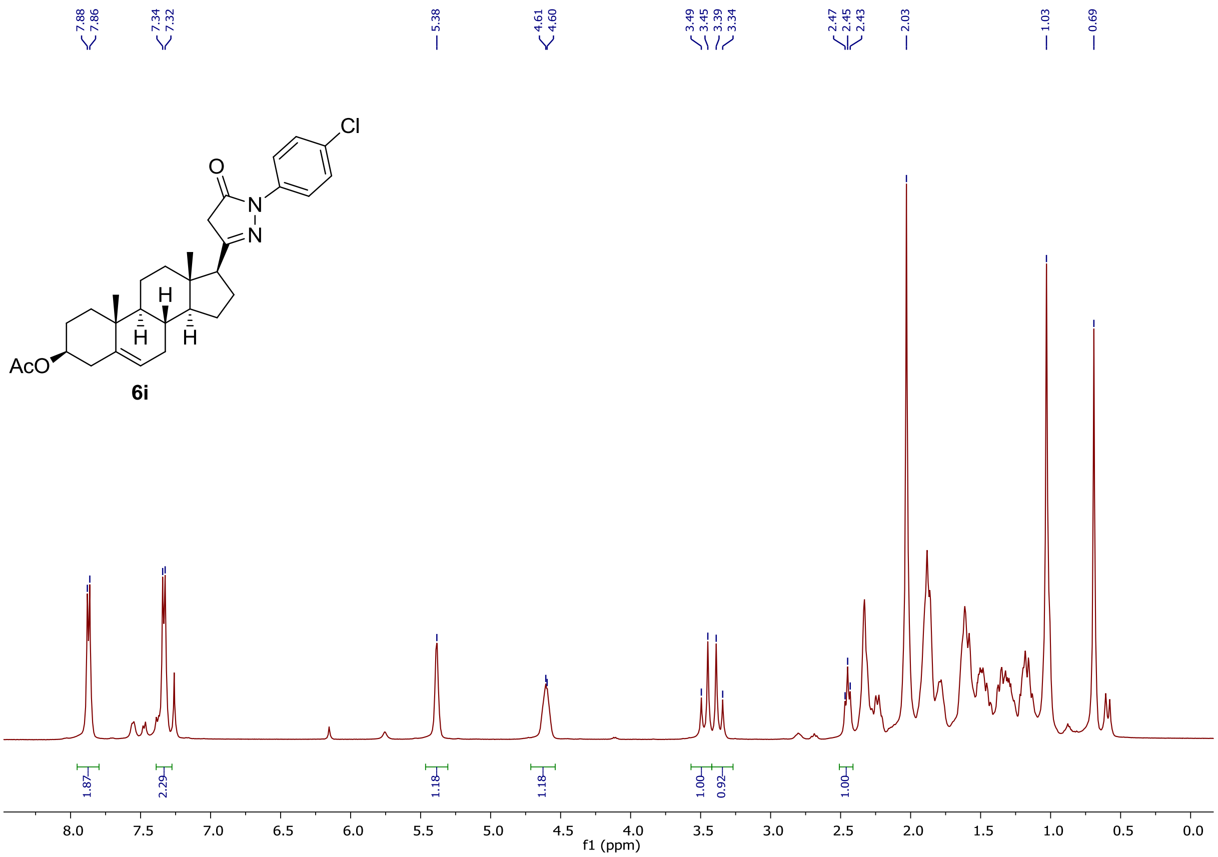
7.88
7.86
7.34
7.32
5.38
4.61
4.60
3.49
3.45
3.39
3.34
2.47
2.45
2.43
2.03
1.03
0.69
Cl
O
N
N
H
H
H
AcO
6i
1.87
2.29
1.18
1.18
1.00
0.92
1.00
8.0
7.5
7.0
6.5
6.0
5.5
5.0
4.5
4.0
3.5
3.0
2.5
2.0
1.5
1.0
0.5
0.0
f1 (ppm)

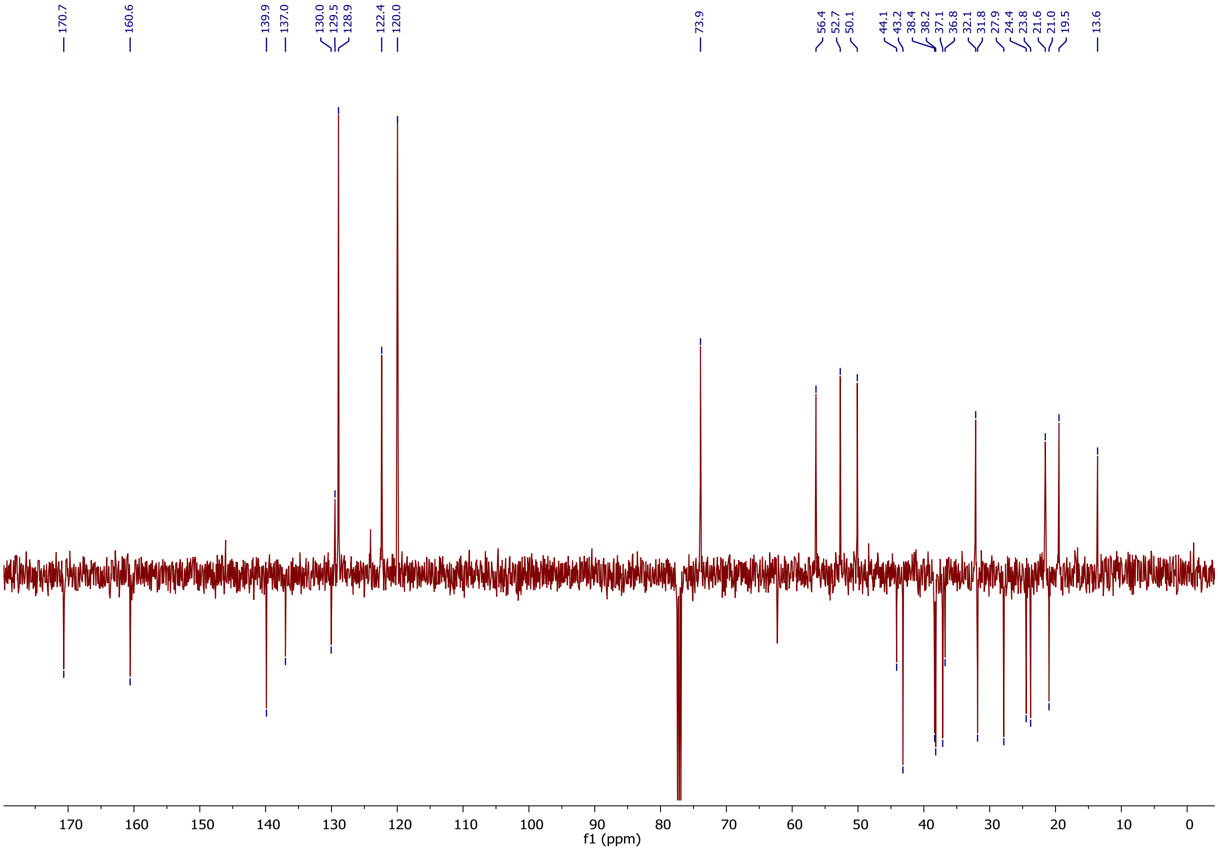
170.7
160.6
139.9
137.0
130.0
129.5
128.9
122.4
120.0
73.9
56.4
52.7
50.1
44.1
43.2
38.4
38.2
37.1
36.8
32.1
31.8
27.9
24.4
23.8
21.6
21.0
19.5
13.6
170
160
150
140
130
120
110
100
90
80
70
60
50
40
30
20
10
0
f1 (ppm)

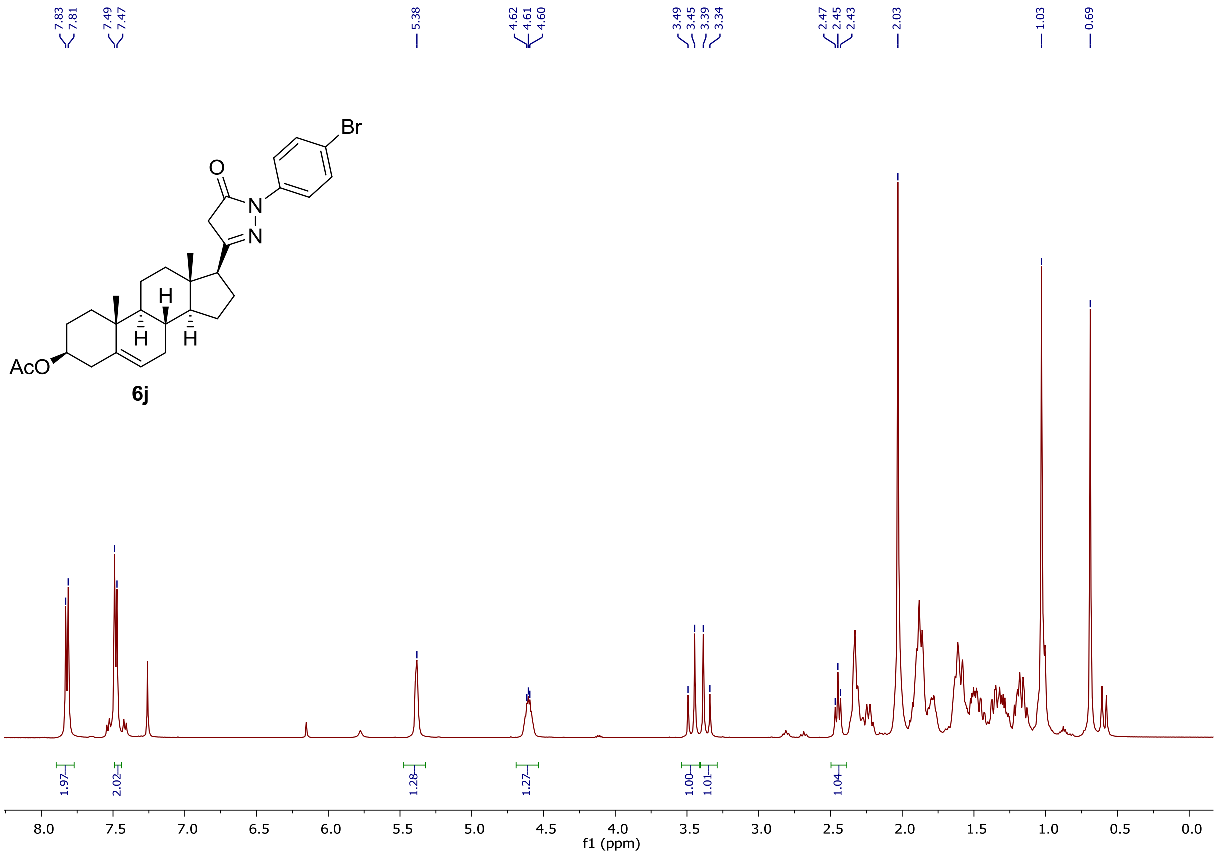
7.83
7.81
7.49
7.47
5.38
4.62
4.61
4.60
3.49
3.45
3.39
3.34
2.47
2.45
2.43
2.03
1.03
0.69
Br
O
N
N
H
H
H
AcO
6j
1.97
2.02
1.28
1.27
1.00
1.01
1.04
8.0
7.5
7.0
6.5
6.0
5.5
5.0
4.5
4.0
3.5
3.0
2.5
2.0
1.5
1.0
0.5
0.0
f1 (ppm)

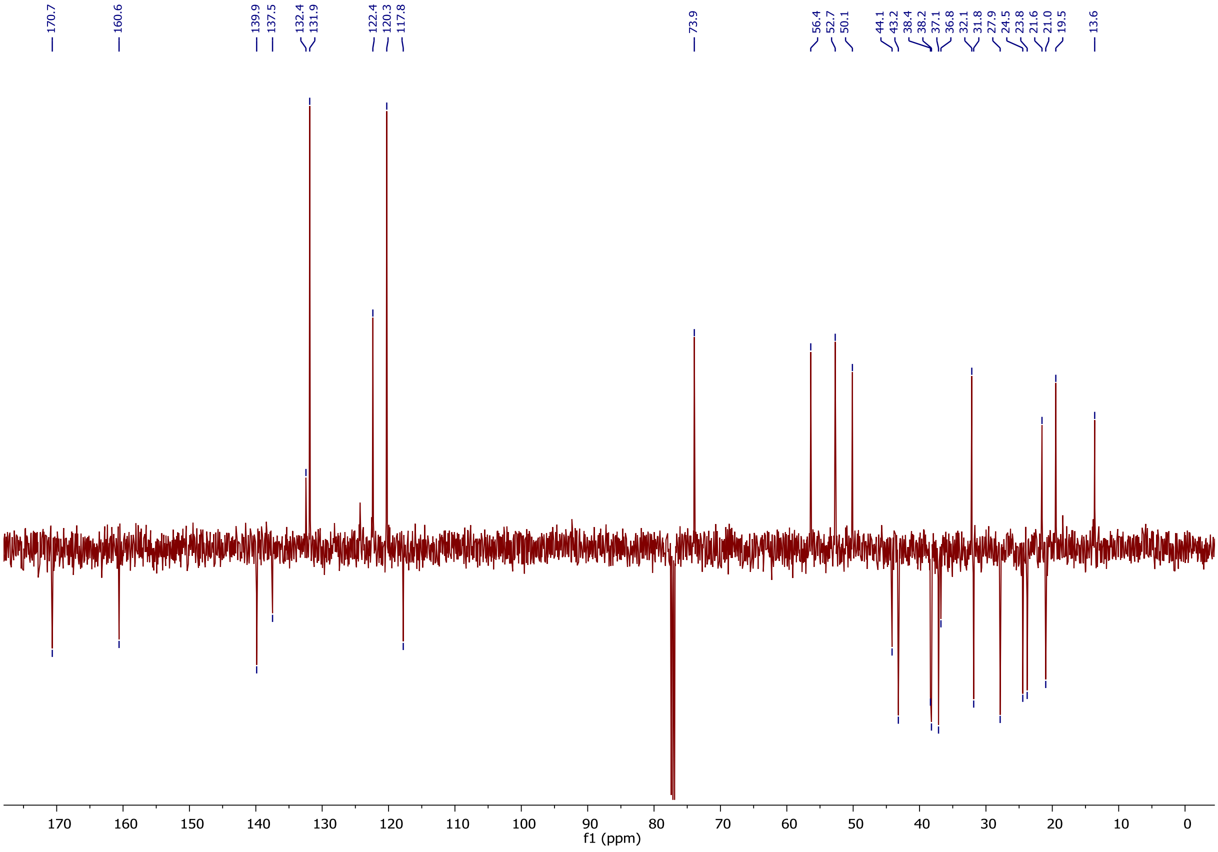
170.7
160.6
139.9
137.5
132.4
131.9
122.4
120.3
117.8
73.9
56.4
52.7
50.1
44.1
43.2
38.4
38.2
37.1
36.8
32.1
31.8
27.9
24.5
23.8
21.6
21.0
19.5
13.6
170
160
150
140
130
120
110
100
90
80
70
60
50
40
30
20
10
0
f1 (ppm)

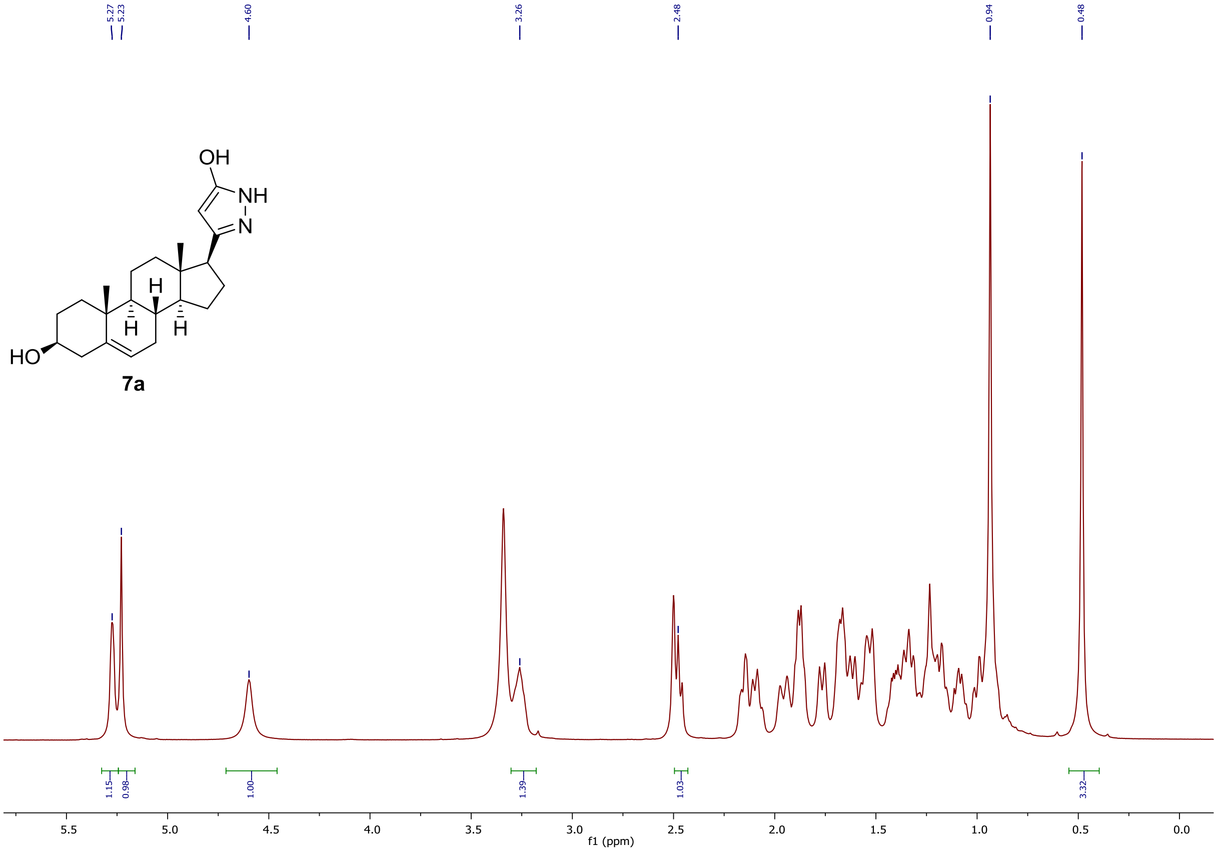
5.27
5.23
4.60
3.26
2.48
0.94
0.48
OH
NH
N
H
H
H
HO
7a
1.15
0.98
1.00
1.39
1.03
3.32
5.5
5.0
4.5
4.0
3.5
3.0
2.5
2.0
1.5
1.0
0.5
0.0
f1 (ppm)

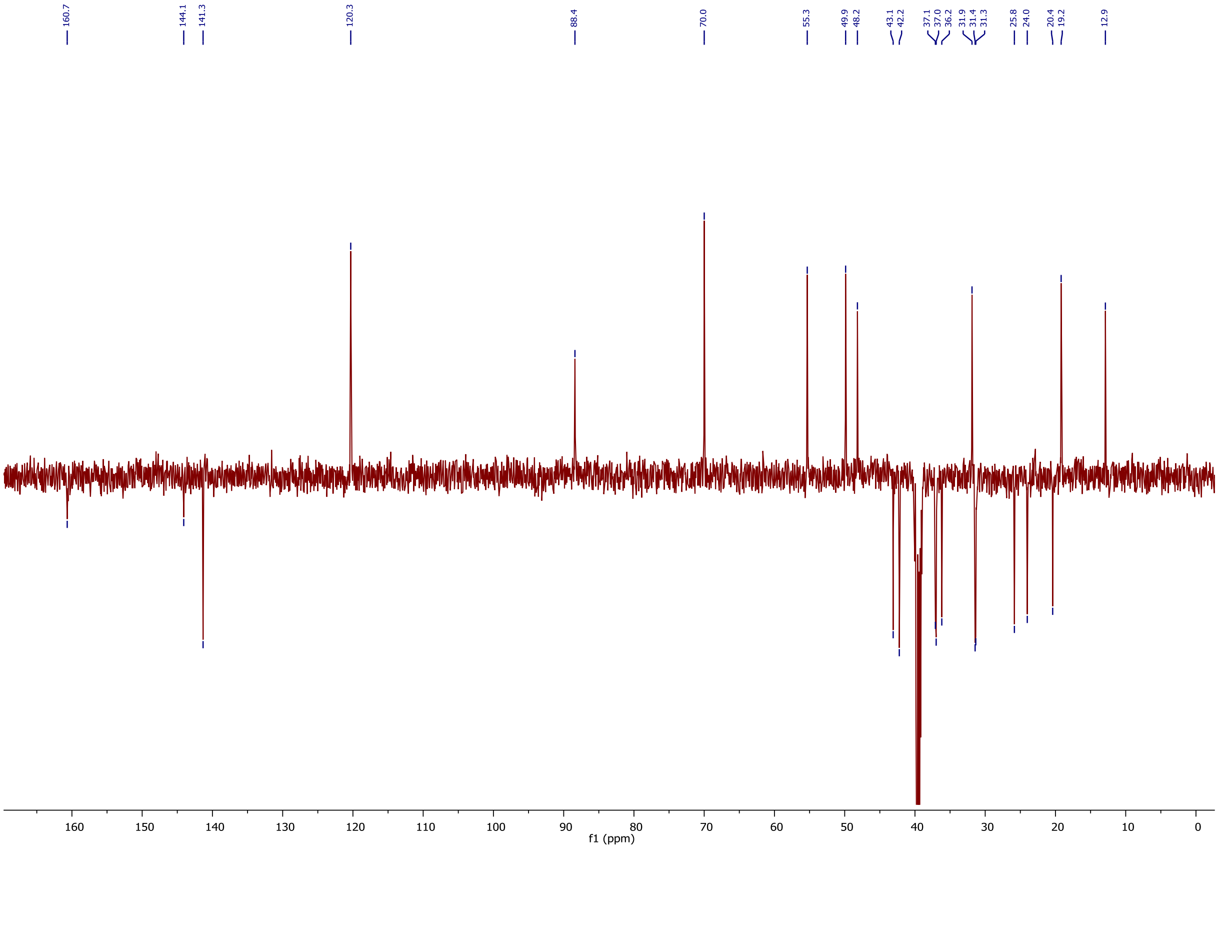
160.7
144.1
141.3
120.3
88.4
70.0
55.3
49.9
48.2
43.1
42.2
37.1
37.0
36.2
31.9
31.4
31.3
25.8
24.0
20.4
19.2
12.9
160
150
140
130
120
110
100
90
80
70
60
50
40
30
20
10
0
f1 (ppm)

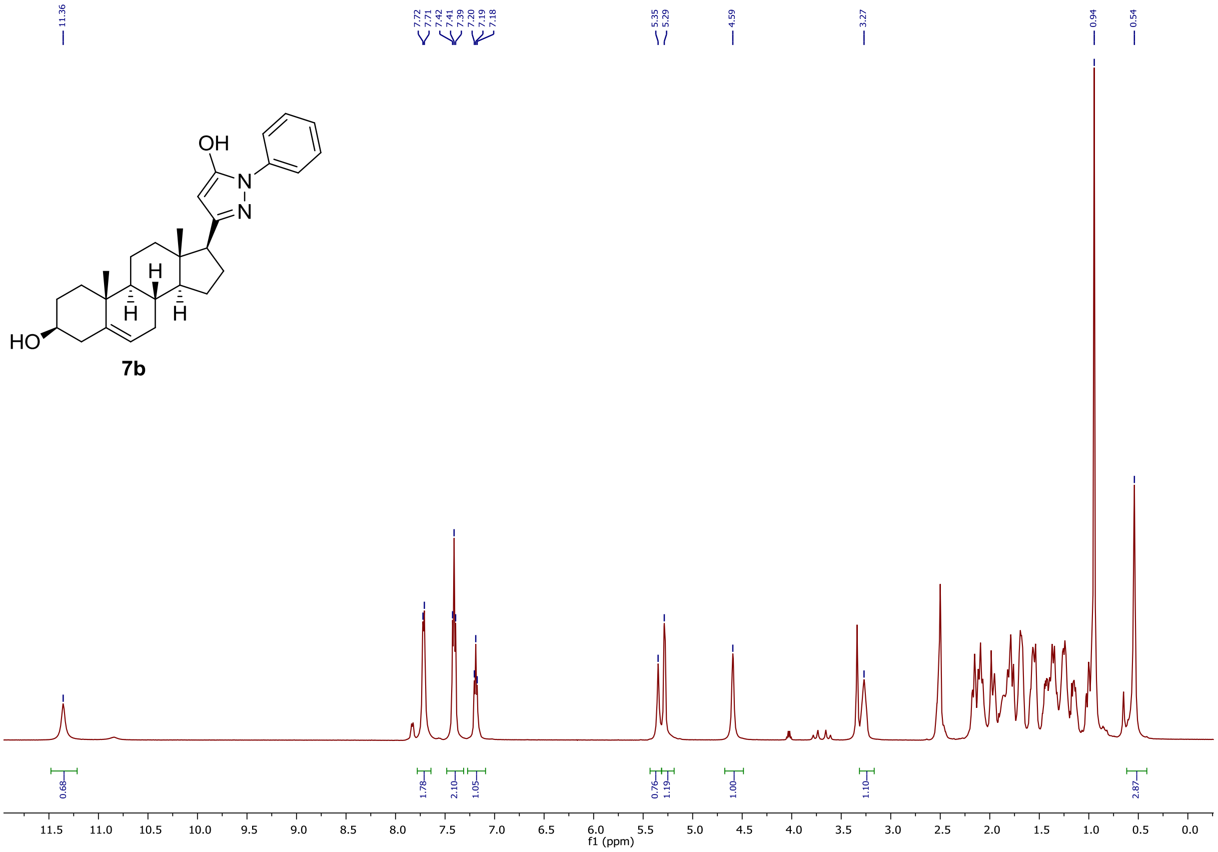

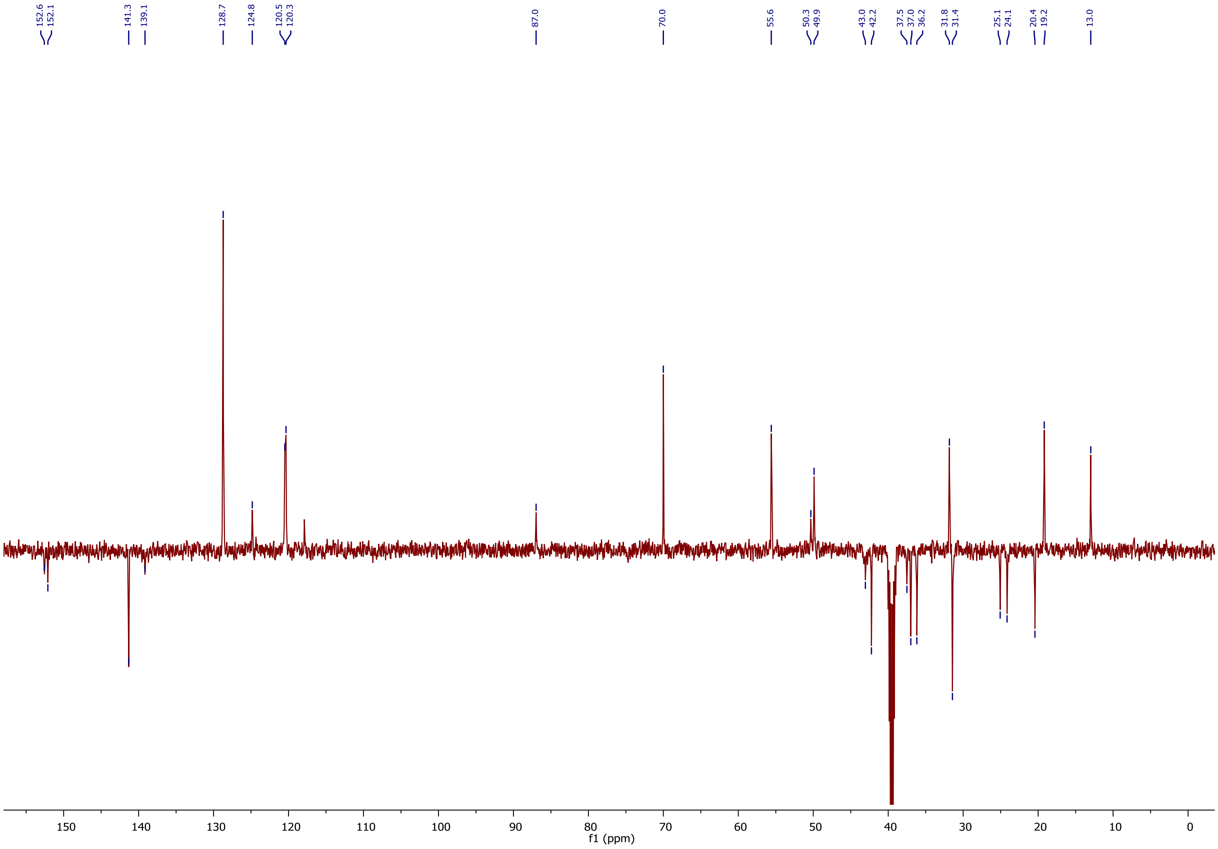

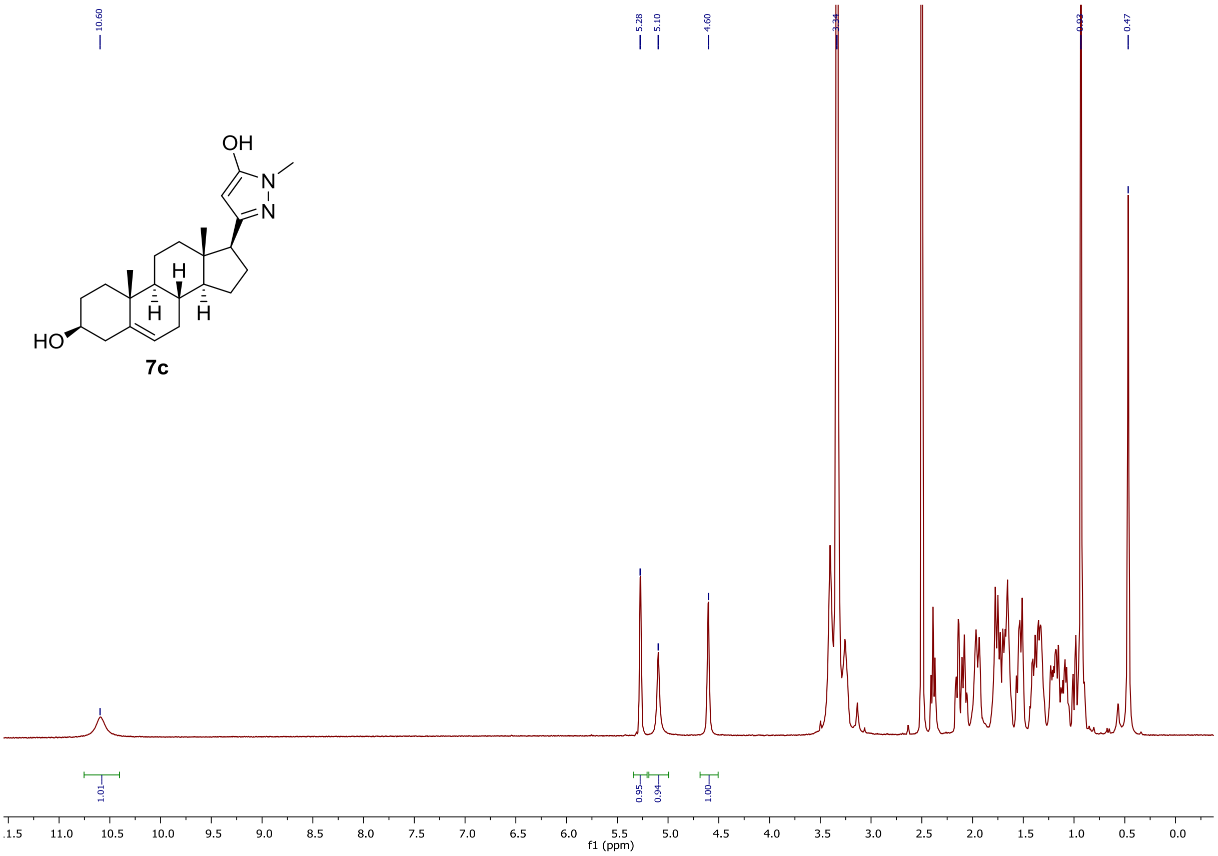
10.60
5.28
5.10
4.60
3.34
0.92
0.47
OH
N
N
H
H
H
HO
7c
1.01
0.95
0.94
1.00
11.0 10.5 10.0 9.5 9.0 8.5 8.0 7.5 7.0 6.5 6.0 5.5 5.0 4.5 4.0 3.5 3.0 2.5 2.0 1.5 1.0 0.5 0.0
f1 (ppm)

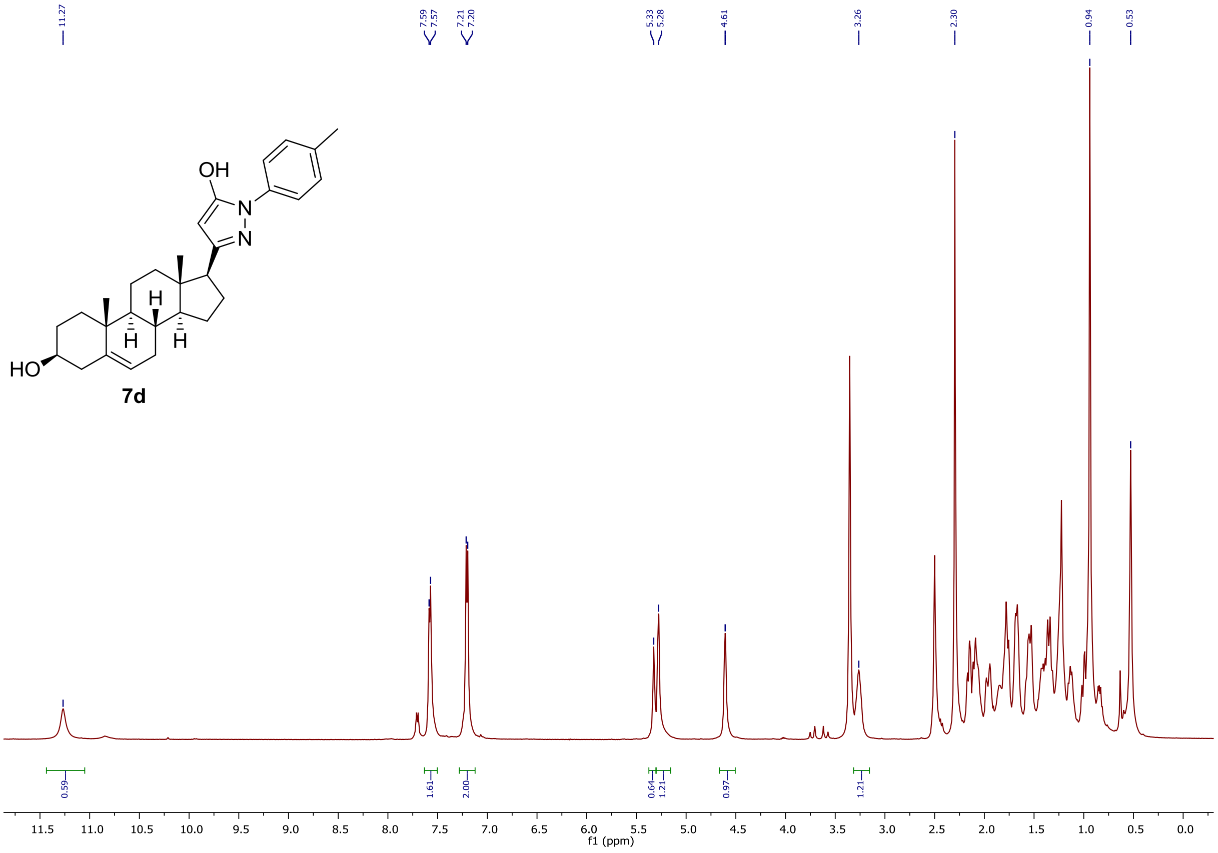
OH
N
N
H
H
H
HO
7d
11.27
7.59
7.57
7.21
7.20
5.33
5.28
4.61
3.26
2.30
0.94
0.53
0.59
1.61
2.00
0.64
1.21
0.97
1.21
f1 (ppm)

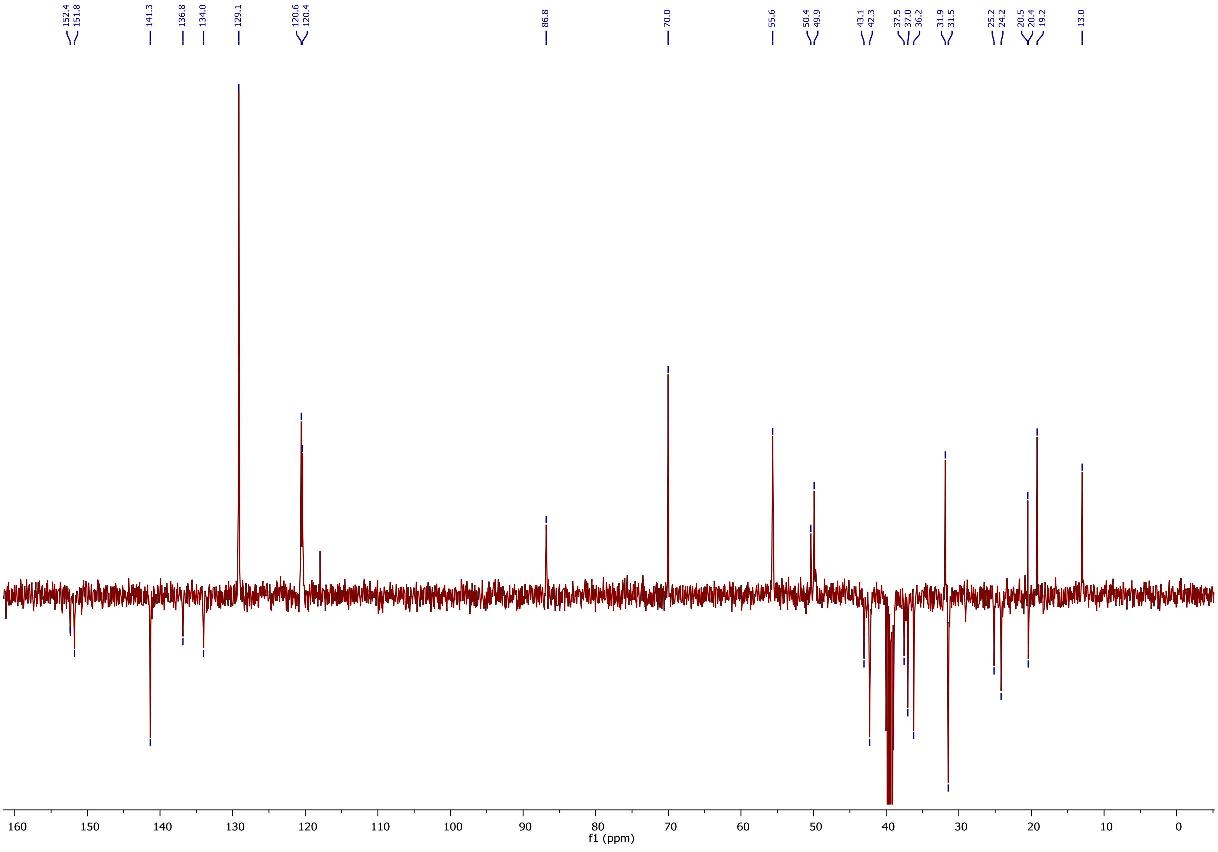
152.4
151.8
141.3
136.8
134.0
129.1
120.6
120.4
86.8
70.0
55.6
50.4
49.9
43.1
42.3
37.5
37.0
36.2
31.9
31.5
25.2
24.2
20.5
20.4
19.2
13.0
f1 (ppm)

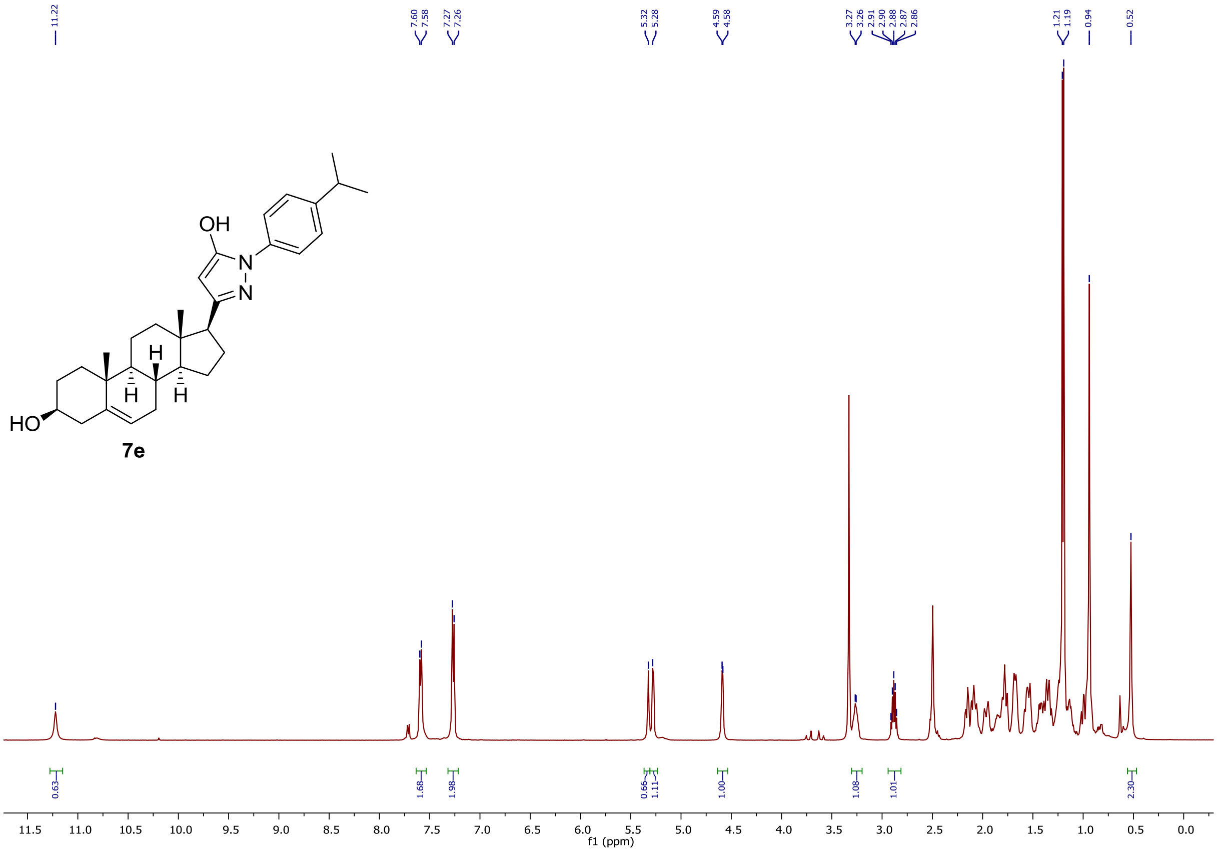
7e
11.22
7.60
7.58
7.27
7.26
5.32
5.28
4.59
4.58
3.27
3.26
2.91
2.90
2.88
2.87
2.86
1.21
1.19
0.94
0.52
0.63
1.68
1.98
0.66
1.11
1.00
1.08
1.01
2.30
f1 (ppm)

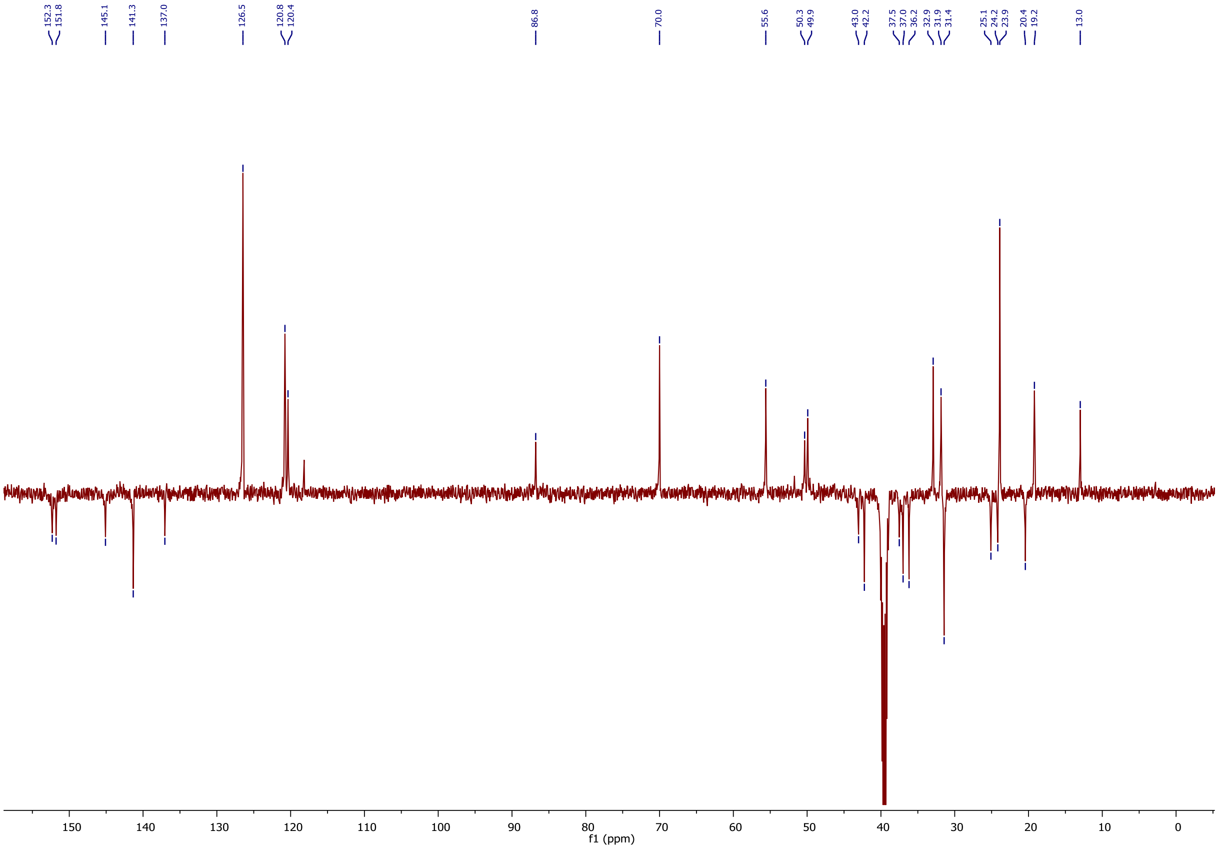
152.3
151.8
145.1
141.3
137.0
126.5
120.8
120.4
86.8
70.0
55.6
50.3
49.9
43.0
42.2
37.5
37.0
36.2
32.9
31.9
31.4
25.1
24.2
23.9
20.4
19.2
13.0
f1 (ppm)

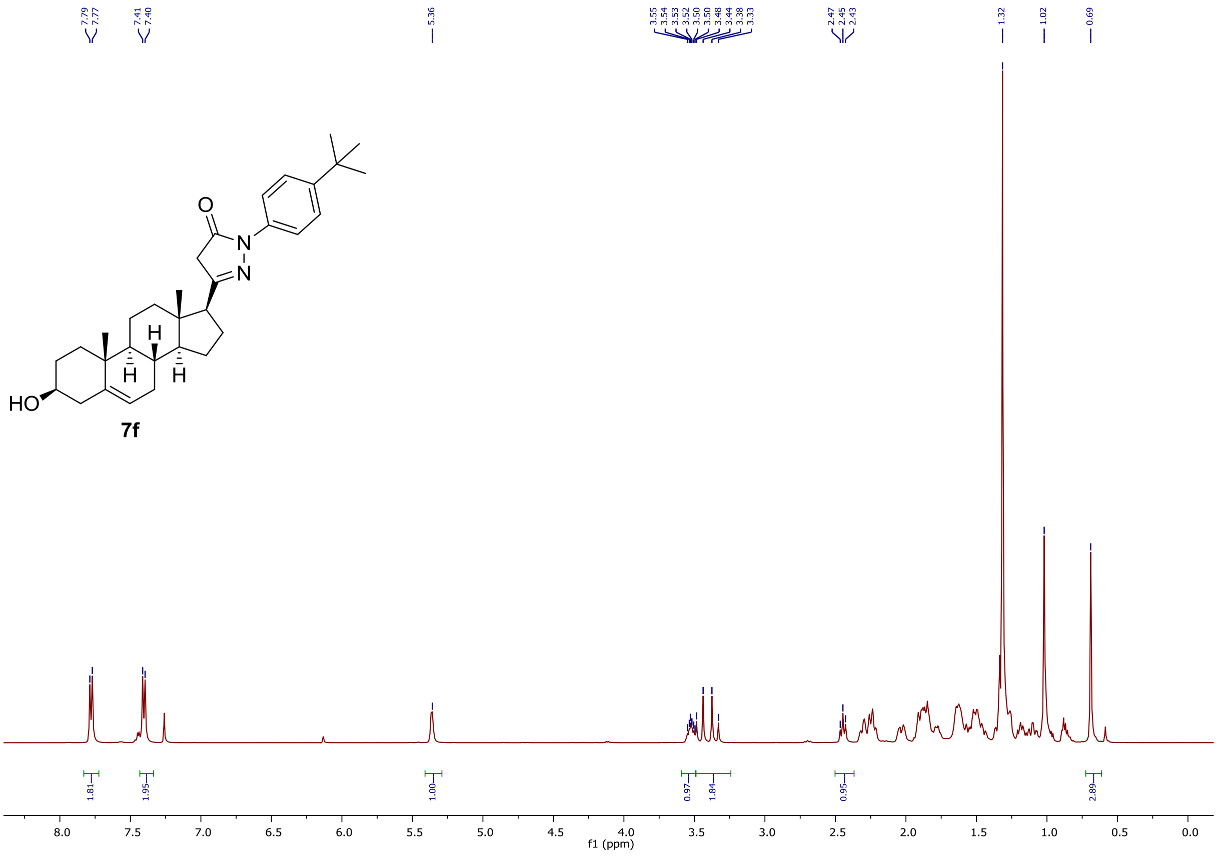
7.79
7.77
7.41
7.40
5.36
3.55
3.54
3.53
3.52
3.50
3.50
3.48
3.44
3.38
3.33
2.47
2.45
2.43
1.32
1.02
0.69
O
N
N
H
H
H
HO
7f
1.81
1.95
1.00
0.97
1.84
0.95
2.89
8.0 7.5 7.0 6.5 6.0 5.5 5.0 4.5 4.0 3.5 3.0 2.5 2.0 1.5 1.0 0.5 0.0
f1 (ppm)

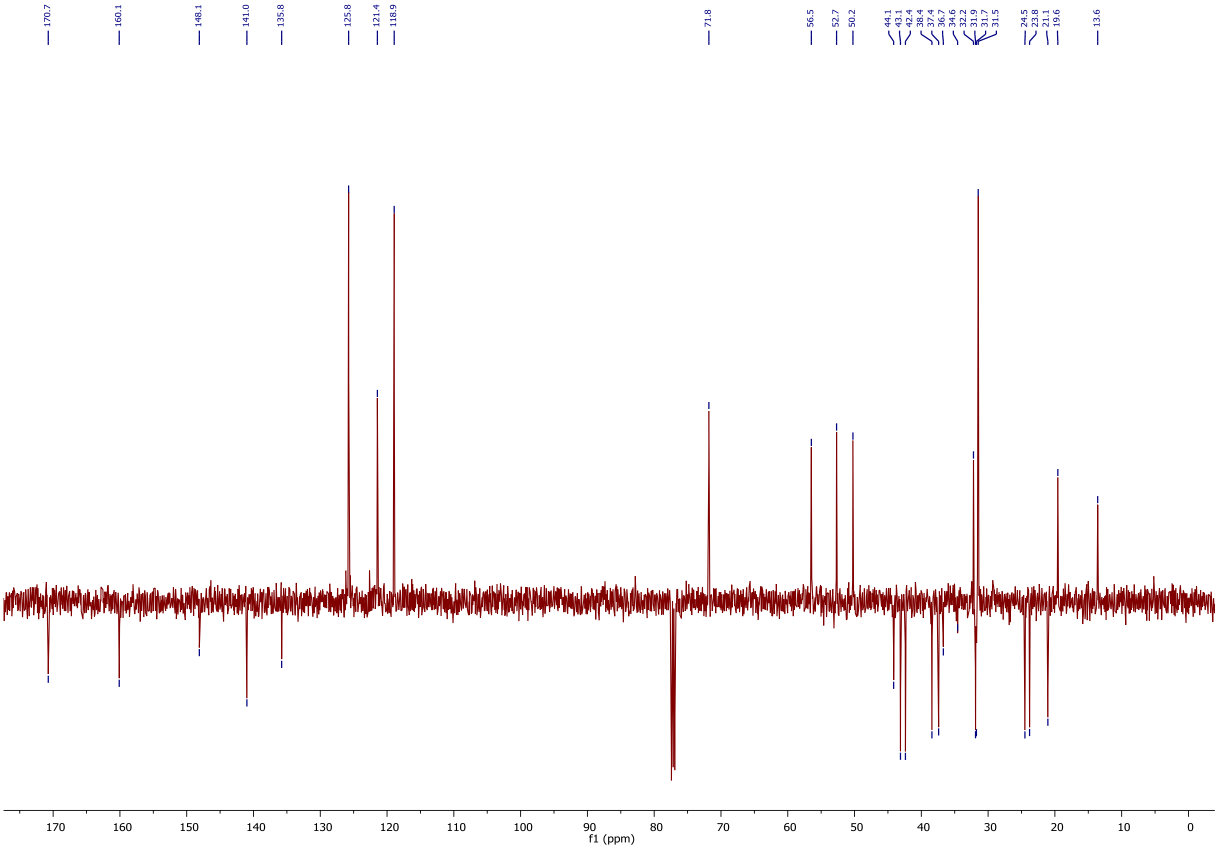
170.7
160.1
148.1
141.0
135.8
125.8
121.4
118.9
71.8
56.5
52.7
50.2
44.1
43.1
42.4
38.4
37.4
36.7
34.6
32.2
31.9
31.7
31.5
24.5
23.8
21.1
19.6
13.6
170 160 150 140 130 120 110 100 90 80 70 60 50 40 30 20 10 0
f1 (ppm)

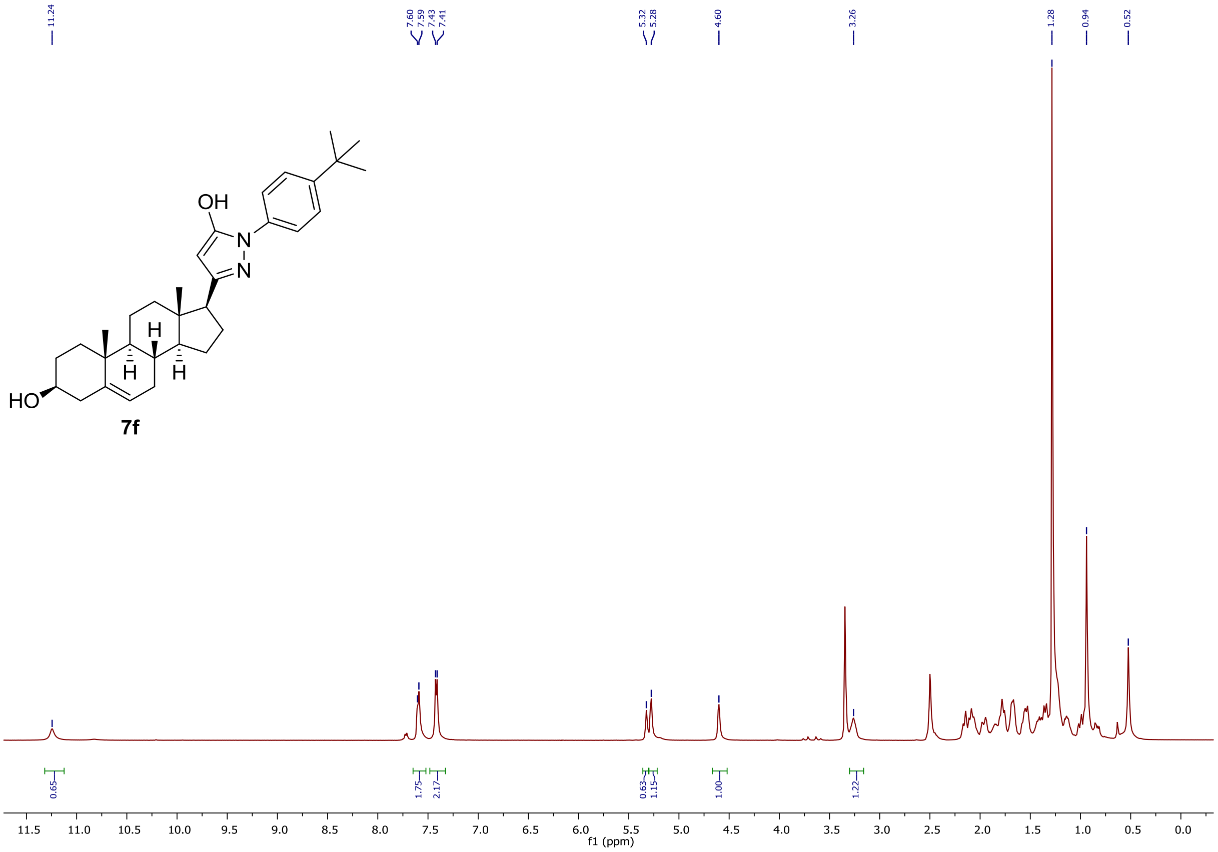
11.24
7.60
7.59
7.43
7.41
5.32
5.28
4.60
3.26
1.28
0.94
0.52
OH
N
N
H
H
H
HO
7f
0.65
1.75
2.17
0.63
1.15
1.00
1.22
11.5 11.0 10.5 10.0 9.5 9.0 8.5 8.0 7.5 7.0 6.5 6.0 5.5 5.0 4.5 4.0 3.5 3.0 2.5 2.0 1.5 1.0 0.5 0.0
f1 (ppm)

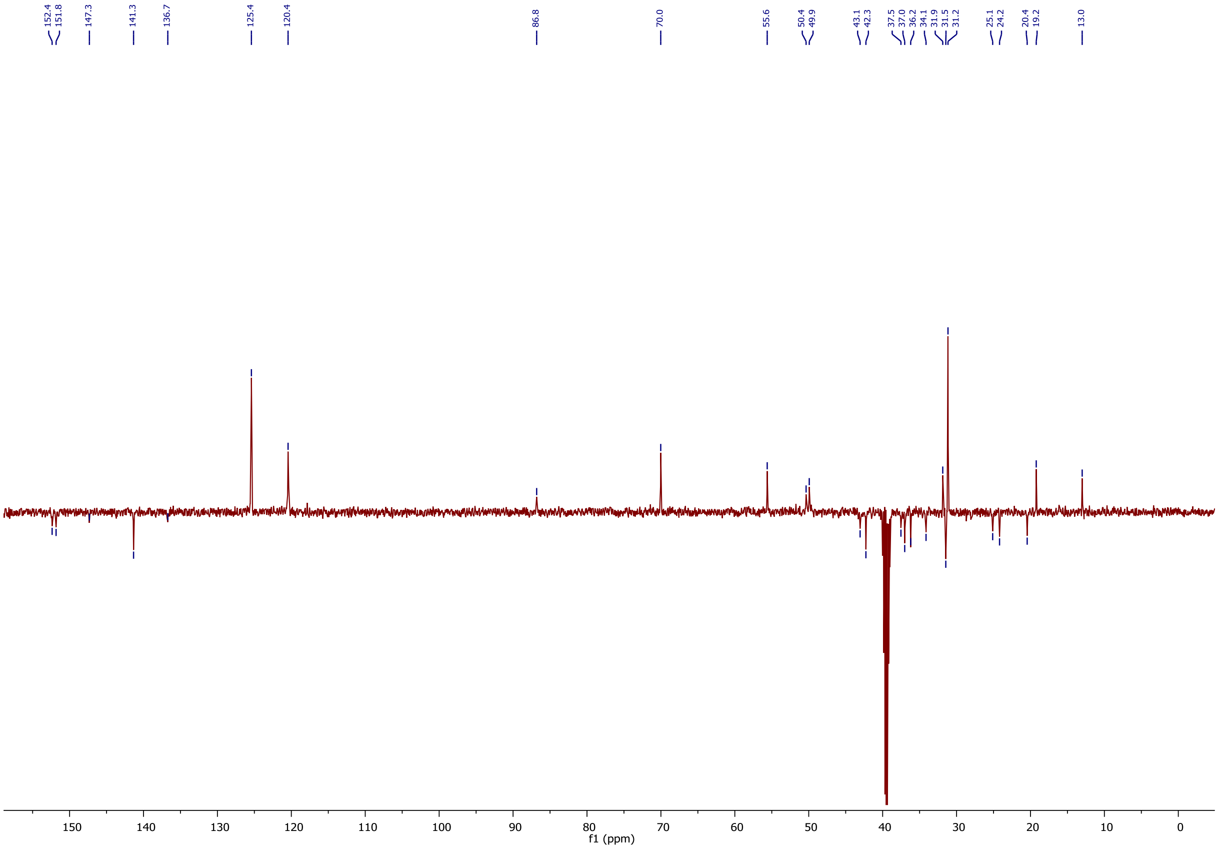
152.4
151.8
147.3
141.3
136.7
125.4
120.4
86.8
70.0
55.6
50.4
49.9
43.1
42.3
37.5
37.0
36.2
34.1
31.9
31.5
31.2
25.1
24.2
20.4
19.2
13.0
150 140 130 120 110 100 90 80 70 60 50 40 30 20 10 0
f1 (ppm)

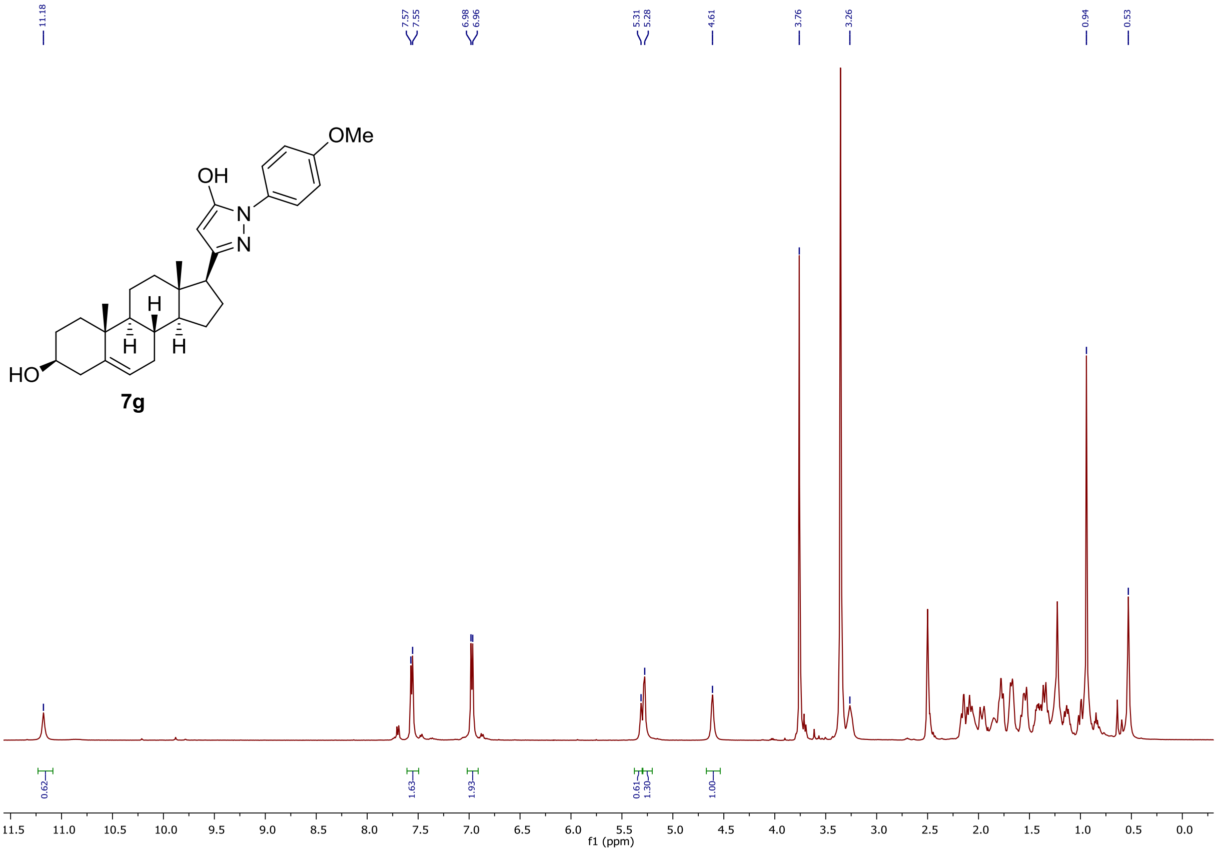
11.18
7.57
7.55
6.98
6.96
5.31
5.28
4.61
3.76
3.26
0.94
0.53
OMe
OH
N
N
H
H
H
HO
7g
0.62
1.63
1.93
0.61
1.30
1.00
11.5 11.0 10.5 10.0 9.5 9.0 8.5 8.0 7.5 7.0 6.5 6.0 5.5 5.0 4.5 4.0 3.5 3.0 2.5 2.0 1.5 1.0 0.5 0.0
f1 (ppm)

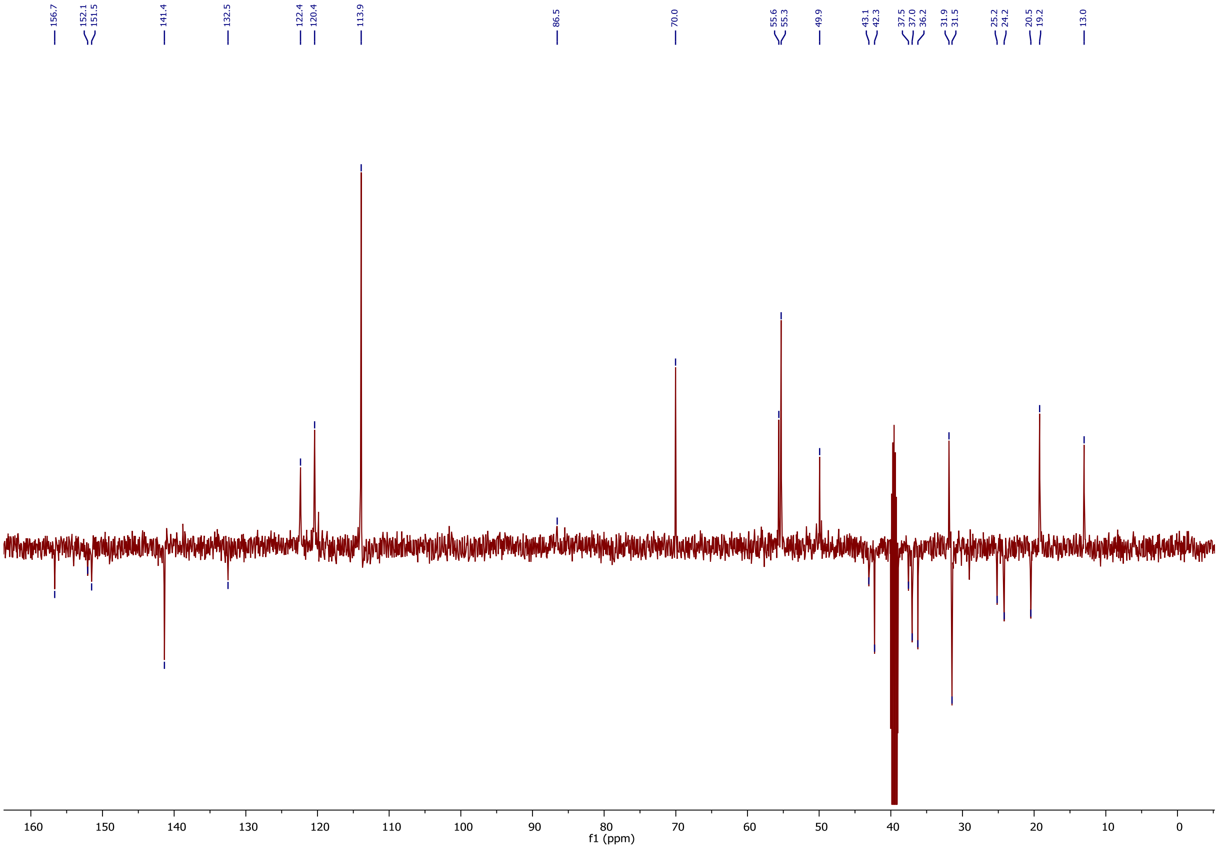
156.7
152.1
151.5
141.4
132.5
122.4
120.4
113.9
86.5
70.0
55.6
55.3
49.9
43.1
42.3
37.5
37.0
36.2
31.9
31.5
25.2
24.2
20.5
19.2
13.0
160 150 140 130 120 110 100 90 80 70 60 50 40 30 20 10 0
f1 (ppm)

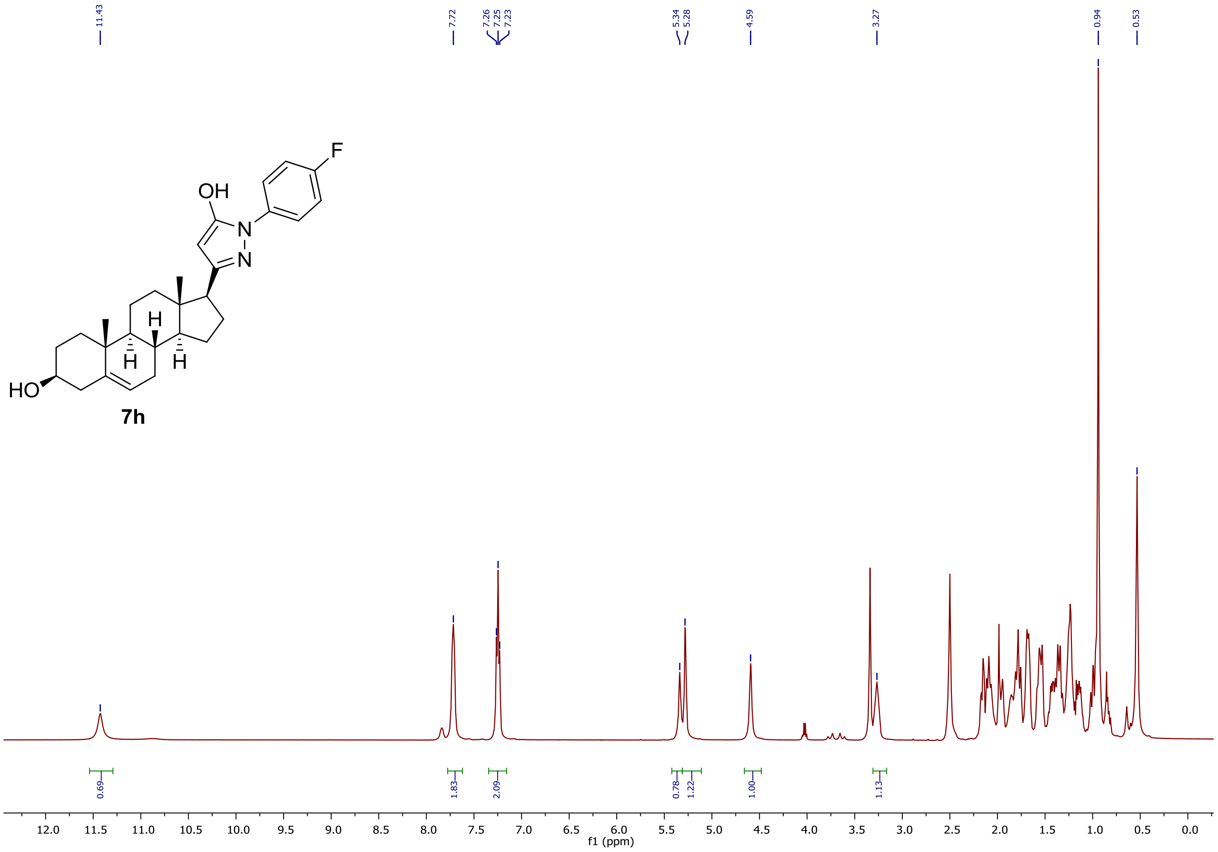
OH
N
N
F
H
H
H
HO
11.43
7.72
7.26
7.25
7.23
5.34
5.28
4.59
3.27
0.94
0.53
0.69
1.83
2.09
0.78
1.22
1.00
1.13
12.0 11.5 11.0 10.5 10.0 9.5 9.0 8.5 8.0 7.5 7.0 6.5 6.0 5.5 5.0 4.5 4.0 3.5 3.0 2.5 2.0 1.5 1.0 0.5 0.0
f1 (ppm)

7h

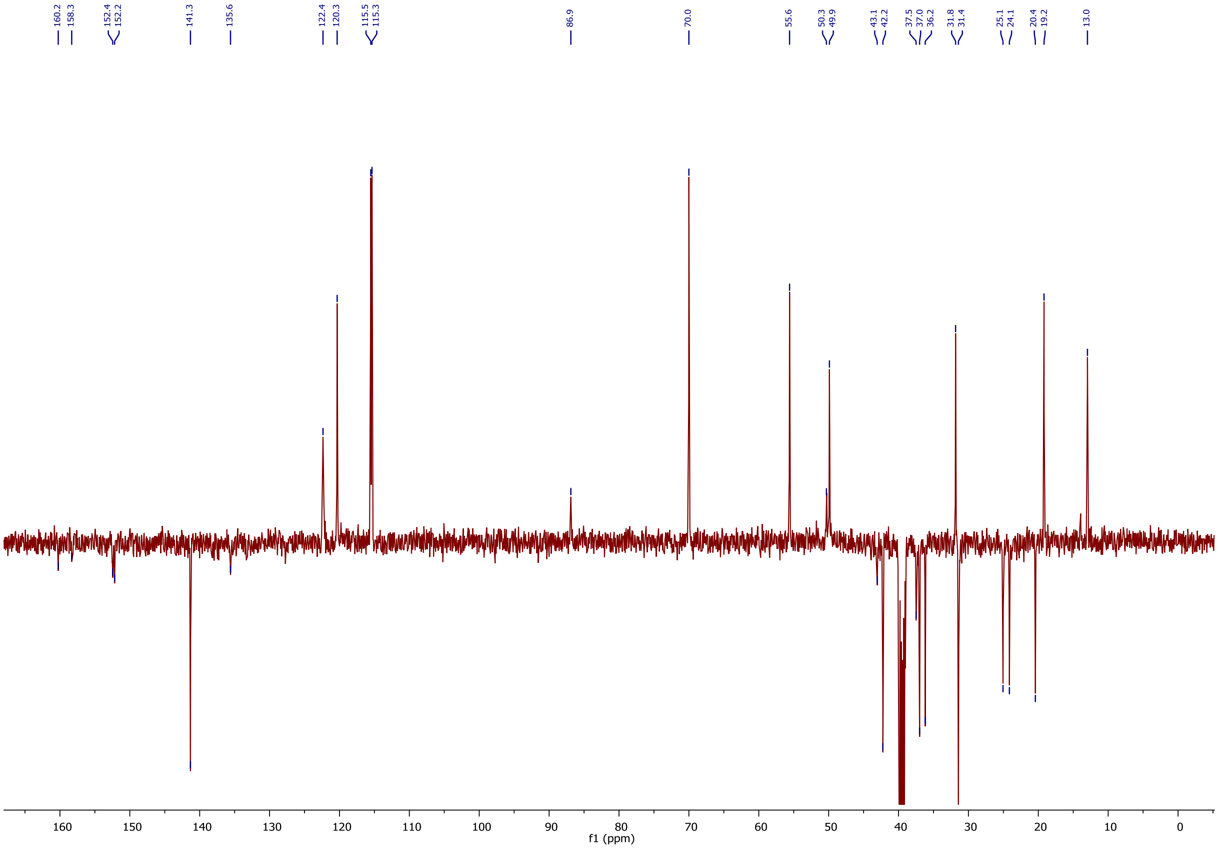
160.2
158.3
152.4
152.2
141.3
135.6
122.4
120.3
115.5
115.3
86.9
70.0
55.6
50.3
49.9
43.1
42.2
37.5
37.0
36.2
31.8
31.4
25.1
24.1
20.4
19.2
13.0
160 150 140 130 120 110 100 90 80 70 60 50 40 30 20 10 0
f1 (ppm)

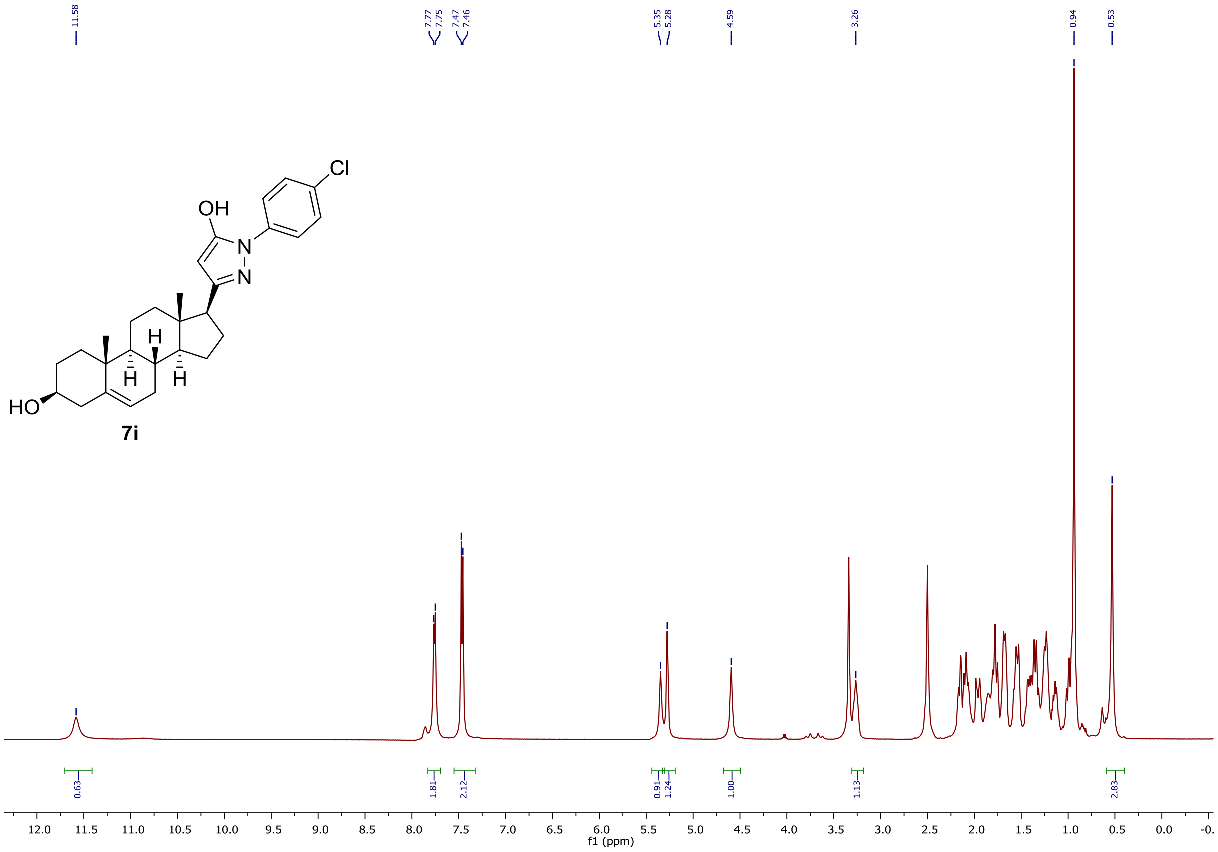
OH
N
N
Cl
HO
H
H
H
7i
11.58
7.77
7.75
7.47
7.46
5.35
5.28
4.59
3.26
0.94
0.53
0.63
1.81
2.12
0.91
1.24
1.00
1.13
2.83
f1 (ppm)

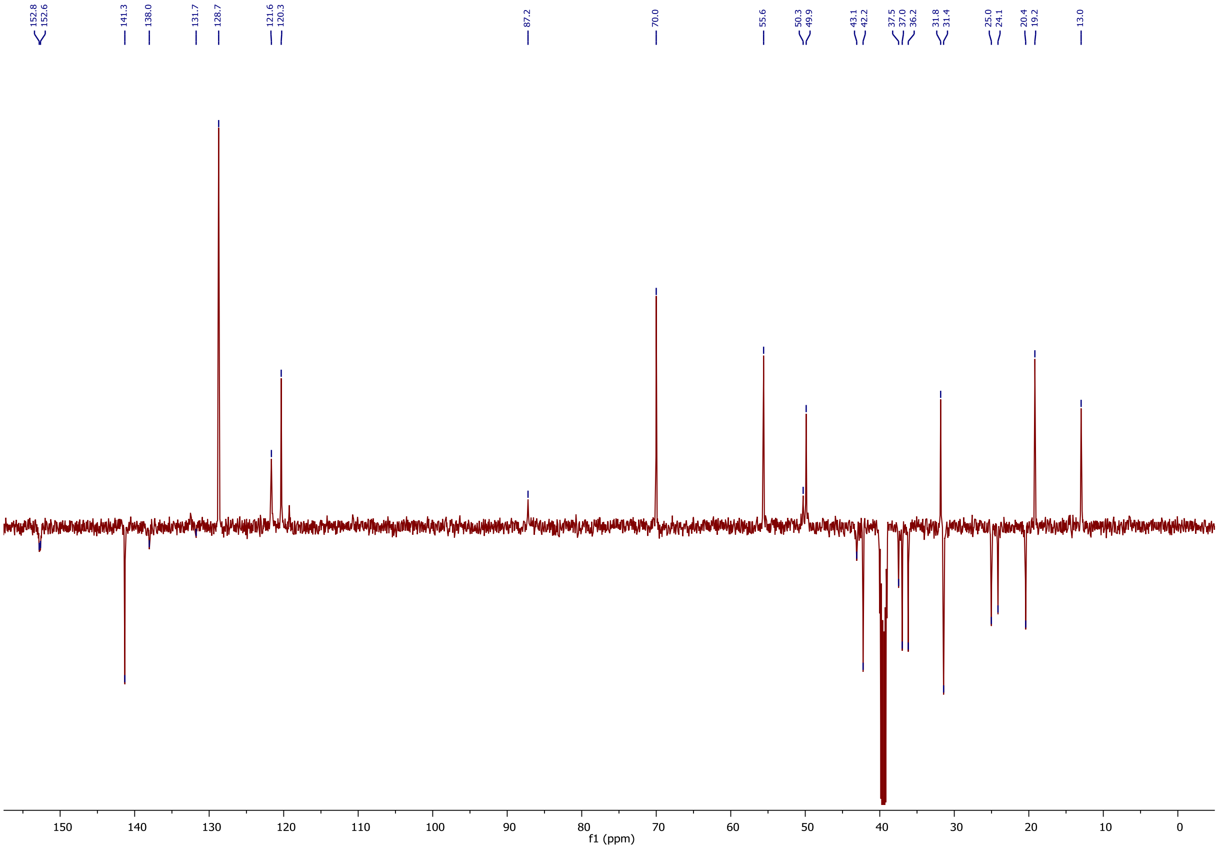
152.8
152.6
141.3
138.0
131.7
128.7
121.6
120.3
87.2
70.0
55.6
50.3
49.9
43.1
42.2
37.5
37.0
36.2
31.8
31.4
25.0
24.1
20.4
19.2
13.0
f1 (ppm)

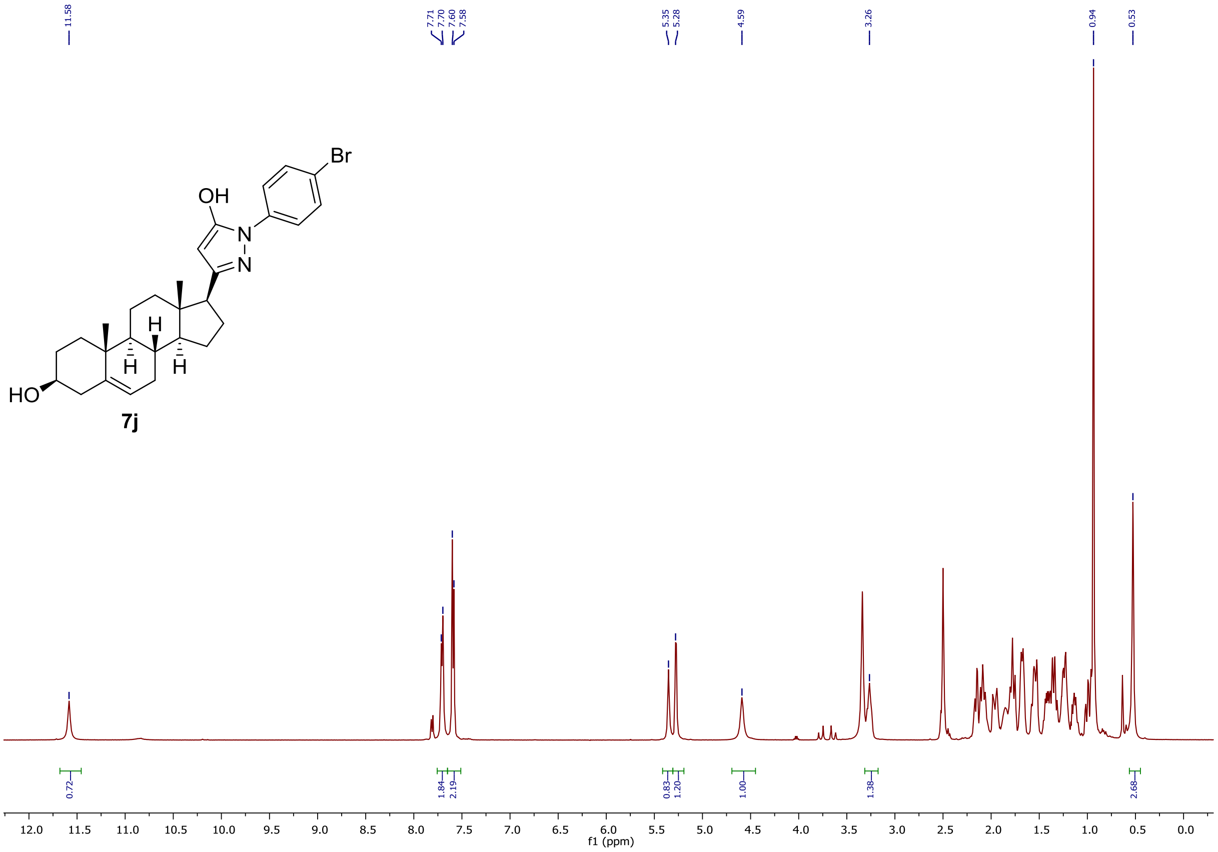
11.58
7.71 7.70 7.60 7.58
5.35 5.28
4.59
3.26
0.94
0.53
Br
OH
N
N
H
H
H
HO
7j
0.72
1.84 2.19
0.83 1.20
1.00
1.38
2.68
12.0 11.5 11.0 10.5 10.0 9.5 9.0 8.5 8.0 7.5 7.0 6.5 6.0 5.5 5.0 4.5 4.0 3.5 3.0 2.5 2.0 1.5 1.0 0.5 0.0
f1 (ppm)

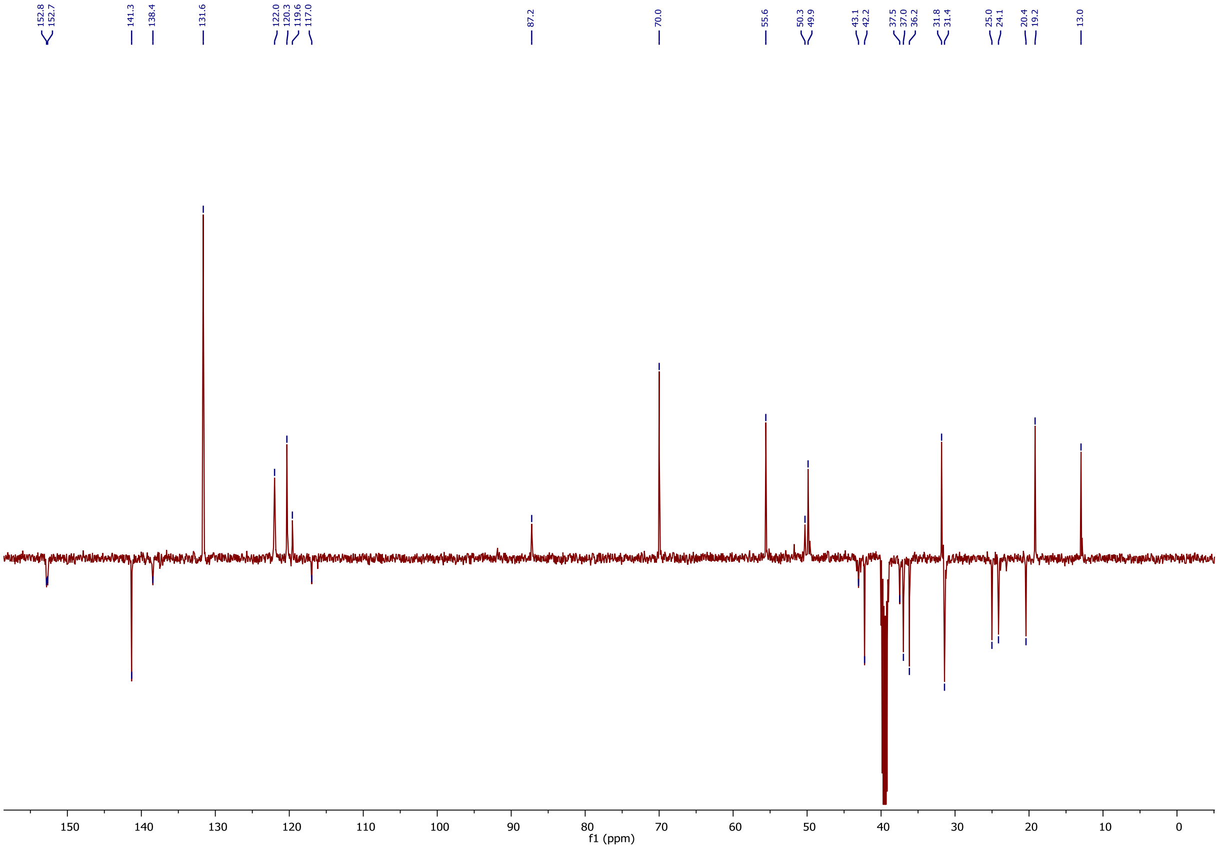
152.8 152.7
141.3
138.4
131.6
122.0 120.3 119.6 117.0
87.2
70.0
55.6
50.3 49.9
43.1 42.2
37.5 37.0 36.2
31.8 31.4
25.0 24.1
20.4 19.2
13.0
150 140 130 120 110 100 90 80 70 60 50 40 30 20 10 0
f1 (ppm)

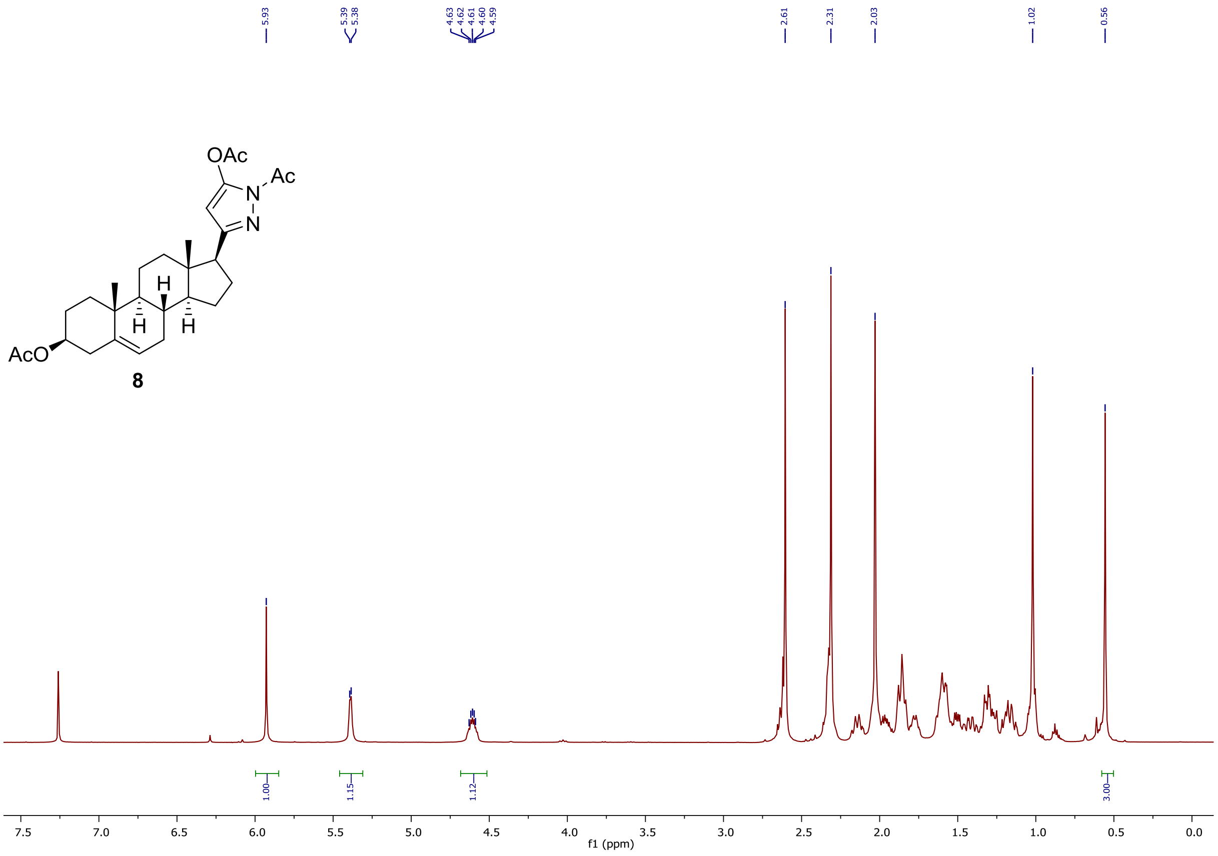
5.93
5.39
5.38
4.63
4.62
4.61
4.60
4.59
2.61
2.31
2.03
1.02
0.56
OAc
N
Ac
N
H
H
H
AcO
8
1.00
1.15
1.12
3.00
f1 (ppm)

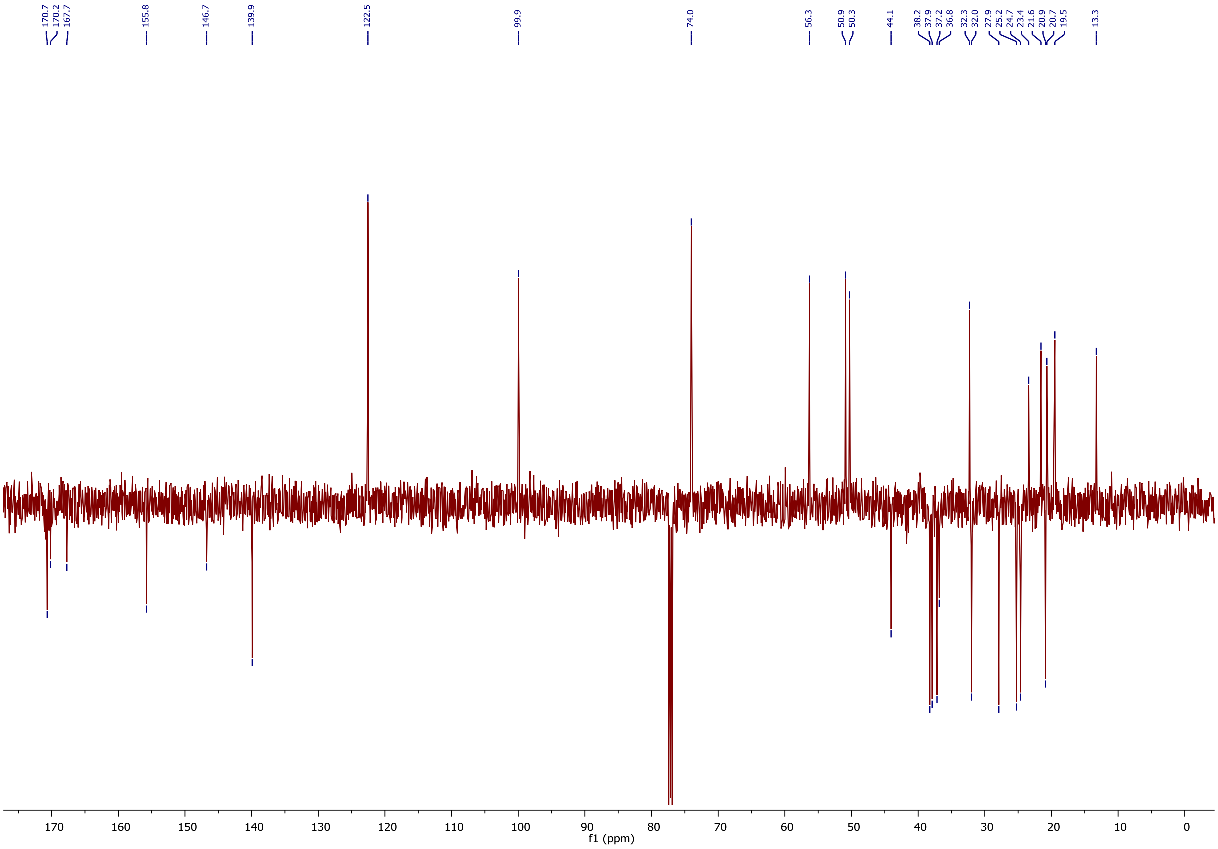
170.7
170.2
167.7
155.8
146.7
139.9
122.5
99.9
74.0
56.3
50.9
50.3
44.1
38.2
37.9
37.2
36.8
32.3
32.0
27.9
25.2
24.7
23.4
21.6
20.9
20.7
19.5
13.3
f1 (ppm)
